# Cropformer: A new generalized deep learning classification approach for multi-scenario crop classification

**DOI:** 10.3389/fpls.2023.1130659

**Published:** 2023-03-02

**Authors:** Hengbin Wang, Wanqiu Chang, Yu Yao, Zhiying Yao, Yuanyuan Zhao, Shaoming Li, Zhe Liu, Xiaodong Zhang

**Affiliations:** ^1^ College of Land Science and Technology, China Agricultural University, Beijing, China; ^2^ Key Laboratory of Remote Sensing for Agri-Hazards, Ministry of Agriculture and Rural Affairs, Beijing, China

**Keywords:** multi-scenario crop classification, time series, deep learning, pre-training, Cropformer

## Abstract

Accurate and efficient crop classification using remotely sensed data can provide fundamental and important information for crop yield estimation. Existing crop classification approaches are usually designed to be strong in some specific scenarios but not for multi-scenario crop classification. In this study, we proposed a new deep learning approach for multi-scenario crop classification, named Cropformer. Cropformer can extract global features and local features, to solve the problem that current crop classification methods extract a single feature. Specifically, Cropformer is a two-step classification approach, where the first step is self-supervised pre-training to accumulate knowledge of crop growth, and the second step is a fine-tuned supervised classification based on the weights from the first step. The unlabeled time series and the labeled time series are used as input for the first and second steps respectively. Multi-scenario crop classification experiments including full-season crop classification, in-season crop classification, few-sample crop classification, and transfer of classification models were conducted in five study areas with complex crop types and compared with several existing competitive approaches. Experimental results showed that Cropformer can not only obtain a very significant accuracy advantage in crop classification, but also can obtain higher accuracy with fewer samples. Compared to other approaches, the classification performance of Cropformer during model transfer and the efficiency of the classification were outstanding. The results showed that Cropformer could build up *a priori* knowledge using unlabeled data and learn generalized features using labeled data, making it applicable to crop classification in multiple scenarios.

## 1 Introduction

With a large amount of remotely sensed data available free, easily, and quickly, remote sensing plays an increasingly important role in vegetation and land cover mapping. The timely and accurate vegetation/land cover information generated by remote sensing images can provide important data for resource management, ecological monitoring, agricultural production, and other fields. For large-scale crop classification, continuous and full-coverage satellite images provided by remote sensing are particularly important (Chen et al., 2019; Zhang et al., 2020), and how to extract useful information for crop classification from satellite images has been explored. In addition, a large number of machine learning algorithms have been introduced to obtain large-scale crop distribution more accurately (Abdullah et al., 2019).

Full-season crop classification is the most common scenario in which current crop classification approaches are applied, but it is generally available at the end of the growing season. In-season crop classification allows the distribution of crops to be obtained as early as the growing season, which is of interest for agricultural production guidance, but few publicly available data are available(Xu et al., 2020). Current research has focused on classification approaches supported by large numbers of samples (Yi et al., 2020). Crop classification in a few-sample context is of interest in the crop classification scenario, where obtaining highly accurate classification results with very few samples can reduce costs. Crop classification in regions with no samples is difficult, and it is feasible to train a well-trained model in a sample-rich region and transfer it to a region with no samples (Hao et al., 2020). The existing crop classification methods all focus on one application scenario or two application scenarios, and there is no discussion of crop classification for multiple application scenarios, nor is there a general crop classification model that can be used. On this basis, developing a classification approach that can be applied to the above classification scenario is greatly needed.

Traditional crop classifiers include Decision Trees (DT), Random Forests (RF), and Support Vector Machines (SVM) (Low et al., 2013; Khatami et al., 2016; Shi and Yang, 2016). The inputs to these classifiers are usually manually designed features including spectral values, vegetation indices, etc., and multi-temporal satellite observations are used instead of mono-temporal imagery (Feng et al., 2019; Eudes Gbodjo et al., 2020). Although multi-temporal inputs are effective in improving classification performance, these classification models often ignore temporal dependence in the time series. Traditional methods require manual design of inputs, which need to be designed differently for different application scenarios. However, the manually designed features have some limitations and are very dependent on *a priori* knowledge and expertise, and the complex changes in realistic conditions affect the manually designed features more, which makes the classification models less robust and less generalizable.

In contrast to classical machine learning, deep learning no longer requires manually designed features, but can learn complex semantic features from high-dimensional data. Currently, deep learning has been widely used in agriculture due to its effectiveness, including crop classification (Kussul et al., 2017; Minh et al., 2018), pest and disease detection (Akbar et al., 2022; Shoaib et al., 2022a; Shoaib et al., 2022b), yield estimation(Nevavuori et al., 2019; Khaki et al., 2020), etc. The complex network structure of deep learning requires a large amount of labeled data for support, which creates difficulties for the agricultural fields where deep learning is applied. When the sample size is insufficient, it makes the model very easy to over fit and thus the application is much less meaningful. For agriculture, especially crop classification, the acquisition of labeled samples is not as easy as in computer vision, and each labeled sample acquisition is resource-intensive (Xu et al., 2020). Therefore, it is necessary to address the problem of deep learning in crop classification that requires the use of a large number of samples. Also, developing a generalized deep learning model that can use only a small number of samples and can be applied to other crop classification scenarios is a scientific challenge.

Multi-temporal observations add a more intensive focus on the phonological cycle of crop growth, but also bring the problem of not keeping the same interval between observations in the study area, which makes the acquired time series irregular. Irregular time series are not directly usable for RF, SVM, and need to be normalized to obtain regular time series. Standardization methods include the rejection of invalid spectral values (Abdullah et al., 2019), missing spectral value supplementation (Ienco et al., 2017; Kussul et al., 2017), and spectral value resampling (Liu et al., 2020; Wang et al., 2020), but this standardization process changes the original sequence information as well as increase the computational effort. Direct use of irregular time series can avoid the standardization process, but it also causes a decrease in classification accuracy. Dynamic Time Warping (DTW) has been used for the analysis of irregular time series, but its traversal algorithm can significantly increase the computational effort (Petitjean et al., 2012). The introduction of a Gaussian process to solve irregular time sampling and missing data is robust, but does not compare favorably with other methods in terms of classification performance (Constantin et al., 2021). The existing approaches using irregular time series usually involve two aspects. On the one hand, it starts from the time series itself, but this approach changes the original growth pattern of the crop, making it difficult for the model to learn the original growth pattern of the crop. On the other hand, it starts from the method of processing time series, which can directly use irregular time series but cannot form a complete system and will significantly increase the workload. Therefore, current methods either do not achieve satisfactory accuracy or do not allow the use of end-to-end classification methods.

The unlabeled data contain rich crop growth information, and unknown crop growth information can be used as *a priori* knowledge. Pre-training as an effective training method has been applied to land use classification to accelerate the convergence of the training process (Zhao et al., 2017). The self-supervised pre-training approach can improve the utilization of labeled samples in land cover classification (Tarasiou and Zafeiriou, 2022). Unlabeled data as pre-training data can effectively improve crop classification accuracy and reduce the use of labeled samples (Yuan and Lin, 2021; Yuan et al., 2022). However, pre-training is still less used in scenarios such as in-season crop classification and model transfer. At present, there is also no general pre-trained classification model that can be applied to multi-scenario crop classification.

This study aims to build a deep learning classification model that can be generalized in multi-scenario crop classification. The potential of a pre-trained classification model based on Transformer and Convolution structures for application in multi-scenario crop classification is evaluated. In this paper, we proposed a new deep learning approach, Cropformer, for multi-scenario crop classification. Full-season crop classification, in-season crop classification, few-sample crop classification, and model transfer experiments were set up in five study areas rich in crop types. A variety of best existing classifications were compared with different indicators. Our novel contributions are threefold:

1. A deep learning structure that fuses Convolution and Transformer is designed. The Transformer captures features throughout the reproduction period of the crop, while the convolution effectively utilizes information from key growth nodes of the crop. Combining the two features can improve the generalization ability of the model, which can be applied to multi-scenario crop classification.
2. The introduction of time-dimensional features on the input side of the model increases the diversity of the input. Position encoding has been added to solve the problem of unusable irregular time series due to missing values in remote sensing imagery.
3. Using a two-step classification framework and pre-training with unlabeled data increases the accumulation of crop growth knowledge in the model and improves the adaptability of the model in multi-scene crop classification.

The remainder of this article is organized as follows. Section II summarizes related work on crop classification. Section III provides a description of the remote sensing images and samples used in this paper. Section IV explains the motivation of the proposed method and describes the proposed network architecture. Section V reports the experimental results. Section VI discusses the article and presents future work. Finally, Section VII concludes this article.

## 2 Related work

An effective and general classification method is a prerequisite for achieving high accuracy crop classification. The more comprehensive the features extracted by the classification model, the more significant the advantages of the classification results obtained. According to the differences in feature selection strategies, existing crop classification methods can be classified into the following three categories.

*Supervised traditional classification methods* include machine learning classification models, such as SVM, RF, and Multilayer Perceptual (MLP). These models are sensitive to the spectral information of crops and use vegetation indices and spectral values as the main feature inputs. However, the sequence relationships hidden in the time series are not exploited, so more temporal features are incorporated in the model inputs including the statistical value of spectral value and vegetation indices and statistical features of vegetation indices curve (Pelletier et al., 2016; Zhang et al., 2018; Zeng et al., 2020; Liu et al., 2021). Comparing multiple crop vegetation index curves and obtaining key dates and key observations from the curves to distinguish crops can be effective in improving classification performance (Simonneaux et al., 2008; Lebourgeois et al., 2017). Many studies have used various functions to fit crop growth characteristics, including wavelet transform and double logistic function, and used the main parameters and significant stages of the functions as features for classification (Sakamoto et al., 2006; Soudani et al., 2008). Supervised traditional classification methods do not require a complex feature extraction process and the time cost is substantial. However, supervised traditional classification methods are influenced by their inputs as well as feature extraction strategies, and their application scenarios are single and cannot be adapted to multi- scenario crop classification.

*Supervised deep learning classification methods* include two outstanding algorithms Recurrent Neural Networks (RNN) and Convolutional Neural Networks (CNN) that can efficiently process sequential data (Ienco et al., 2017; Minh et al., 2018; Zhong et al., 2019). RNN has the unique advantage of processing sequential data, which is sensitive to temporal order (Mou and Zhu, 2018; Sharma et al., 2018; Papadomanolaki et al., 2019). Long Short-Term Memory (LSTM) model handles longer time series than RNN and has proven its effectiveness in capturing features in several classification models (Rußwurm and Korner, 2017; Zhong et al., 2019; Rajendran et al., 2020). The advantages of LSTM for inter-annual samples and the spatial transfer capability were also confirmed (Xu et al., 2020). LSTM has a drawback in parallel computation and cannot compute multiple layers at the same time, which can significantly increase the time consumption and is not practical for large-scale crop mapping. CNN has sparse swapping and parameter sharing, which may be able to reduce the time of network training (Tai et al., 2017). Different forms of input design have an important impact on classification performance for CNN (Marcos et al., 2018). From one-dimensional sequences, to two-dimensional images, to three-dimensional video streams have been used as input to CNN for hyperspectral or multispectral data classification (Chen et al., 2014; Kussul et al., 2017; Huang et al., 2018; Ji et al., 2018), but the conversion of multi-temporal observations to image or video streams increases the classification cost. In addition, a novel network structure combining RNN and CNN has been proposed to extract temporal features by learning temporal correlations (Mou et al., 2018; Martinez et al., 2021), but the features extracted by RNN and CNN are local features, which prevents the model from learning features from a global perspective. Therefore, the current supervised deep learning classification methods still have the drawback of extracting a single feature. Feature extraction is performed only from one side of crop growth, without combining key nodes of crop growth stages and the whole reproductive period, and it is impossible to obtain diverse features that can characterize crop growth patterns.

*Self-Supervised deep learning classification methods* have gained much attention because of its excellent learning ability on unlabeled data. The Transformer, consisting of multiple self-attention structures, is currently the most commonly used SSL structure (Vaswani et al., 2017; Devlin et al., 2018; Dosovitskiy et al., 2020). Xu et al. (Xu et al., 2020) verified that Transformer has advantages for processing time series, using parallel operations to overcome the problem of time-consuming processing of long time series, but experiments have only been conducted in in-season and full-season crop mapping. The Transformer is good at capturing global information but not sensitive to local information, so fusing the Transformer and CNN into a new network structure becomes a popular way (Gulati et al., 2020; Zhang et al., 2022). Li et al. (2020) developed a hybrid Convolution and Transformer network structure for multi-source remote sensing image classification and verified the feasibility of fusing the two, but it only discussed the effectiveness of the hybrid structure in full-season crop classification. However, the potential of the new network structure combining Transformer and CNN in the field of crop classification in other scenarios has not been fully tested. A general model with the ability to capture both global and local information is necessary for multi-scenario crop classification.

In conclusion, current crop classification methods still suffer from insufficient feature extraction ability, single application scenario, and lack of a general and effective classification method.

## 3 Materials

### 3.1 Study area

**Figure 1:**
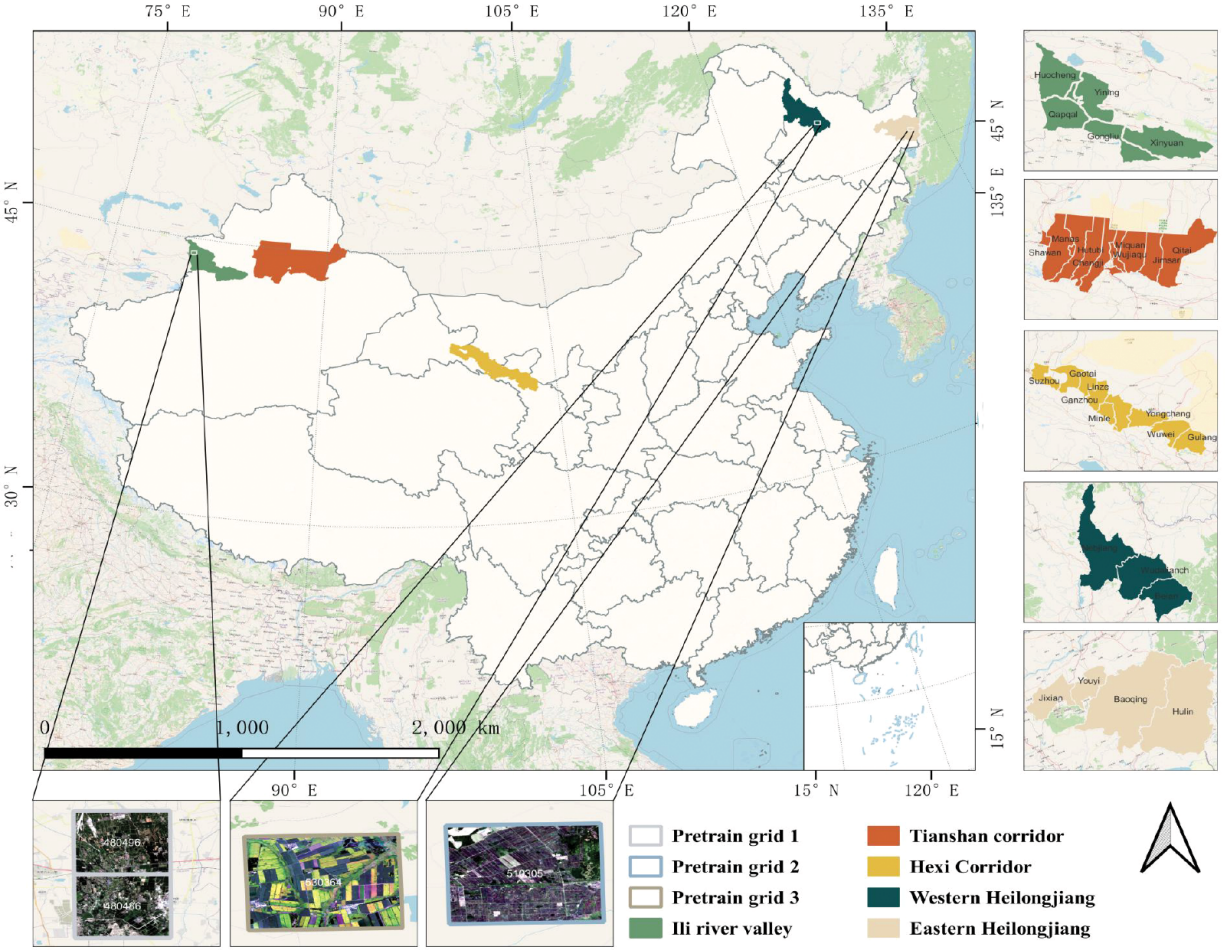
The geographical locations of the five study areas in China. The data used for pre-training are from the white boxed area in the Ili River Valley, Western Heilongjiang and Eastern Heilongjiang. The lower part shows a remote sensing image of GaoFen 1.

In this paper, three of the five study areas are located in Northwest China and two in Northeast China, as shown in **Figure 1**. The first study area is the Hexi Corridor, which is an important agricultural production area with abundant light resources and abundant snow and ice meltwater. The second study area is the Ili River Valley, which has a mild climate, a temperate continental climate, abundant sunshine and precipitation, and significant advantages for agricultural development. The third study area is the Tianshan Corridor, which is in the mid-temperate arid climate zone and is an oasis irrigated agricultural area. The fourth and fifth study areas are Western and Eastern Heilongjiang, which have a temperate continental climate and whose black land advantage makes them an important food supply base for China. These five regions have favorable agricultural production conditions and diverse crop types, which are ideal for verifying the validity as well as the robustness of the classification model.

### 3.2 Satellite imagery

The remote sensing images we used are acquired by the GaoFen-1 satellite, which contains four bands: red, green, blue, and near-infrared, with a spatial resolution of 16 m and a temporal resolution of 4 days. We only used images with cloud coverage of less than 10%, which causes inconsistencies in the length of the time series. The number of valid images for each study area is shown in **Table 1**, where Pre_His and Pre_Cur represent the imagery covering the pre-training sample areas acquired in the historical year and current year, respectively. **Table 1** shows that the number of remotely sensed images in each region and each month is different, and directly using the time series obtained from the images as input is a challenge for most models, which require pre-processing of the extracted time series. Cropformer, on the other hand, does not require any processing and can directly use the time series extracted from the images, which greatly improves the efficiency of the classification. The way of dealing with the irregular time series inputs by Cropformer is described in Section 4.1.

**Table 1:** Number of valid images in the five study areas.

	Study Area	Mar.	Apr.	May.	Jun.	Jul.	Aug.	Sep.	All
	I	12	7	5	3	6	8	4	45
	II	3	2	2	0	9	7	4	27
	III	7	6	5	7	12	8	8	54
	IV	1	5	2	4	3	1	8	24
	V	1	2	2	2	1	0	4	12
Irregular	Pre_His_IV	1	2	0	3	3	1	6	16
	Pre_His_V	1	1	1	1	0	0	2	6
	Pre_His_II	3	2	2	0	7	4	1	23
	Pre_Cur_II	0	2	2	6	2	0	0	12
	Pre_Cur_IV	2	1	0	2	2	6	6	19
	Pre_Cur_V	1	3	2	1	0	1	3	11
Regular	–	4	3	3	3	3	3	3	22

### 3.3 Sample dataset

Each of the three study areas of Northwest China has about 20 crop types in each region, and the main crops are maize, cotton, grape, and wheat. The two study areas in Northeastern China have a stable cropping structure, with the main crops being maize, rice, and soybean. We used unlabeled data from two 10 km × 10 km areas as the pre-training dataset, with a total of 781,250 sample units. In addition, field samples from three study areas collected in the current year were used for training and testing, where the ratio of the training dataset, validation dataset, and testing dataset was 6:2:2. The distribution of sample categories in the three study areas of Northwest China was very unbalanced, which also challenged our classification model. The numbers of sample units in each crop type are shown in **Table 2**.

**Table 2:** Number of Samples in the Three Study Areas.

Study area	Crop type	Number of samples	Study area	Crop type	Number of samples	Study area	Crop type	Number of samples
I	Seed Maize	36771	II	Middle Rice	27826	III	Cotton	301178
	Spring Maize	25136		Winter Wheat	8295		Spring Maize	78567
	Greenhouse	7659		Spring Wheat	97		Seed Maize	77360
	Woodland	4610		Spring Maize	221613		Grape	36779
	Alfalfa	3390		Seed Maize	61720		Tomato	27266
	Chili Pepper	3014		Sweet Potato	4008		Winter Wheat	15119
	Onion	2968		Safflower	1335		Sunflower	14561
	Winter Wheat	2493		Sunflower	10084		Gourd	10917
	Bare Land	2260		Soybean	9457		Bara Land	10691
	Grape	1925		Cotton	6446		Woodland	8222
	Sorghum	1796		Sugar Beets	10298		Chili Pepper	5939
	Stevia	1653		Stevia	7753		Silver Beet	5457
	Sunflower	1307		Alfalfa	6353		Watermelon	3027
	Spring Wheat	1189		Chili Pepper	2007		Sweet Potato	2252
	Pear	619		Gourd	1319		Greenhouse	1920
	Sweet Potato	308		Watermelon	950		Hops	1698
	Sugar Beets	223		Greenhouse	1641		Nursery	1636
	Soybean	186		Grape	2430		Spring Wheat	1248
	Gourd	142		Indian Jujube	2777		Alfalfa	805
	–	–		Woodland	13822		Pumpkin	381
	–	–		Nursery	5258		Potato	183
Total Number of Samples		97649			405489			605326
Study area	Crop type	Number of samples		Study area		Crop Type		Number of samples
IV	Soybean	1207		V		Soybean		571
	Spring Maize	440				Spring Maize		515
	Middle Rice	39				Middle Rice		416
	Other	364				Other		197
Total Number of Samples		2050						1699

## 4 Methods

### 4.1 Motivation

In this paper, we propose a new combined Transformer and Convolutional network structure, the new structure takes the Transformer as the main structure and is used for crop classification, thus naming the new structure a Cropformer.

**Figure 2:**
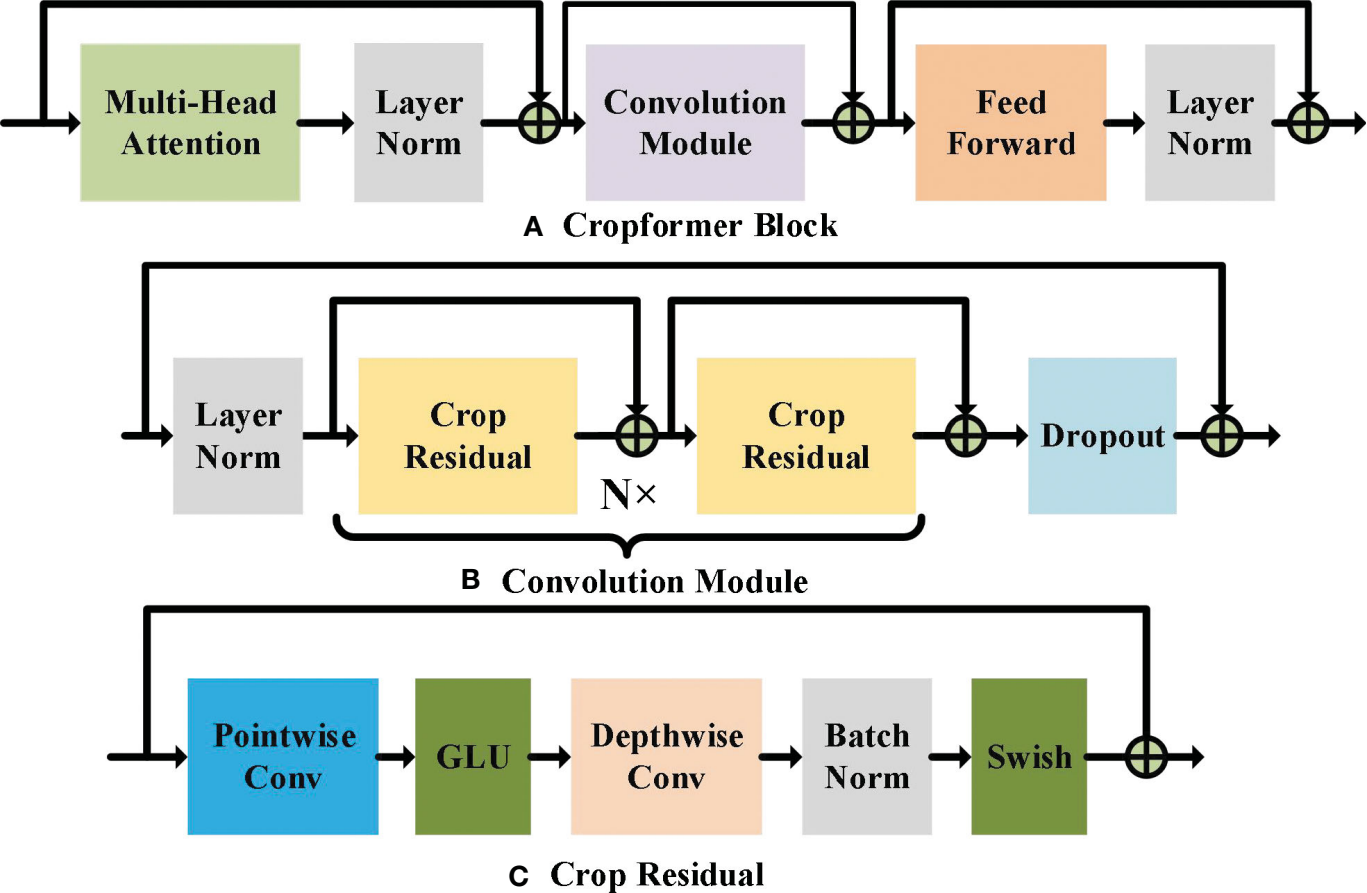
Cropformer Block, Convolution Module and Crop Residual detailed architecture. In **(A)** MHAL extracts global information, CM extracts local information, FFN will get information further enhanced, and LN is used for layer normalization to prevent model overfitting. **(B)** shows the two-start residual connection, and dropout is used to speed up the model training and prevent overfitting. GLU and Swish are the two learnable activation functions in **(C)**, and BN is used for batch normalization to prevent model overfitting.

Both the single Convolutional structure and the single Transformer structure have certain drawbacks for crop classification. Convolution focuses too much on the key issues local to the sequence and ignores the dependencies between long sequences (Vaswani et al., 2017). Although Transformer can learn the dependencies between long sequences, those of local information are insensitive (Liu et al., 2022). Fusing the two to achieve a complementary effect can improve the adaptability of the model to cope with crop classification in different scenarios. The new network structure uses an embedded structure (Devlin et al., 2018), as shown in **Figure 2B**, to embed the Convolutional part downstream of the multi-headed attention of the Transformer, so that the features with weights acquired by Transformer are input to the convolutional part, which can also be regarded as adding weight to the input of the Convolution, and thus the Convolution can pay more attention to those local features with larger weights. The Convolutional structure uses a new double-start residual connection, as shown in **Figure 2C**. This connection ensures that the original input with weights can be fed directly to the Feed Forward Layer without losing the information in the global features due to the addition of Convolutional modules, thus allowing the global information extracted by Transformer to be fused with the local information extracted by Convolution. We use a point-depth convolution structure (Hua et al., 2018) to solve the problem that adding convolution significantly increases the number of parameters in Convolution part, and we use a residual structure to allow the model to converge faster (He et al., 2016). In addition, we use two parameter-adjustable activation functions, GLU and Swish (Dauphin et al., 2017; Ramachandran et al., 2017), which ensure that the convolution structure can automatically select the best activation function for training based on the prior self-attention output, and both activation functions have the advantage of fast convergence, so that the whole model can be trained and converged more easily. Single convolutional structure and single Transformer structure have been very common in the field of crop classification, but methods combining these two structures are still less common in the field of crop classification. Therefore, building a classification model dominated by convolutional and Transformer structures is valuable in the field of crop classification.

Irregular time series are difficult to apply directly due to their irregularity and to solve this problem, we introduced positional encoding in Cropformer. The effectiveness of positional encoding in the field of natural language processing(NLP) has been demonstrated (Devlin et al., 2018), but there is no precedent in the field of crop classification as a method for solving irregular time series. Specifically, we encode the observation points of the acquired valid remote sensing images to form temporal features, which are fused with the spectral features and input into the model together. The model will learn the spectral features at the corresponding positions according to the temporal features, and no misalignment of spectral values will occur due to different sequence lengths.

Labeled sample data is costly to obtain, while unlabeled remote sensing image data is easily available, and using unlabeled data to improve the learning ability of the model is competitive compared to other models (Yuan and Lin, 2021; Yuan et al., 2022). Pre-training using unlabeled data in this study forces the model to learn the crop growth patterns from unlabeled data, thus accumulating a large amount of prior knowledge. Specifically, we randomly add noise to some of the nodes in the sequence of unlabeled data and pre-train the model using a self-supervised training approach, thus allowing the model to learn the spatial-temporal relationships of crops at different time nodes. This improves the generalization ability of the model as well as provides prior knowledge for supervised classification.

### 4.2 Cropformer

**Figure 3:**
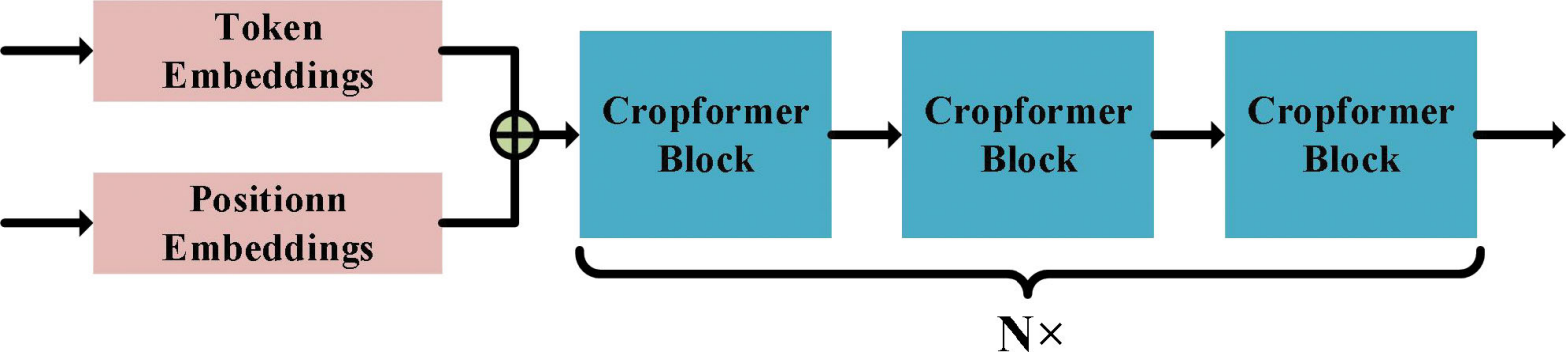
Cropformer architecture. Where Token Embedding denotes encoding of spectral information in time series and Position Embedding denotes encoding of temporal information in time series. Cropformer Block details can be shown by **Figure 2**, **4**.


The Cropformer architecture consists of three parts: Token Embedding (TE), Position Embedding (PE), and Cropformer Block (CB), whose architecture is shown in **Figure 3**. TE is a Linear Layer that projections the spectral sequence into a sequence feature vector of dimension *d*, i.e., equation (1). PE encodes the time series into a temporal feature vector of dimension *d* by equation (2). The sequence feature vector and the temporal feature vector are concatenated into a new vector as the input of CB.

(1) $$s_{i}=f\left(seq_{i}\right)$$

(2) $$t_{i}\left(p\right)=\{\begin{array}{c} \sin\left(doy_{i}/1000^{2k/d}\right)\;if\;p=2k\\ \cos\left(doy_{i}/1000^{2k/d}\right)\;if\;p=2k+1 \end{array}$$

(3) $$x_{i}=\text{Concat}\left(t_{i},s_{i}\right)$$

where *i*∈[0,*N*]*,k*∈[0,*d*] , *N* denotes the sequence (time series) length and *doy* represents the difference of valid sampling points. Encoding *doy* ensures that each growth node of the crop has a fixed temporal feature vector corresponding to it, so the input sequence can be irregular. Although irregular inputs can improve image utilization, they can lead to a very limited number of effective images acquired when subjected to practical conditions such as cloud occlusion, which requires the model to be able to learn key features from the constrained inputs.

CB in Cropformer can exist *N*, forming a network structure with depth *N*. However, there is a limit to the size of *N*, and infinite increase does not significantly improve the results, and its structure is shown in **Figure 2A**. The CB consists of three important components: Multi-Head Attention Layer (MHAL), Convolution Module (CM), and Feed-Forward Network (FFN). MHAL is good at capturing global information (Yuan and Lin, 2021), while CM can effectively use local features, and combining the two can achieve more comprehensive learning of crop growth and development.

**Figure 4:**
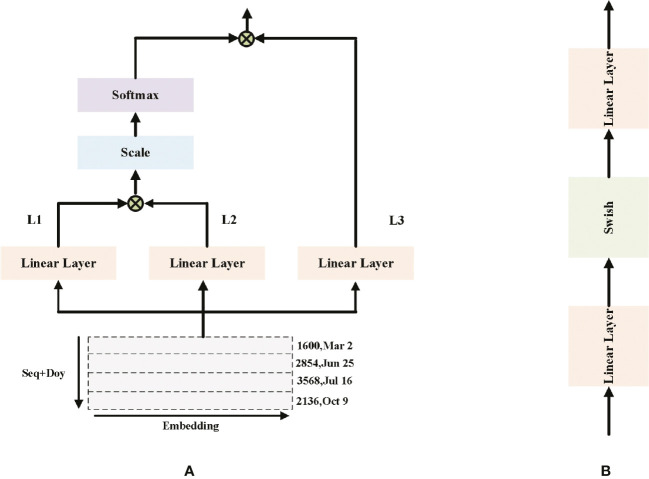
**(A)** Single MHAL architecture **(B)** FFN architecture. The inputs in **(A)** are the visualization results of the time series. L1-3 denote the three Linear Layers, but their weights are not the same.


**Figure 4A** shows that Single MHAL architecture. MHAL takes the joint vector of sequence + doy as input, by performing three linear projections on the input vector. The outputs of the first two Linear Layer are selected for scaled dot product and the fraction of each feature is calculated using Softmax, i.e., Equation. (4). The obtained feature scores are dotted multiplied once more with the output of the third projection, and finally the output of MHAL is obtained. MHAL is to calculate the feature scores between different positions of sequences, it learns the dependencies between sequences and obtains the global sequence information. The weight of the important sequence information in this part is scaled up, making more emphasis on this part in the CM part.

(4) $${\hat{x}}_{i}=soft\text{max}\left(\frac{L_{1}\left(x_{i}\right)L_{2}{\left(x_{i}\right)}^{T}}{\sqrt{d}}\right)L_{3}\left(x_{i}\right)$$

where *L_j_* represents *j*-th Linear Layer in MHAL; *d* represents the feature vector of dimension; *x_i_* denotes model input, and ${\hat{x}}_{i}$ denotes MHAL output. Softmax is used to calculate the score of each feature.

**Figure 4B** shows that FFN architecture. FFN is used to enhance the expressiveness of the features and consists of two linear layers and an activation function.

CM is composed of Layer Normalization (LN) (Ba et al., 2016), Crop Residual (CR), and Dropout (Srivastava et al., 2014), whose structure is shown in **Figure 2B**. The mathematical expression of the CB part is


(5) $$\begin{array}{l} {\tilde{x}}_{i}=x_{i}+LN({\hat{x}}_{i})\\ {x^{\prime }}_{i}={\tilde{x}}_{i}+CM({\tilde{x}}_{i})\\ y_{i}={x^{\prime }}_{i}+LN(FFN({x^{\prime }}_{i})) \end{array}$$

where *LN* represents Layer Normalization, *CM* represents Convolution Module, and *FFN* represents Feed-Forward Network; ${\tilde{x}}_{i}$ denotes CM input, *x* ^′^ denotes *FFN* input, and y*_i_* denotes model input.

CR is a pure Convolutional structure, we use a new double-start Shortcut connection that can fuse two kinds of features, which can guarantee lossless fusion of global and local features. The depth-separable convolution layer is used in CR, which effectively reduces the number of parameters and ensures that the model can be trained faster. Two activation functions, GLU and Swish, are used in the Pointwise Convolution Layer and Depth Convolution Layer, respectively, and in the last Batch Normalization (BN) (Ioffe and Szegedy, 2015), which is more suitable for convolution operations, is added to one convolution layer, and its architecture is shown in **Figure 2C**.

### 4.3 Crop classification framework

**Figure 5:**
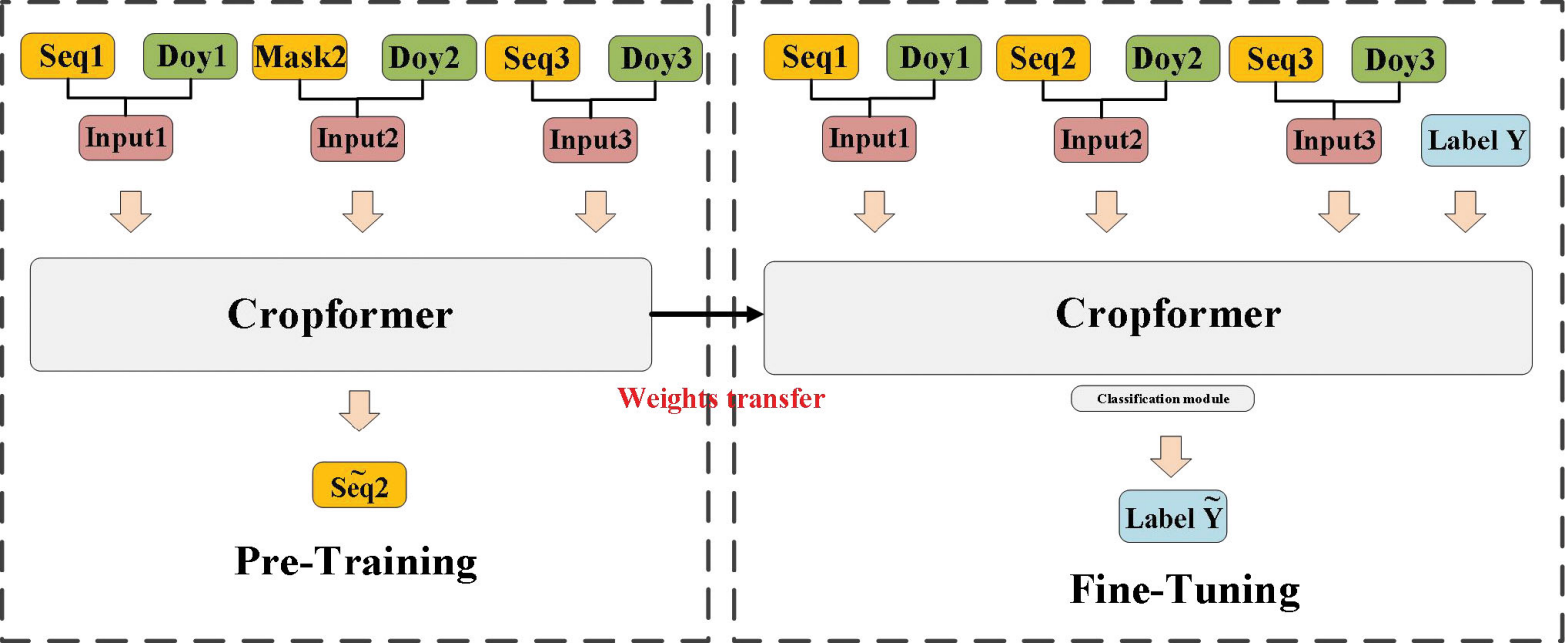
Cropformer crop classification framework. *Seq* represents the input time series, *Mask* represents the time series being masked, and *Doy* represents the sampling time point. The fine-tuning phase has the same network structure as the pre-training phase, but the fine-tuning phase has a Classification module.


The crop classification framework using Cropformer is divided into a pre-training part and a fine-tuning part, as shown in **Figure 5**. In the pre-training part, we use an SSL training approach for predicting missing values (Devlin et al., 2018), and it is worth noting that this part is trained entirely with unlabeled data. In the input continuous irregular time series, we randomize the time series of the MASK part of the input sequence sampling points, and Cropformer predicts the value of the MASK part by learning the spatial-temporal contextual relationships between the sequences, which allows the model to fully learn each node of crop growth and development. The loss function of Cropformer uses the Mean-Square Error (MSE) between the original and predicted sequences, i.e.

(6) $$Loss=\frac{\sum_{i=0}^{N}seq_{i}-s\tilde{e}q;}{N}$$

where *seq* represents the sequence value of MASK, $s\tilde{e}q$ represents the predicted sequence value and *N* denotes the number of masked sequence values. When the predicted values are infinitely close to the true values, it shows that Cropformer already can recognize the growth and development patterns of crops, thanks to pre-training using a large amount of unlabeled data. Although accurate crop types cannot be obtained by pre-training with unlabeled data, the growth patterns of a large number of crops expressed as time series are learned. These learned crop growth patterns are passed on to the fine-tuning stage as prior knowledge, thus ensuring that the best classification results can be obtained more efficiently in the fine-tuning stage.

After completing the pre-training, the resulting parameters from the pre-training part are transferred to the fine-tuning part for use. Because of the effectiveness of the pre-training part, the fine-tuning part does not take much time. Also, in the network structure part, only a simple classification module is added behind the Cropformer and then a supervised fine-tuning process is performed.

### 4.4 Experiment design and settings

**Figure 6:**
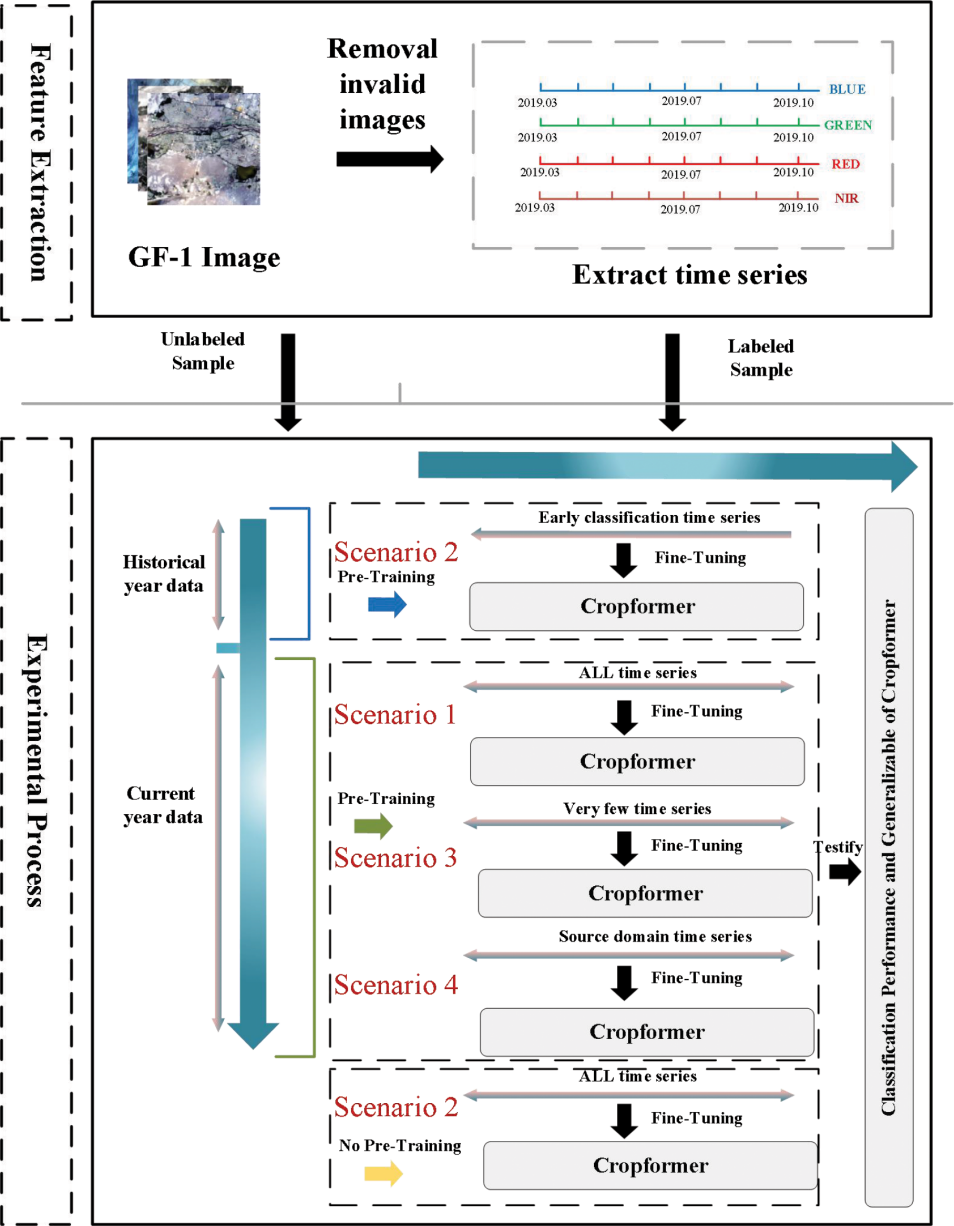
The experimental process uses historical data and current year data as pre-training data. The acquisition time of image data is from March to October. The bands used are blue, green, red, and near-red. The red serial number represents the number of crop classification scenario.

We had applied Cropformer in several crop classification scenarios to demonstrate its generality namely (1) Full-season crop classification; (2) In-season crop classification; (3) Few-sample crop classification, (4) Spatial transfer of classification model. The experimental scheme is shown in **Figure 6**. Specifically, (1) The classification ability of Cropformer was tested using current-year unlabeled data for pre-training and the full current-year field sample for fine-tuning, and compared to the best existing approaches RF (Pelletier et al., 2016), Res-18 (Thenmozhi and Reddy, 2019), SIFT-BERT (Yuan and Lin, 2021), Performer (Choromanski et al., 2020) and ALBERT (Lan et al., 2019); (2) In the in-season crop classification experiments, historical data were used as a pre-training data source, and the field sampling data in the current year were divided by month as fine-tuned data, e.g., March-end of April for the first stage of early detection and March-end of May for the second stage, with the input time series gradually becoming longer until all-time series were included; (3) In the few-sample crop classification experiment, two few-sample scenarios were simulated as balanced and unbalanced crop distributions. We designed experiments with 1% of labeled samples drawn from each class and a fixed number of labeled samples drawn from each class (the number of samples with the lowest number of all classes as the number of draws) as the fine-tuning training dataset; (4) Transfer learning can solve the problem of insufficient labeled samples in the target domain, and we set up two transfer methods in this experiment. One is to transfer the model from training in a region with sufficient samples to the target region, and the other was to transfer the model from training in a geographically similar region to the target region.

To evaluate the experimental results, besides the visually comparison of the classified crop maps, Overall Accuracy (OA), Average Accuracy (AA), and F1 scores were used to quantify the classification performance of different methods. In addition, all the results were the average of the three experimental results.

The hyperparameter settings of Cropformer are divided into two parts: network structure and training optimization, both of which are closely related to the performance of the model. For the network structure, the number of CB is set to 3 and the number of CR is set to 2. The number of Head in MHAL is set to 8 and the dimension of Linear Layers is set to 256; the dimension of Linear Layers in FFN is set to 1024; the size of convolutional kernels in CR is set to 7×7, padding is 3, stride is 1. The number of channels in both Pointwise Conv and The number of channels of both Pointwise Conv and Depthwise Conv is 256. For the training optimization, the pre-training stage was performed with 200 epochs, batch size set to 512, set to initial learning rate set to 1e-4, decay after 10 epochs, dropout set to 0.1, and the optimizer is selected as Adam; the fine-tuning stage was performed with 10 epochs, batch size set to 256, learning rate set to 1e-5, and dropout set to 0.1. The input size of the model can be obtained from equation (7)


(7) input_shape=t_num*band_num+t_num

where *t_num* denotes the number of images acquired which is the length of the time series, and *band_num* denotes the number of bands. The number of bands in this paper is 4, but the inputs to the model in this paper are diverse because the inputs used are irregular time series.

The entire experiment was run on a Windows platform configured with an i7-11700 K @ 3.60 GHz, 32 G RAM, and NVIDIA GeForce RTX 3080 GPU (10 GB RAM), and all programs were written using the python language.

## 5 Results and analysis

### 5.1 Full-season crop classification

In our experiments, we compared Cropformer with five competing methods. RF had advantages in processing sequential data and hence was used as a baseline for traditional machine learning algorithms. The advanced deep learning methods Res_18, SIFT_BERT, Performer, and ALBERT were used as comparisons and they showed good results in crop classification as well as sequence data processing. Among them, the input of Res_18 was a three-dimensional tensor, the input of RF and Performer was a regular time series, and the input of SIFT_BERT and ALBERT were irregular time series in line with the input of Cropformer, and the experimental results were shown in **Table 3**.

**Table 3:** Performance comparison of cropformer and other classifiers in three study areas.

Study Area	Methods	RF	Res-18	Performer	SIFT_BERT	ALBERT	Cropformer
Study Area I	AA(%)	56.43	50.14	54.95	71.11	70.66	**73.16**
	OA(%)	80.72	73.08	74.10	79.65	78.58	**81.93**
Study Area II	AA(%)	48.16	34.73	60.33	63.62	63.31	**64.81**
	OA(%)	85.82	84.80	84.65	84.56	85.39	**86.32**
Study Area III	AA(%)	60.02	43.85	58.89	57.42	**62.37**	61.23
	OA(%)	85.61	78.29	83.69	83.97	**85.99**	85.70
Study Area IV	AA(%)	73.26	59.71	78.10	**82.91**	79.66	81.52
	OA(%)	79.71	71.71	81.46	82.44	83.49	**84.39**
Study Area V	AA(%)	53.92	48.45	60.86	**72.42**	70.92	70.23
	OA(%)	71.79	71.49	71.06	77.87	78.91	**79.15**

Bolded indicates best results.

**Table 3** showed that Cropformer obtained OA of 81.93%, 86.32%, 85.70%, 84.39%, and 79.15% in the five study areas, respectively. Compared to the baseline RF of the traditional method, the increase was more than 5% in study areas IV-V with fewer samples, while in study areas I-III with abundant samples, the increase was not significant. This demonstrated the saturation of the classification accuracy achieved by the classification algorithm when samples were sufficient. In study area IV, Cropformer obtained AA of 81.52%, which was very close to the OA (84.39%) obtained in this region, while in study areas II and III it obtained AA of 64.81% and 61.23%, respectively, which was more than 20% different from the OA (86.32%, 85.70%) obtained, and this difference was more pronounced in RF (37%, 25%). This was due to the extremely unbalanced distribution of samples in study area II and III, where the sample size of maize was more than half of the total sample size, causing the AA to be insignificant, which is consistent with the findings of (Wang et al., 2022). Sample imbalance can bias the classification results toward the more numerous categories. The results showed that Cropformer in full-season crop mapping enabled to achieve better and more stable classification results even in situations where the sample conditions were not ideal, while the performance of the traditional classification method (RF) was not stable and vulnerable to realistic conditions, which is in line with the conclusions of (Xu et al., 2020).

SIFT-BERT and ALBERT outperformed Cropformer for classification in a few cases. ALBERT achieved AA/OA of 62.37%/85.99% respectively, which was better than Cropformer (61.23%/85.70%); SIFT-BERT achieved AA of 82.91%/72.42% in study areas IV-V, again better than Cropformer (81.52%/70.23%). However, in most cases Cropformer had a clear advantage in both AA and OA. In study areas II, III, V, where the number of valid images was low (valid images of 27/24/12, respectively), Cropformer had a 1%-8% improvement in OA compared to RF and Performer (22 valid images) using regular time series, while the improvement in AA was very significant (3%-16%). This indicated that Cropformer is able to learn more useful features from a finite length sequence. In fact, regular time series required resampling operation, and in the case of limited number of images, this operation would destroy the original information of the time series and thus had an impact on the classification results. Res_18 performed the worst among all methods, obtaining only OA of 84.80% in study area II, and no more than AA/OA of 60%/80% in other study areas, which was an unacceptable result. Although the Res_18 used a more informative three-dimensional tensor as input, its classification accuracy was not outstanding. Therefore, the direct use of one-dimensional time series as input results as well as efficiency would be more advantageous.

**Figure 7:**
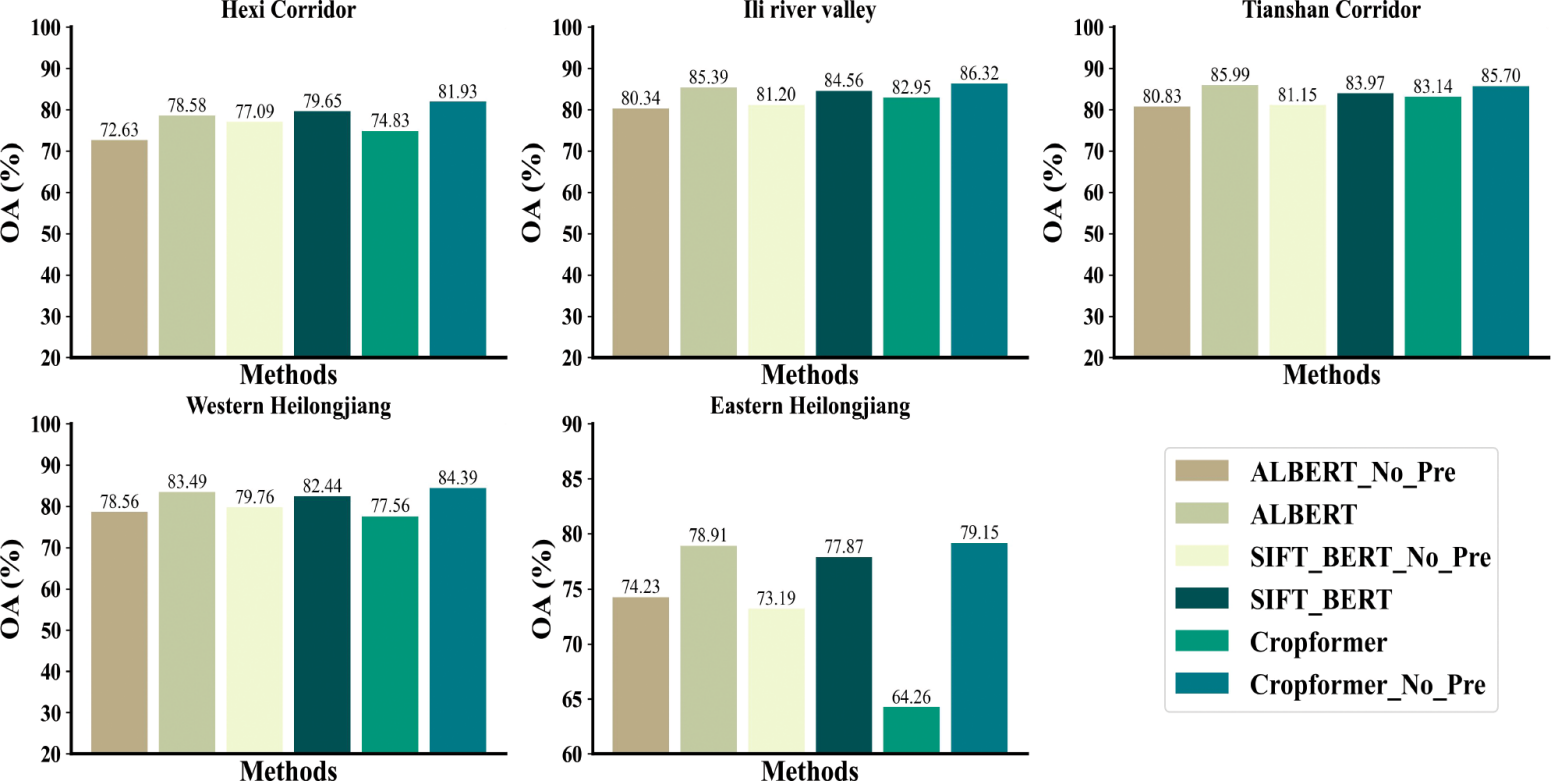
Performance comparison of Cropformer and other classifiers in five study area.

**Figures 7B, C** showed that in the case of sufficient samples, all three methods had a significant improvement in accuracy after pre-training, among which ALBERT had the most significant improvement (5%), while Cropformer and SIFT-BERT do not have a significant increase (2%-3%). The accuracy improvement of the three methods after pre-training was very obvious, especially in study areas IV-V, where the sample size was very small, and the highest improvement was up to 14.89%. The results showed that pre-training not only improves the crop classification accuracy, but also effectively reduced the model’s demand for samples, which was consistent with the findings of (Yuan and Lin, 2021; Yuan et al., 2022). In addition, the the average improvement in the five study areas of the Cropformer (6.95%) after pre-training was higher than that of BERT (3.22%) and ALBERT (5.2%), which indicated that the Convolution-Transformer structure in the Cropformer had better learning ability compared to the single-structured Transformer. By focusing on both global and local crop growth patterns, we could not only focus on the key growth nodes of crops but also capture the dynamics of crops throughout the reproductive period, and combine two important discriminatory approaches to better distinguish between different types of crops.

### 5.2 In-season crop classification

**Figure 8:**
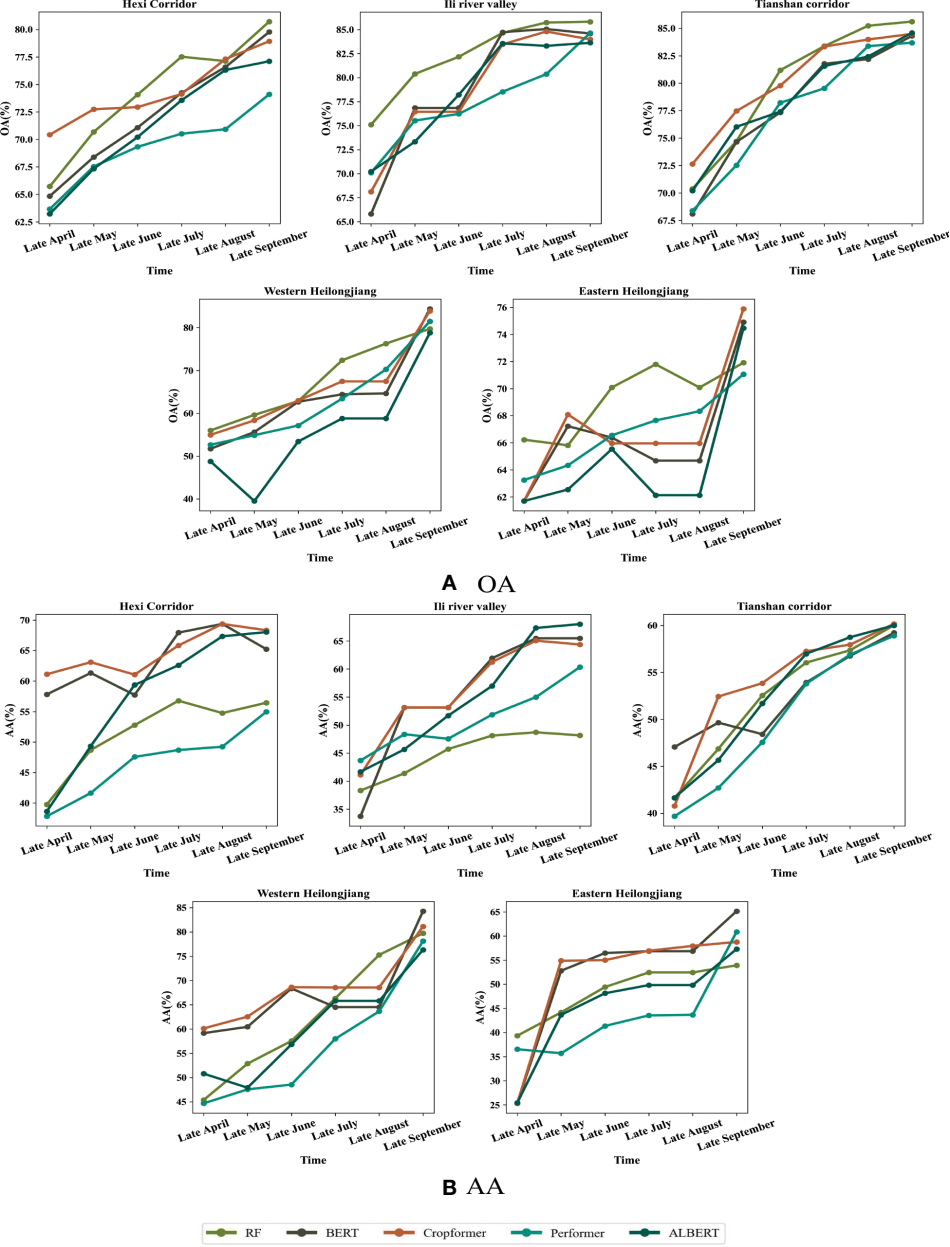
Early detection results with time transformation in the five study areas. The first row of which is OA and the second row is AA for the period from the end of April to the end of September. **(A)** OA for in-season crop classification **(B)** AA for in-season crop classification.

Historical unlabeled data were selected as pre-training data in the in-season crop classification experiment. We believed that crop growth information could be learned by training on historical data, even if it was not from the current year. Therefore, we used historical data as pre-training data in in-season crop classification and irregular time series of different lengths of the current year as fine-tuning data. The classification time was a one-month interval, with the end of April as the start time and the end of September as the end time, and the experimental results were shown in **Figure 8**.

**Figure 8A** showed Cropformer dominance in early crop growth (end of April - end of May), especially in study area I, III, V where OA of 72.74%, 77.47% and 68.09% were obtained, while RF only obtained 70.68%, 74.71% and 65.81%. However, the advantage of Cropformer was not obvious in the middle of the crop growth period (end of June - end of August), especially in study area II and study area V, where the number of valid images for this time period was extremely low, thus leading to the inability to obtain valid classification results using the irregular time series method. **Figure 8B** showed that the advantage of Cropformer in evaluating the in-season crop classification with AA Cropformer can improve 1%-6% in early crop growth (end of May) compared to other methods, while in mid-growth the performance was comparable to SIFT-BERT and ALBERT, but significantly better than RF and Performer, which was most evident in study area I and study area II, where the improvement was close to 10%. Comparing **Figures 8A, B**, it can be found that in in-season crop classification, although RF can achieve some advantage (when evaluated with OA), it was the classification of a large number of teste data into a larger number of categories that achieved better results, so that the classification results are no longer advantageous when evaluated with AA. The pre-training of the accumulated prior knowledge improved the ability of the model to detect different classes of crops, so the model with pre-training can be applied to areas with uneven sample distribution and complex crop types. In addition, the pre-trained data were derived from historical data, which would make the historical data as pre-trained data had an impact on the classification accuracy if the historical data were different from the current year’s data in terms of sowing time and other agricultural activities, resulting in some differences in crop growth stages from the current year.

**Figure 9:**
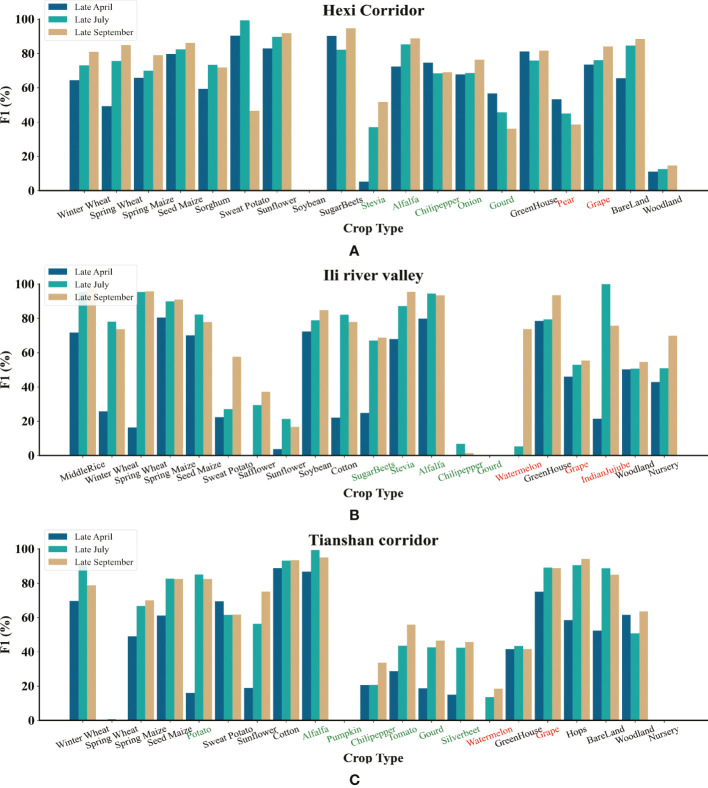
F1 scores for each crop type in the three study areas using Cropformer at the end of April, July and September. Where the green axis headings indicate vegetables and the red axis headings indicate fruits. **(A)** Hexi Corridor F1 Score **(B)** Ili River Valley F1 Score **(C)** Tianshan Corridor F1 Score.

The earlier the crops were classified, the more important the impact on agricultural production, so we further investigated the earliest point in time of the year when different crops were classified using Cropformer in three areas rich in crop types. Since different crops had different sowing times, the earliest time that could be classified would be different and similar. We analyzed the end of April and the end of July as two important points in the in-season crop classification, and the experimental results were shown in **Figure 9**.

**Figure 9** shows for half of the crops in all three study areas, more than 50% of the F1 scores were available at the end of April and nearly 60% of the crops had more than 70% of the F1 scores at the end of July, with a higher percentage in the Hexi Corridor(80% of the crop had an F1 score above 50% at the end of April and 70% of the crop had an F1 score above 70% at the end of July), due to the relatively balanced distribution of samples in the Hexi Corridor compared to the other two study areas. The unbalanced sample distribution was very unfair for crops with relatively small sample sizes, such as Chili Pepper and Gourd in the Ili River Valley, which were very susceptible to confounding with other crops (both had relatively low F1 scores in the three study areas), and because of the small number of available training samples, a large amount of confounding could occur. In all study areas, the F1 scores for each crop type at the end of July were very close to the F1 scores at the end of September, and even some crops had higher F1 scores at the end of July than at the end of September. When the crops were close to maturity or harvest, the time series information of crops was very close at this time, and if we continue to add time-series information, it would generate redundant information or useless information, which would make the model misclassify, so we could consider the end of July as a better time point for early classification using Cropformer. Hao et al. (2018)also proved the conclusion that accuracy consistent with crop maturity can be obtained in July-August. Cropformer uses irregular sequences and acquisition of very limited spectral information about crops but still allows crop identification at an earlier time, which shows that Cropformer can be applied to the classification of in-season crops in regions with complex crop types.

### 5.3 Few-sample crop classification

**Figure 10:**
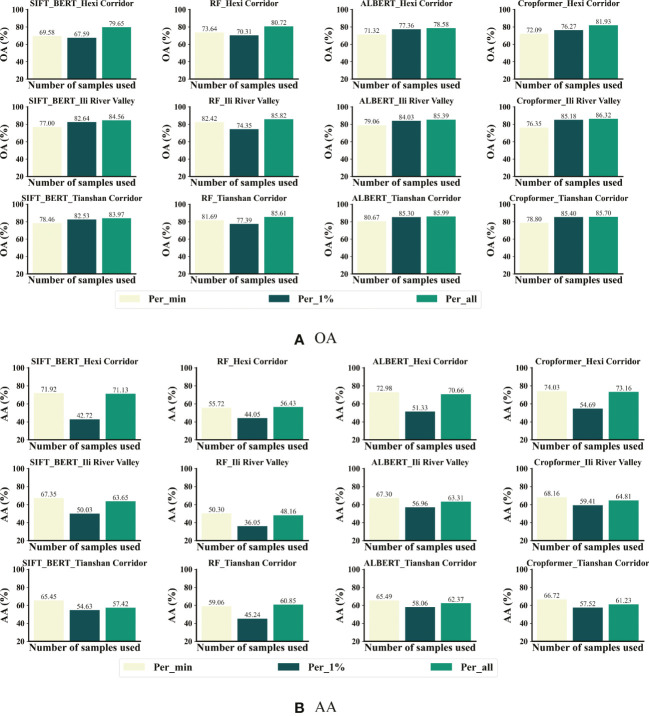
Comparison of results for different proportions of labeled samples in the three study areas. **(A)** OA for few-sample crop classification **(B)** AA for few-sample crop classification.

In the few-sample crop classification experiments, we modeled two schemes to fit the few-sample scenario, i.e., using only 1% labeled samples per category and using only a fixed number of labeled samples per category (the fixed number referred to the number of samples with the least amount of sample size among all categories). We selected RF, SIFT-BERT, and ALBERT, which showed excellent results in previous experiments, as a comparison and conducted experiments in three areas with rich crop types. Each experiment was randomly selected three times to take the average value as the result and the experimental results were shown in **Figure 10**.

**Figure 10A** showed Cropformer achieved OA of 76.27%, 85.18%, and 85.40% in the three study areas when using only 1% labeled samples, which was only 5.66%, 1.14%, and 0.3% less than using all labeled samples. When trained using the 1% sample, RF showed a significant OA decrease of 10.4%, 11.47%, and 8.22% in the three study areas, respectively, while the OA decrease using pre-trained SIFT-BERT, ALBERT, and Cropformer was not significant and only showed a significant OA decrease in study area I. This further demonstrated the effectiveness of pre-training. Moreover, Cropformer also performed optimally when only 1% of the samples were used, but ALBERT achieved comparable performance to Cropformer, while SIFT-BERT showed a significant drop in performance compared to the previous performance. When using the minimum number of samples per class, the accuracy of all methods decreased, which was due to the uniform trend and extremely reduced number of samples in the training set, but the distribution in the test set was still extremely uneven, which leaded to a significant decrease in accuracy.

**Figure 10B** showed that the AA of Cropformer reached 74.03%,68.16% and 66.72% when using the minimum number of samples per class, which was 0.87%, 3.35% and 5.49% higher than the AA using the full sample fine-tuning. This further supported the previous conclusion that when the sample distribution was uneven, the category with the larger sample size dominates. Whether using the minimum number of samples per class or 1% samples per class, classification results of Cropformer still outperformed other methods, while SIFT-BERT and ALBERT were second. As with the previous classification results evaluated in terms of AA, the AA of RF was the lowest among all methods, which showed that RF was not applicable to samples with unbalanced distribution. Overall, Cropformer achieved competitive classification results in few-sample context classification, both in unbalanced and balanced samples.

### 5.4 Spatial transfer of classification model

If the target region was not rich in labeled samples for training, transferring the model trained in the source region to the target region could reduce the problem of sparse labeled samples. We set up two types of transfer across regions, one was between regions with similar geographic and climatic conditions(TL1), and the other was to use regions with rich labeled samples to transfer to regions with fewer labeled samples(TL2). Study area II was the area with abundant labeling samples, and study area IV and study area V had similar geographical and climatic conditions. Since study area II and study area V were not in the same geographical area, we selected pre-training data from the two areas for pre-training. Where Pre_W and Pre_E represented the pre-training sets for the Northwest and Northeast regions of China, respectively. The crops were common to all three areas: soybean, spring maize, middle rice, and others. The results of the experiment are shown in **Table 4**.

**Table 4:** Comparing the results of different transfer strategies.

Pre-training Area	Methods	Study AreaIV→ Study AreaV		Study AreaII→ Study AreaV	
		AA(%)	OA(%)	AA(%)	OA(%)
No_Pre	RF	37.93	25.21	41.52	47.68
	Performer	33.65	30.54	43.62	44.38
	Cropformer	25.96	62.13	27.68	62.98
Pre_W	ALBERT	–	–	22.65	43.67
	BERT	–	–	23.34	32.77
	Cropformer	–	–	25.65	61.70
Pre_E	ALBERT	**35.93**	52.34	29.32	63.40
	BERT	28.36	62.98	44.78	62.55
	Cropformer	29.65	**63.77**	**45.46**	**64.26**

Bolded indicates best results.

**Table 4** showed that Cropformer achieved OA of 62.13% in TL1 when no pre-training was used, which was a 36.92% and 31.59% improvement compared to RF and Performer. In TL2 the OA reached 62.98%, a 15.3% and 18.6% improvement compared to RF and Performer. However, the AA of Cropformer was not outstanding among the two transfer methods. When using Pre_W as pre-training, the OA of Cropformer reached 61.70%, which was still more than 20% improvement compared to ALBERT and SIFT-BERT. When using Pre_E as pre-training, Cropformer achieved OA of 63.77% in TL1 and OA of 64.26% in TL2. Compared to the first two pre-training methods, the OA improvement of Cropformer was much less using the third pre-training method, which indicated that the region of pre-trained data need to be consistent with the region of fine-tuned data. However, Cropformer can overcome the scenario of regional inconsistency, reflecting Cropformer’s ability to transfer across regions. Cropformer’s ability to capture key information about the crop and understand crop growth patterns from the entire reproductive period of the crop allowed Cropformer to identify crops in different regions faster and better, which was important reason for Cropformer’s good spatial transfer capability.

In the no-pre-training scenario, the average OA/AA of the three methods in TL2 reached 51.68%/37.61%, which was an improvement of 12.48%/5.1%, respectively, compared to that in TL1 (39.2%/32.51%). When pre-training with Pre_E, the average OA/AA of the three methods in TL2 reached 63.40%/39.85%, which was an improvement of 3.7%/8.54% compared to TL1 (59.70%/31.31%), respectively. The results indicated that TL2 outperformed TL1, and thus it can be assumed that models trained in areas with rich label samples outperformed those trained in areas with similar geographical location and climate.

### 5.5 Processing efficiency

We compared the processing speed of Cropformer with the other three methods in the Ili River Valley and ensured that all experimental settings were consistent, and the experimental results were shown in **Table 5**.

**Table 5:** Comparison of processing speed between cropformer and other methods(s).

Methods	RF	Res-18	Performer	ALBERT	SIFT-BERT	Cropformer
Data pre-processing	523.93	–	523.92	–	–	–
Pre-training epoch	–	–	–	230.36	275.25	315.88
Training epoch	271.65	1165.24	470.69	196.85	242.84	250.63
All time consuming	795.58	1165.24	994.61	427.21	518.09	566.51

**Table 5** showed that the total time consumption of Cropformer (566.51s) was higher than that of BERT (518.09s), ALBERT (427.21s), and Performer (994.61s), but lower than that of RF (795.58s) and Res-18 (1165.24s). The main time consumption of RF and Performer consumption lied in data preprocessing (523.93s), which accounted for 65.86% and 52.68% of all time consumed, which severely limited the efficiency of RF and Performer. Cropformer was more time consuming than SIFT-BERT and ALBERT because we added a convolutional part to the network structure. ALBERT had the advantage of having a very small number of parameters, which was an important reason why its efficiency is the best among all methods.

### 5.6 Comparison of the crop maps

**Figure 11:**
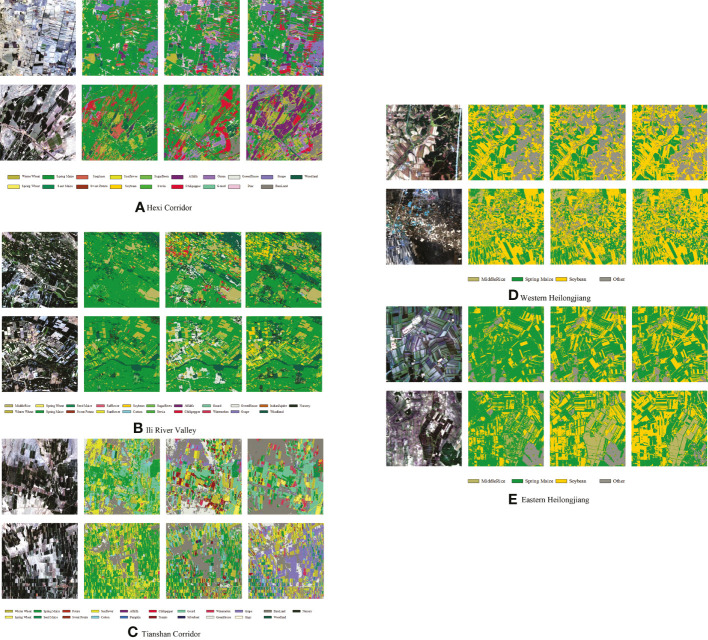
Map of crop distribution in selected areas of the five study areas. For each study area, from left to right, remote sensing images, RF mapping, SIFT- BERT mapping, and Cropformer mapping.

We selected two 10 km ×10 km areas in each of the five study areas for full-season mapping using Cropformer, and compared the results with those of RF, and SIFT-BERT, as shown in **Figure 11**. Results of crop mapping by other methods can be viewed in the **Supplementary Material**.

**Figure 11** showed the mapping differences between the three methods were more pronounced in the first three study areas with rich crop types, while the three methods were similar in the last two study areas with similar cropping structures. The most significant differences in the maps among the five methods were found in the Tianshan Corridor, for example, in the second 10 km × 10 km area, Cropformer identified most of the crops as grapes, while RF identified most of the crops as spring maize and sunflower, and SIFT-BERT identified them as seeded mazie and cotton. Based on the field survey in Tianshan Corridor, an important grape production base in China, the prediction of Cropformer was more accurate. In the area with complex planting structures, Cropformer still showed good mapping performances. The parcel distribution of Cropformer mapping is very close to plot distribution of the original remote sensing image, and the mapping results of other methods had a serious pepper effect and look more fragmented. In the latter two study areas where the plot size was small and clustered distributed, Cropformer still overcame the pepper effect and there were few cases of fragmented plots. Overall Cropformer had good mapping results and provides a possible solution for large-scale remote sensing mapping.

## 6 Discussion

In this paper, we proposed a new crop classification method that can be applied to multi-scenario crop classification, and its generality and validity were demonstrated in five study areas with complex crop growing structures.

The success of Cropformer lied in the ability to focus on both global information about crop growth and to capture key features of crop growth to achieve a more comprehensive feature representation from the perspective of feature complementarity. For the features extracted by the model, neither local nor global features can fully characterize the crop growth pattern (Gulati et al., 2020). Therefore, better results were obtained using more comprehensive and integrated features. The role of pre-training was to help the model better understand the crop growth pattern, and when pre-training was introduced in the classification method, the classification accuracy was significantly improved, which was consistent with the findings in the literature (Yuan and Lin, 2021; Yuan et al., 2022). The implication behind pre-training was to make the model learn the contextual relationships of the time series by forcing the model to learn them through self-supervised training when inputting unlabeled time series, thus summarizing the time series patterns of the crop. These laws were used as prior knowledge to the fine-tuning phase, which both reduced the need for labeled samples and sped up the convergence of the model (Li et al., 2020). The introduction of position encoding reduced the requirement of time series as input. Using time and spectra as inputs to the model ensured the correct correspondence between image acquisition time and crop spectra in the time series, and also enriched the inputs to the model.

The unlabeled remote sensing data were very easy to obtain, and only some areas were randomly selected for pre-training in this paper, without considering the influence of the land cover degree of the area where the unlabeled remote sensing data were located on the results. Therefore, it was the focus of future work to fully exploit the potential of unlabeled remote sensing data in crop classification, including the effects of different types of unlabeled remote sensing data and remote sensing data of different time series length on the classification results. Data augmentation was an important tool for enriching sample types and avoiding model overfitting (Vulli et al., 2022), which would also be applied to crop classification in future work. In addition, the experimental results across regions were not satisfactory, and how to solve the effective migration of the model in large scale crop classification was also worthy of attention.

## 7 Conclusion

To build deep learning models that can be applied to multi-scene crop classification, we created a two-step classification system and proposed a new deep learning architecture, Cropformer. Cropformer can adapt irregular time series as input and can accumulate crop growth information in the pre-training phase, which enabled it to achieve the best performance in multiple crop classification scenarios. In full-season crop classification experiments, the average OA/AA of Cropformer with Transformer and convolution (83.50%/70.19%) outperformed traditional classification methods RF (80.73%/58.36%), the single convolution structure Res-18 (78.88%/43.38%) and the single Transformer structure of SIFT-BERT (81.70%/69.50%), indicating that Cropformer had the ability to extract more comprehensive features by using both the Convolution structure to extract local features and the Transformer to capture global information. The results of in-season crop classification experiments showed that Cropformer can obtain classification results comparable to those of crop maturity (end of September) at mid-to-late crop growth (end of July), reflecting Cropformer’s ability of early identification, taking advantage of the ability to use irregular time series directly, and thus extracting usable features from limited images. In the classification of crops with few samples, the average OA of only 1% of the samples used by Cropformer for each class reached 82.28%, which was 2.37% lower than that of all the samples (84.65%). This showed that after pre-training, Cropformer had accumulated a lot of prior knowledge, which can effectively reduce the demand for standard label samples, so that it can obtain high-precision classification results with few labeled samples. The results of spatial transfer experiments showed that Cropformer can overcome the problem of inconsistency between the regions of the pre-training data and the fine-tuning data, indicating that Cropformer had the ability of spatial generalization. Crop mapping results showed that Cropformer can obtain mapping results consistent with field samples, which benefited from Cropformer’s strong learning ability and can learn generalized features. All experiments showed that Cropformer adapts to multi-scenario crop classification and had great potential in large-scale crop classification.

## Data availability statement

The raw data supporting the conclusions of this article will be made available by the authors, without undue reservation.

## Author contributions

Th HW, WC, YY, ZY, YZ, SL, ZL, and XZ conducted the field experiment. HW conducted the image analysis. All authors contributed to the article and approved the submitted version.
