# Supplementary material for: Cropformer: A new generalized deep learning classification approach for multi-scenario crop classification
Source: Front Plant Sci. 2023 Mar 2;14:1130659. doi: 10.3389/fpls.2023.1130659 (PMC10017990; doi:10.3389/fpls.2023.1130659)
Supplement: Supplementary file 1 [file DataSheet_1.docx]

Supplementary Material

Cropformer: A New Generalized Deep Learning Classification Approach for Multi-Scenario Crop Classification

Hengbin Wang^1^, Wanqiu Chang^1^, Yu Yao^1^, Zhiying Yao^1^, Yuanuan Zhao^1,2*^, Shaoming Li^1,2^, Zhe Liu^1,2^, Xiaodong Zhang^1,2^

^1^College of Land Science and Technology, China Agricultural University, Beijing, China

^2^Key Laboratory of Remote Sensing for Agri-Hazards, Ministry of Agriculture and Rural Affairs, Beijing, China

*** Correspondence:**Yuanyuan Zhao
zhaoyuanyuan@cau.edu.cn

# Supplementary Figures and Tables

## Supplementary Figures


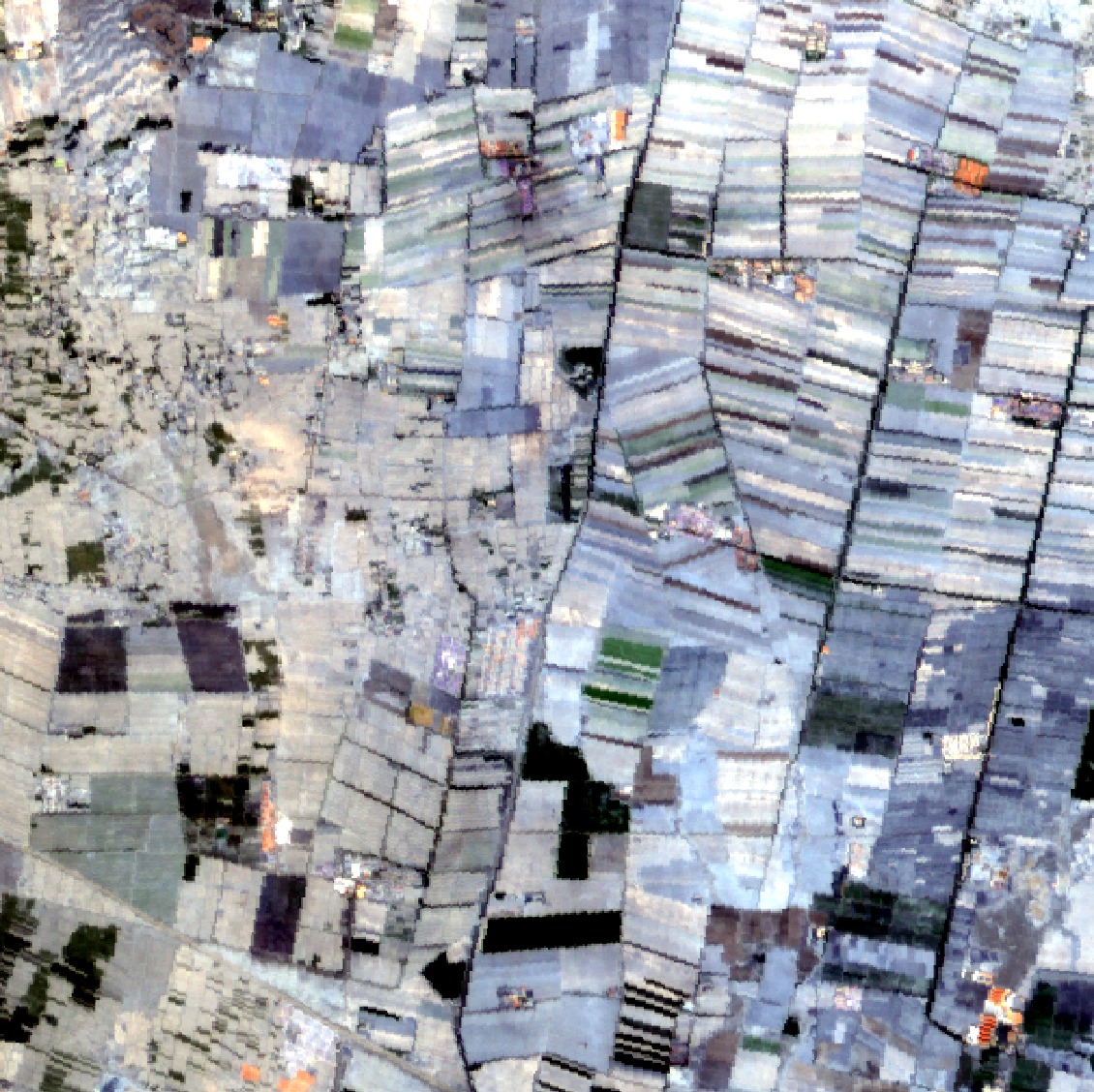

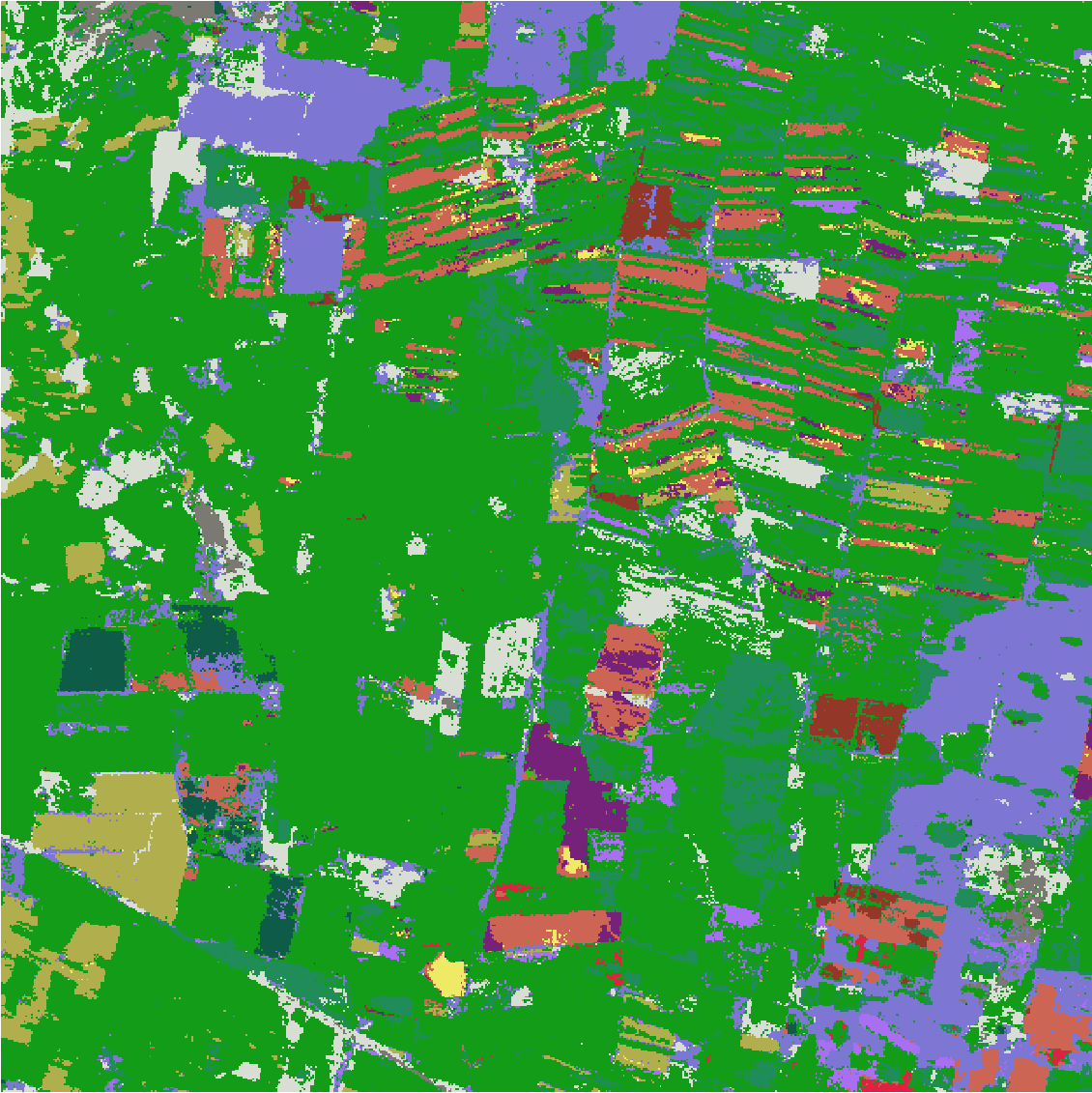

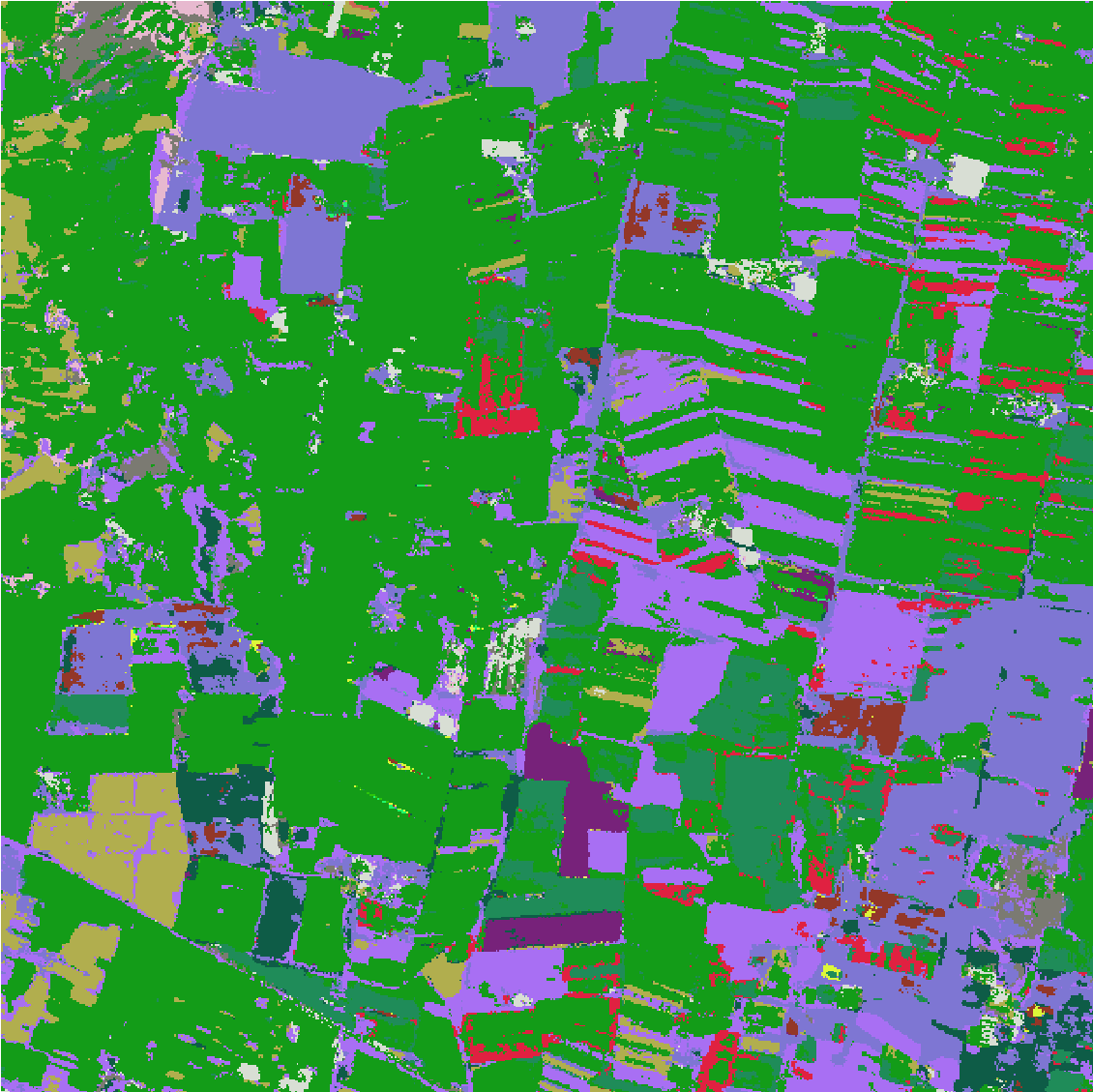


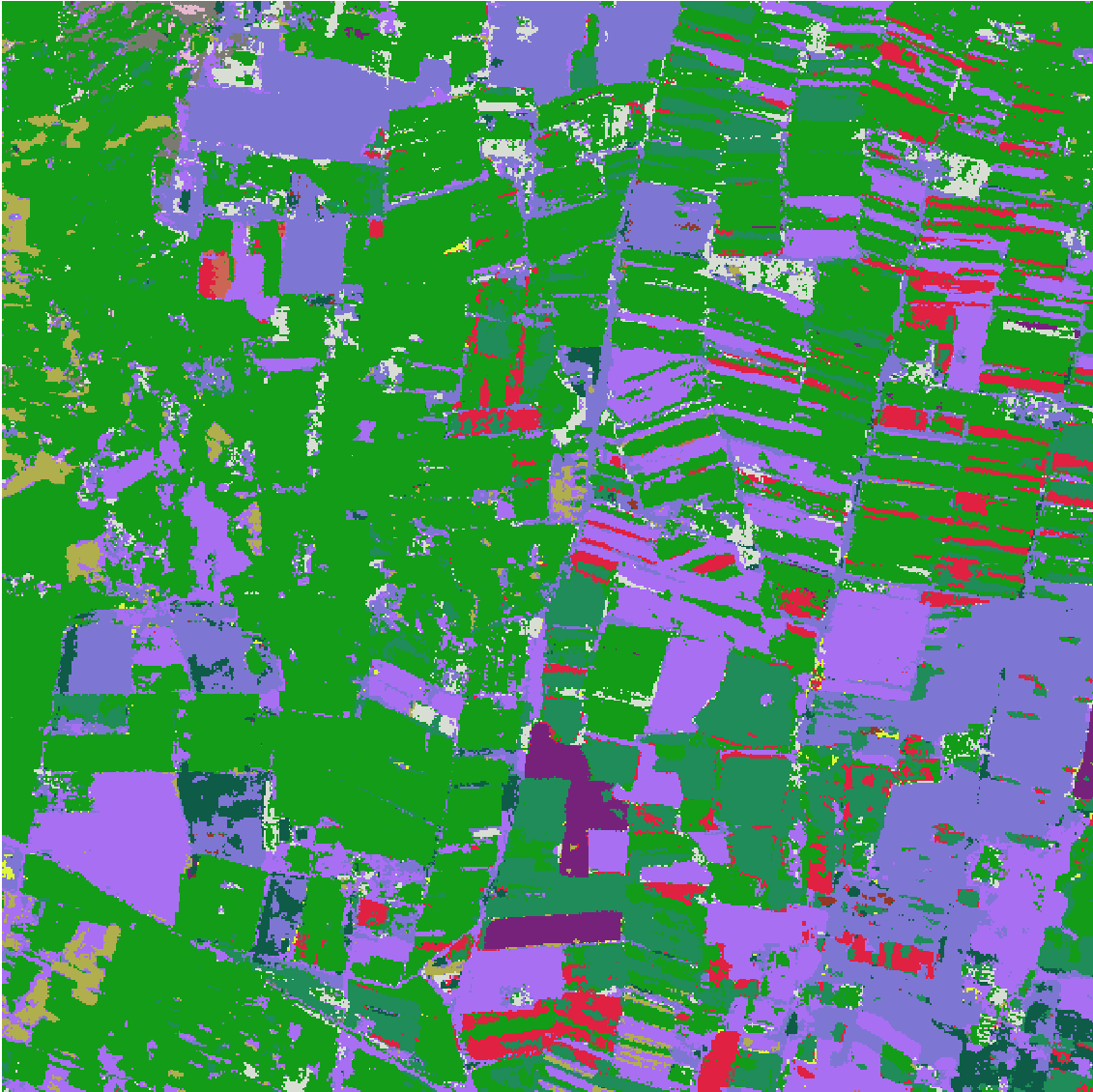

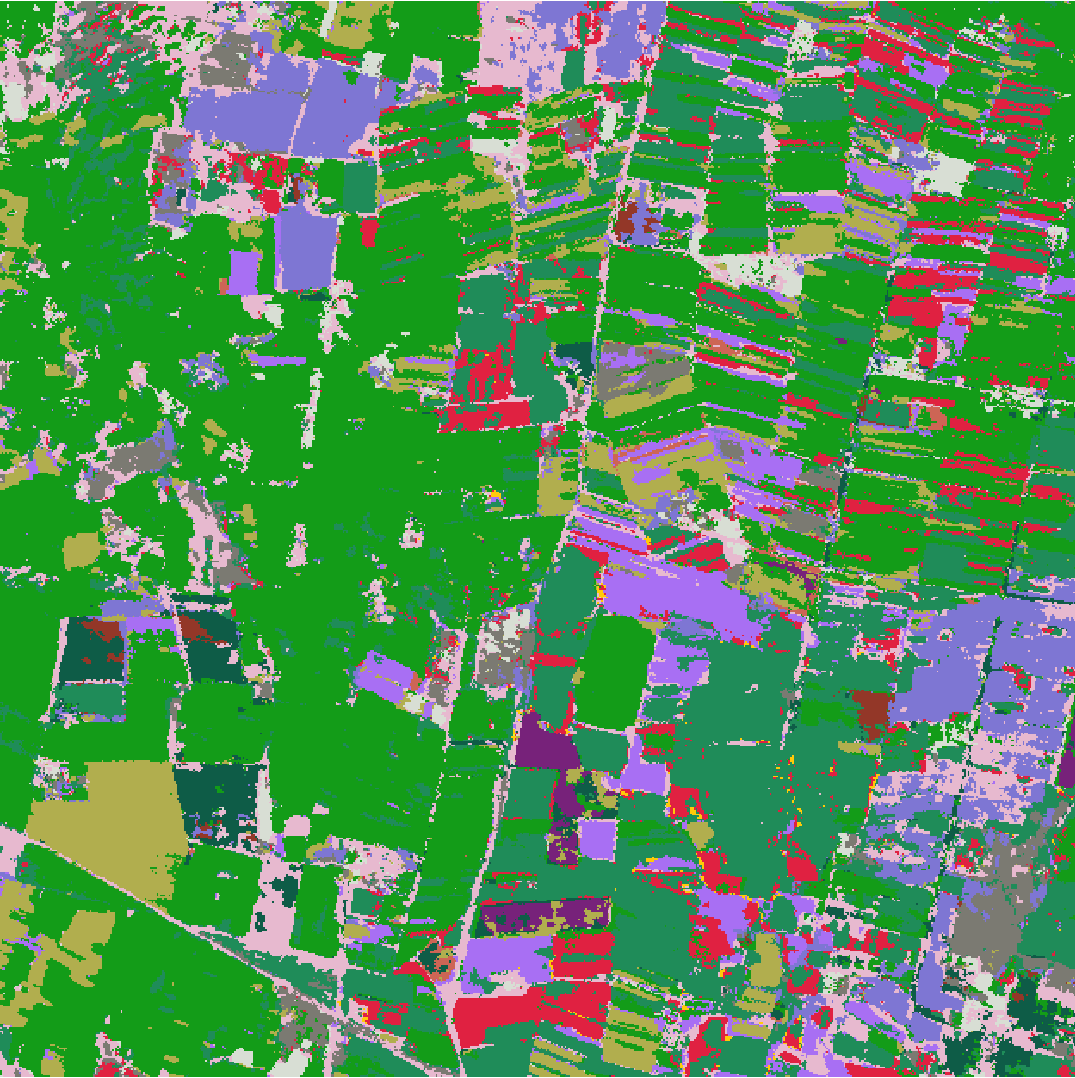

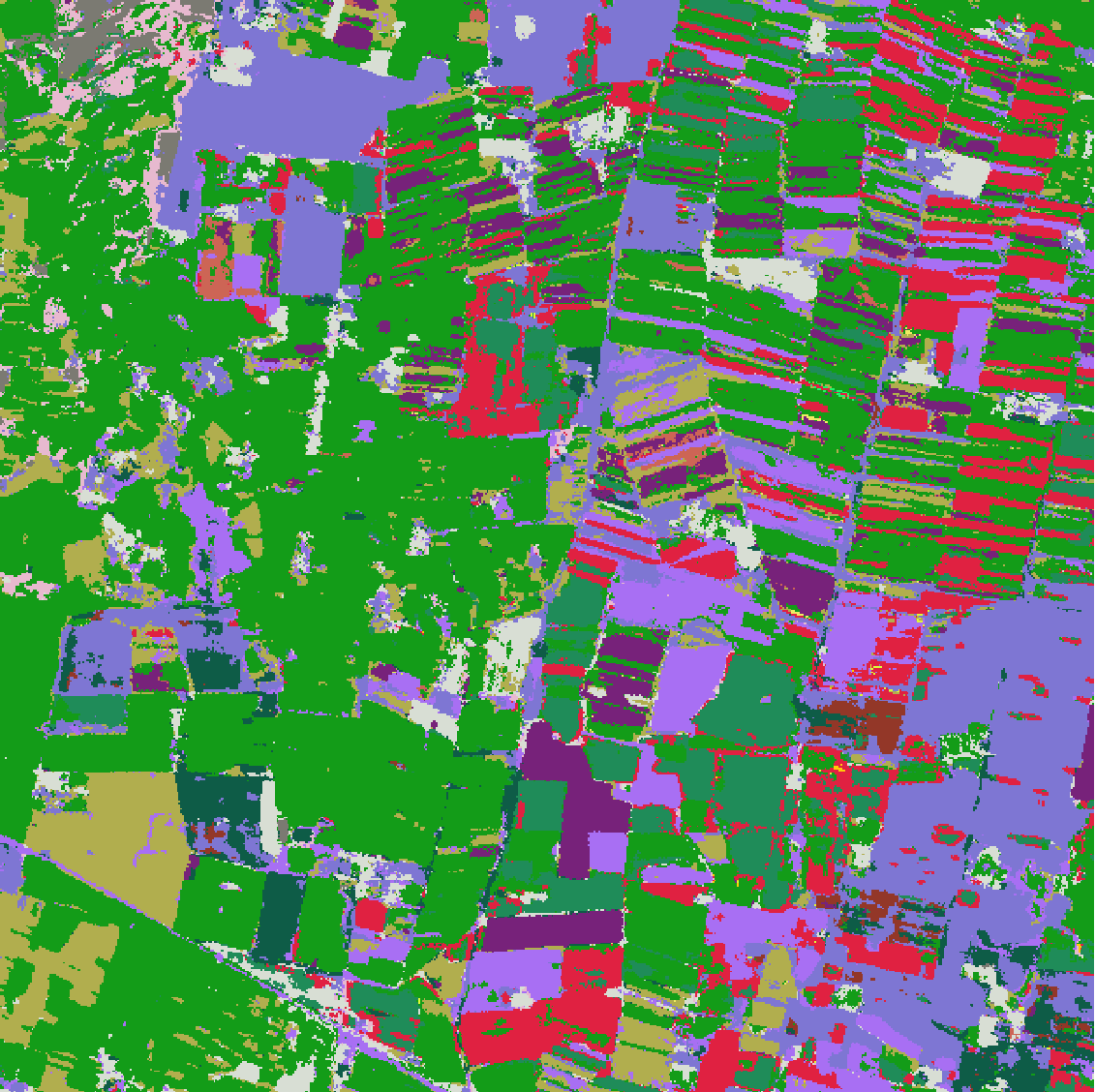


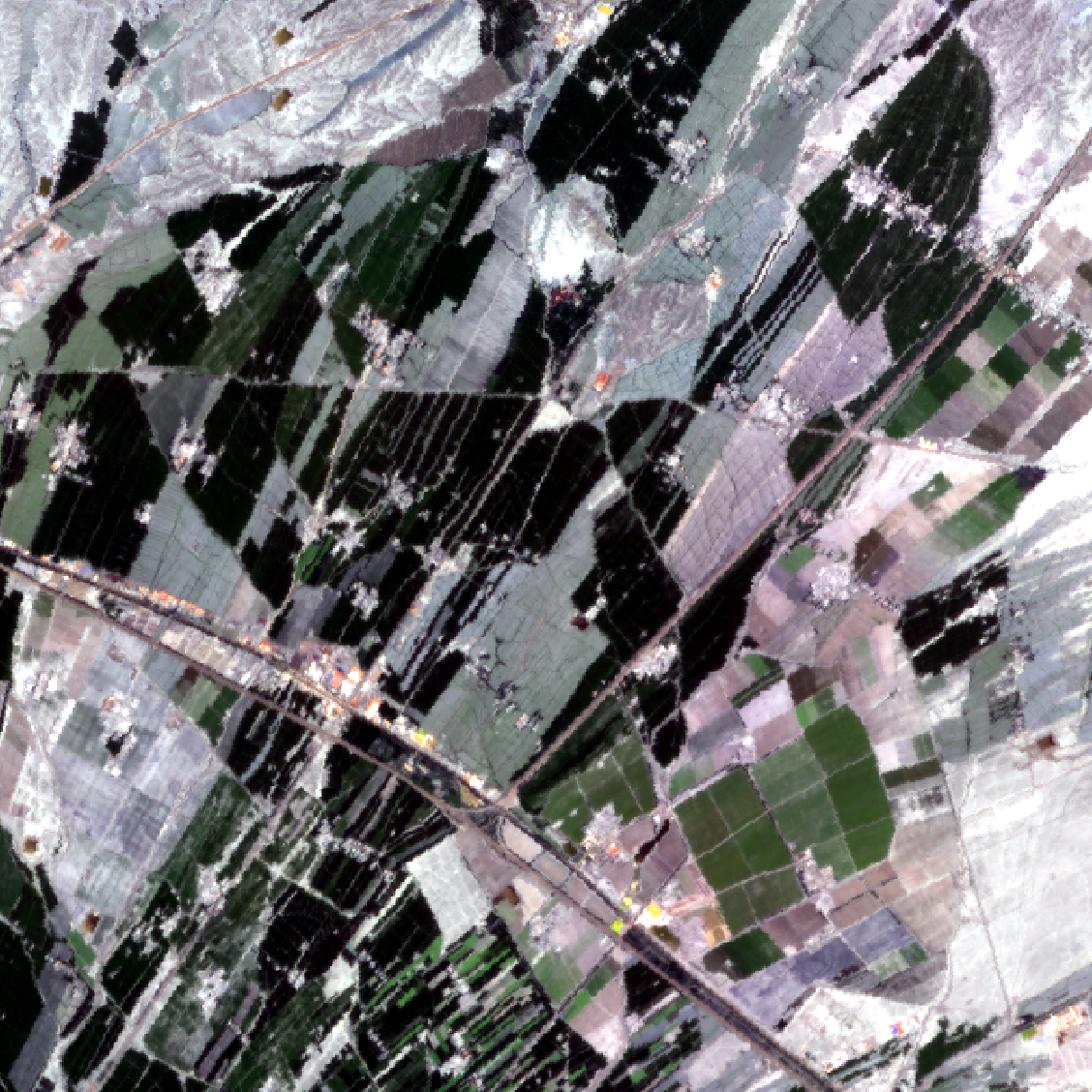

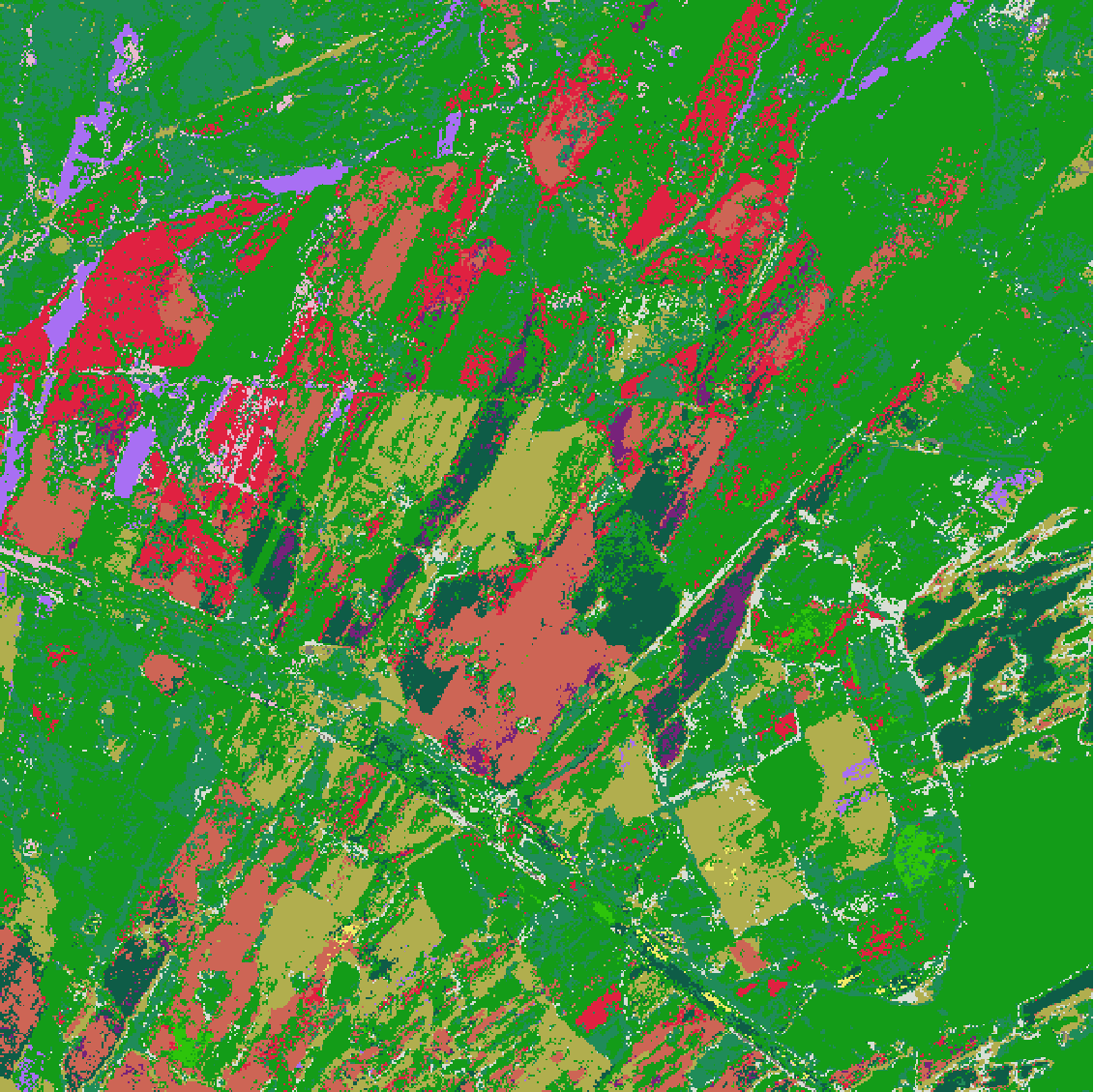

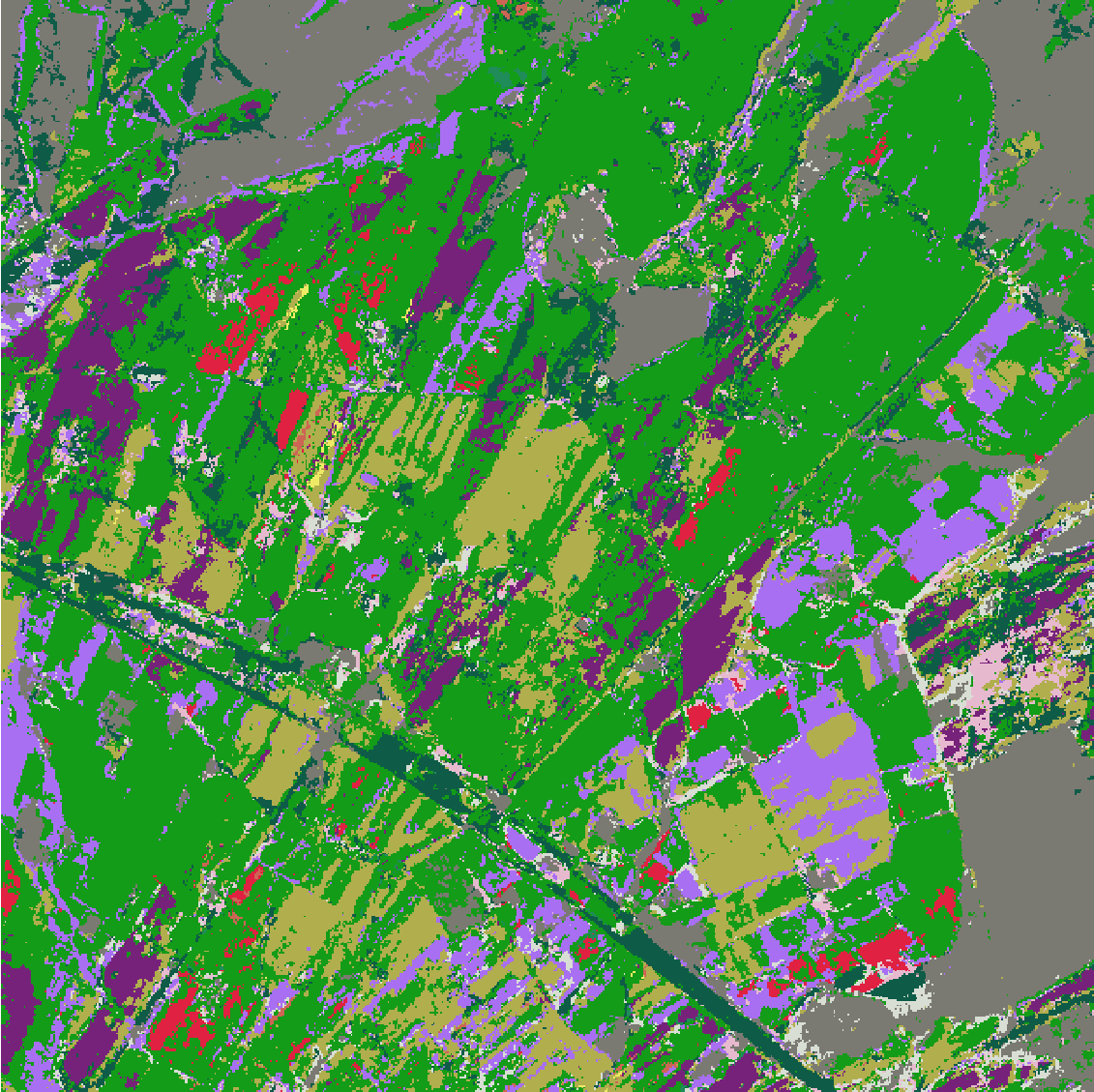


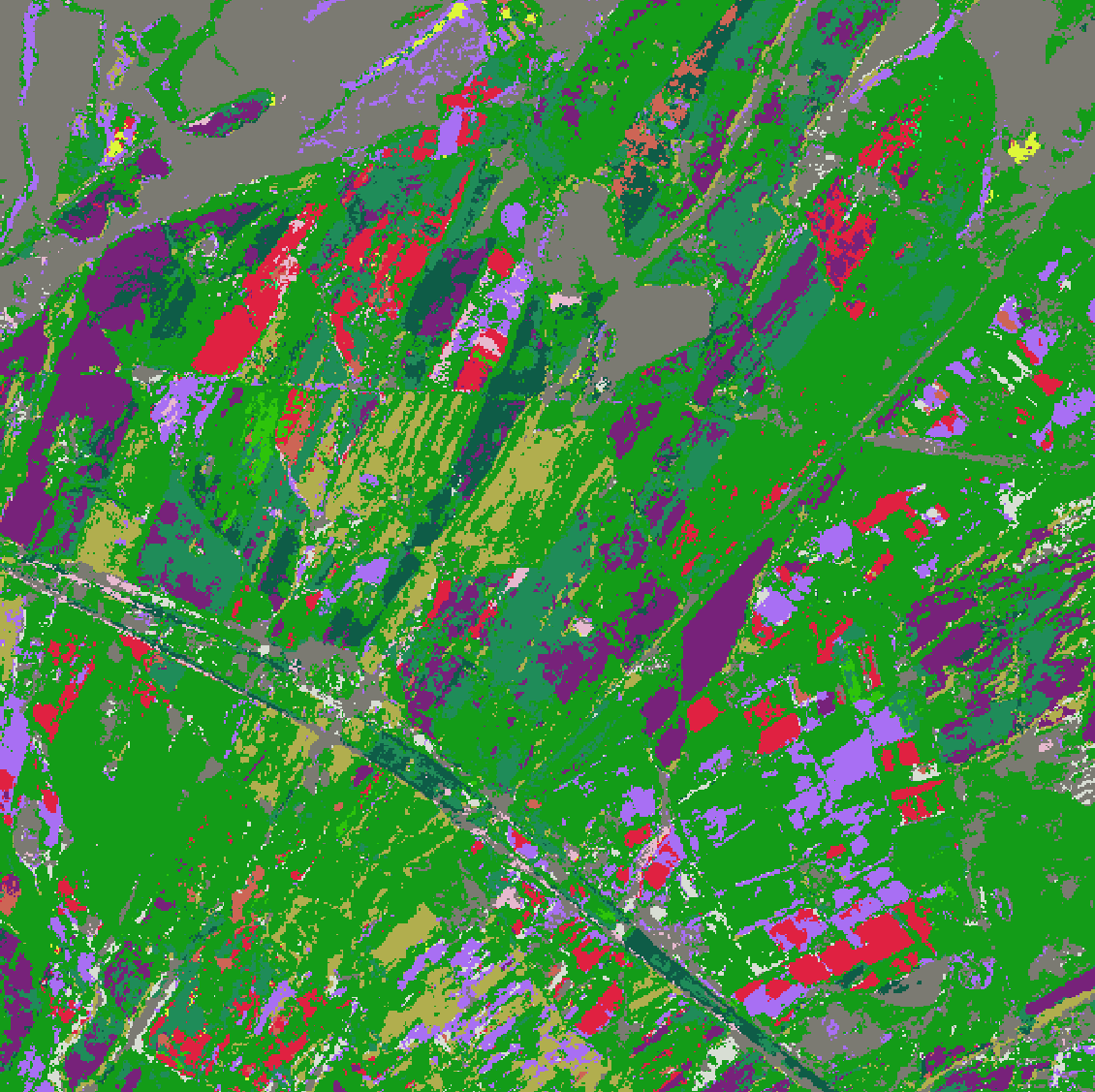

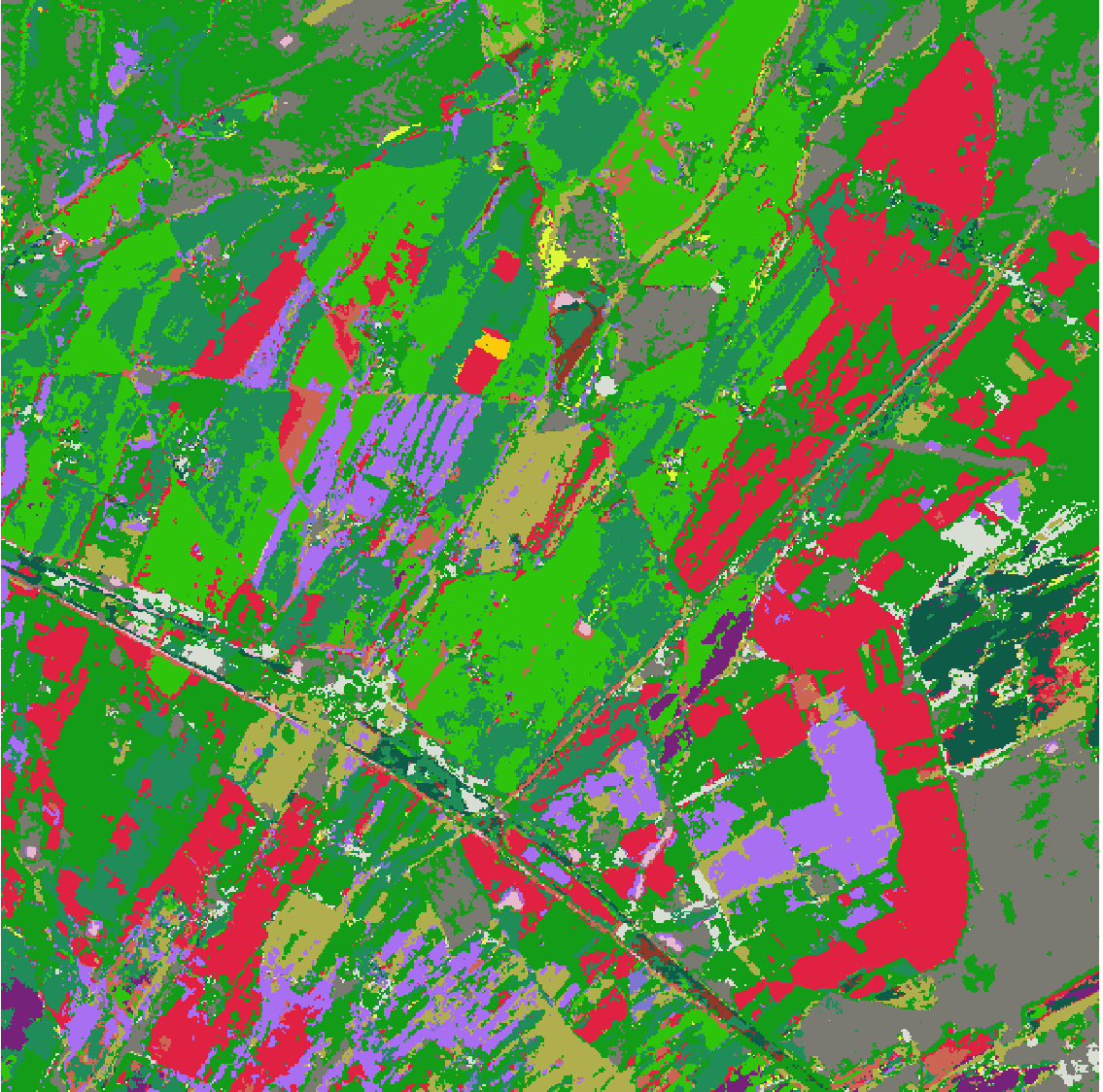

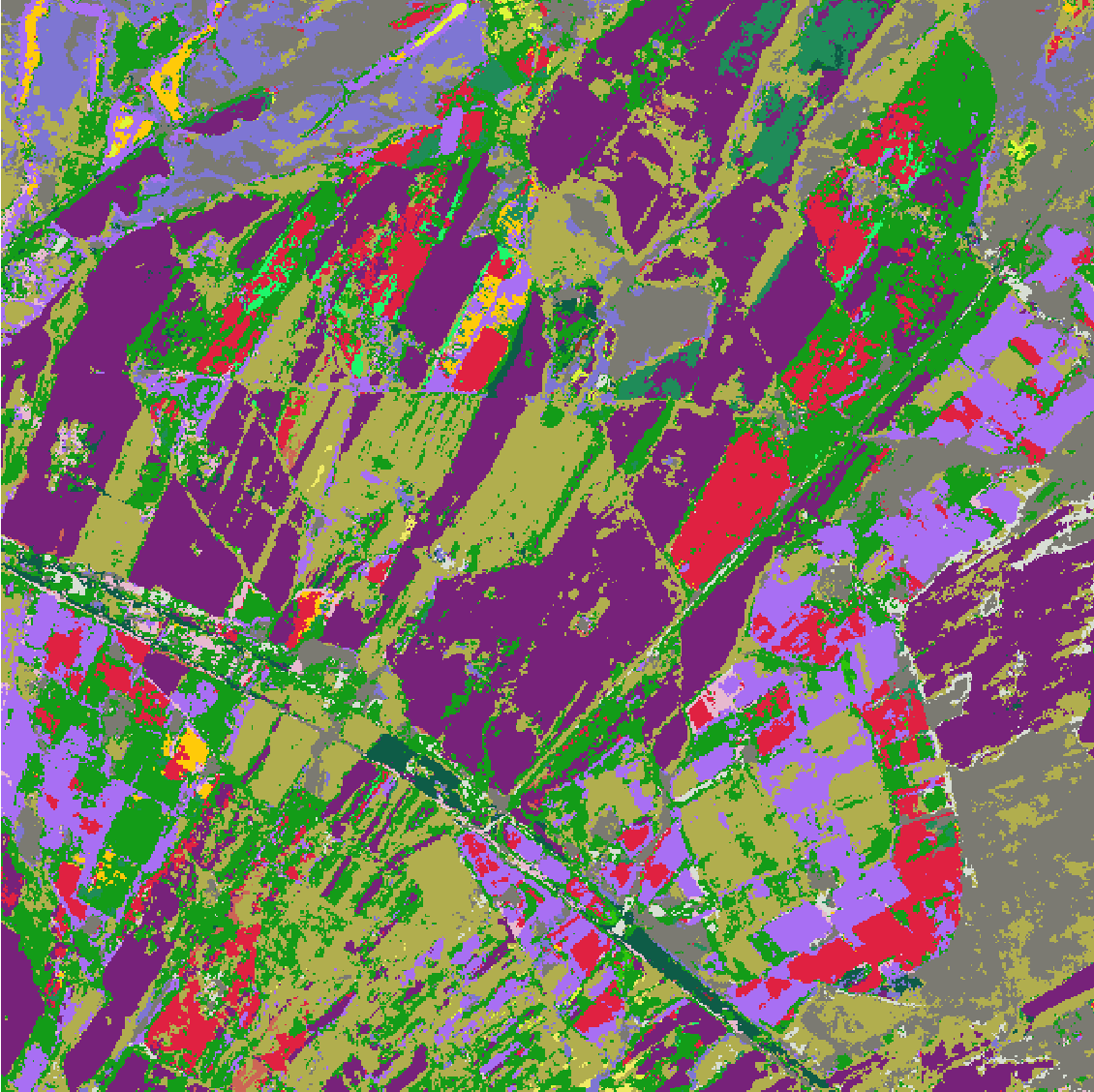


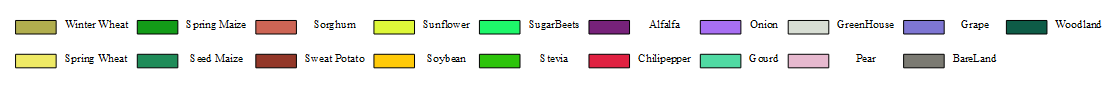


1. Hexi Corridor


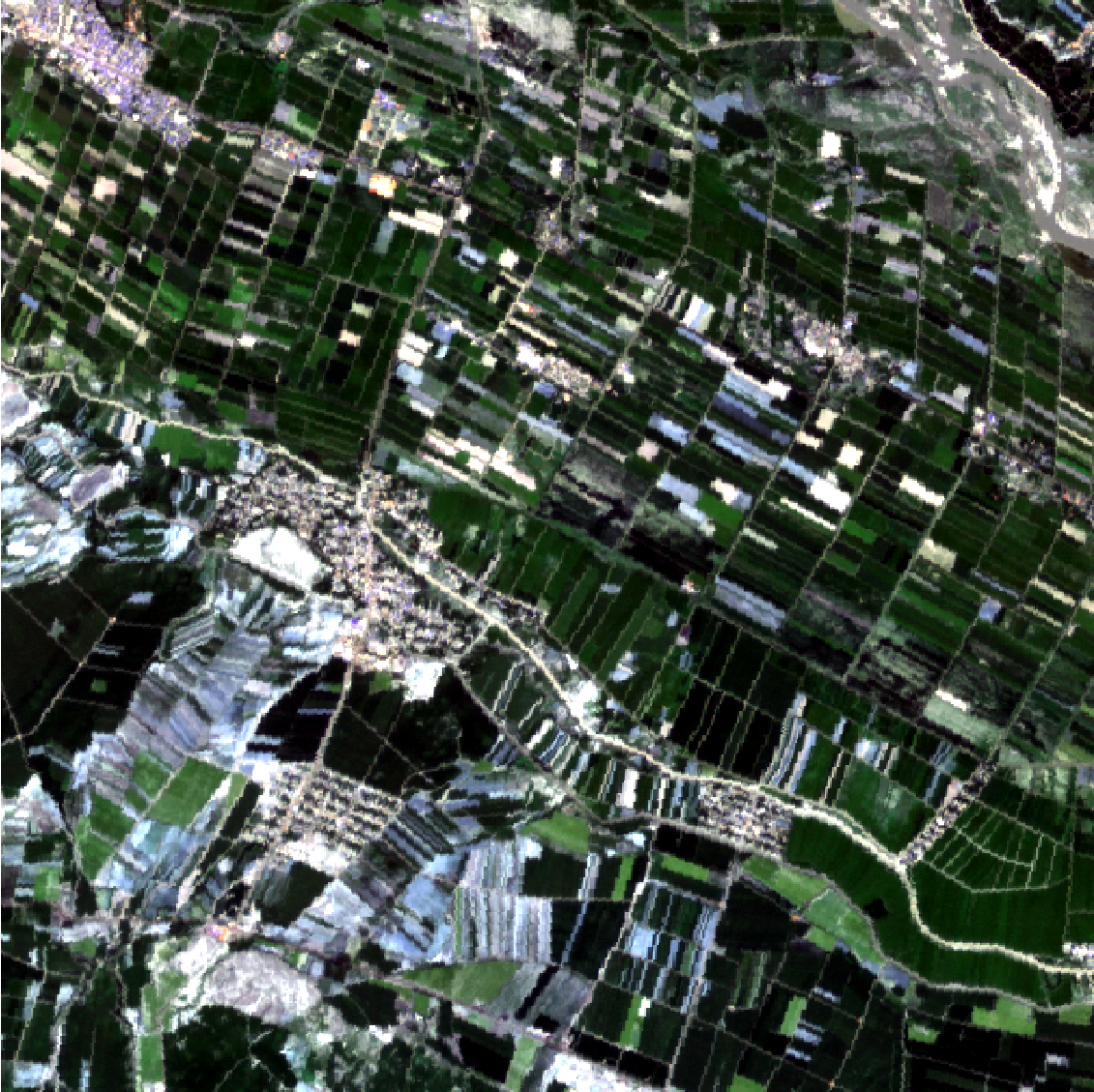

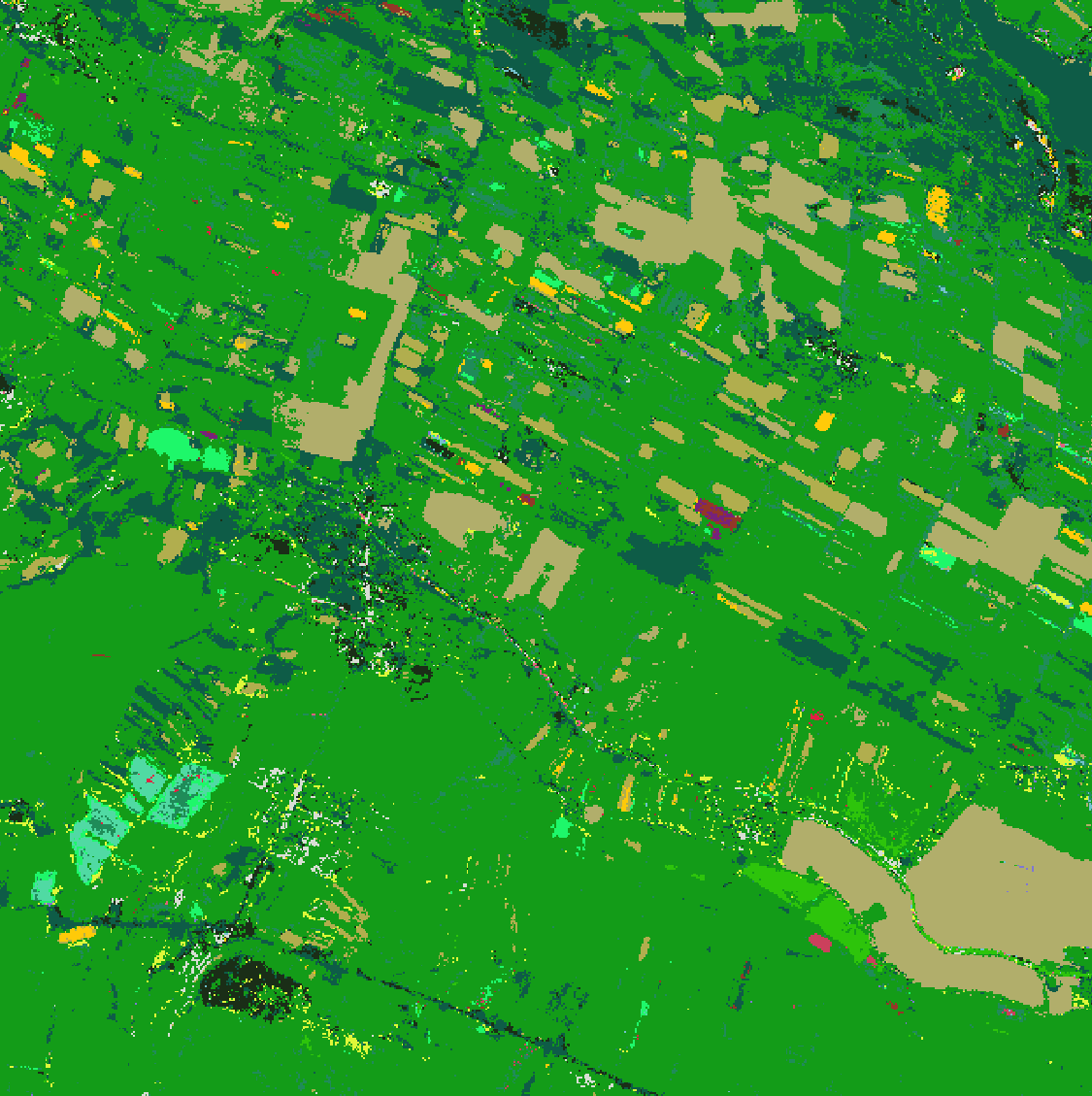

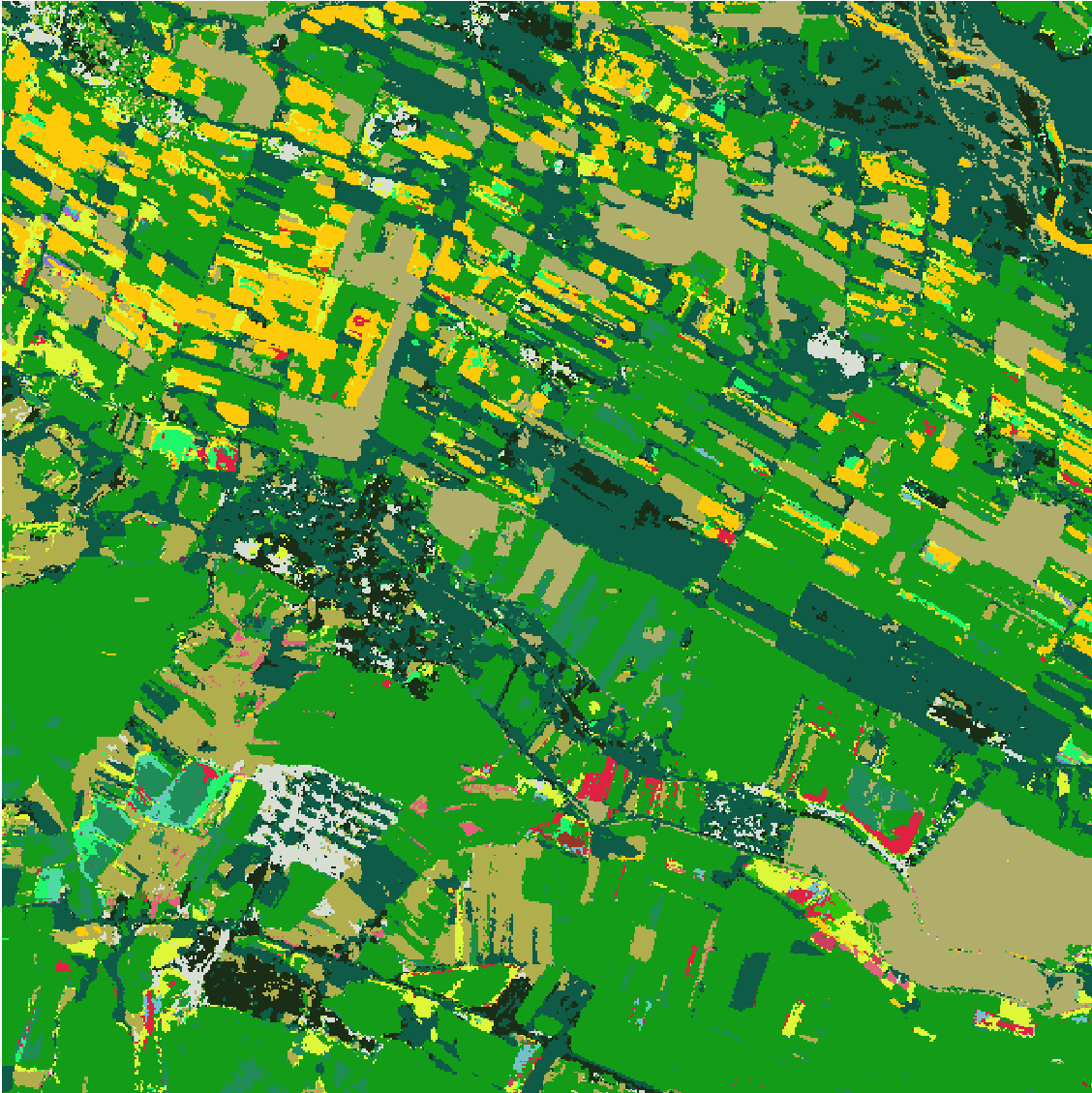


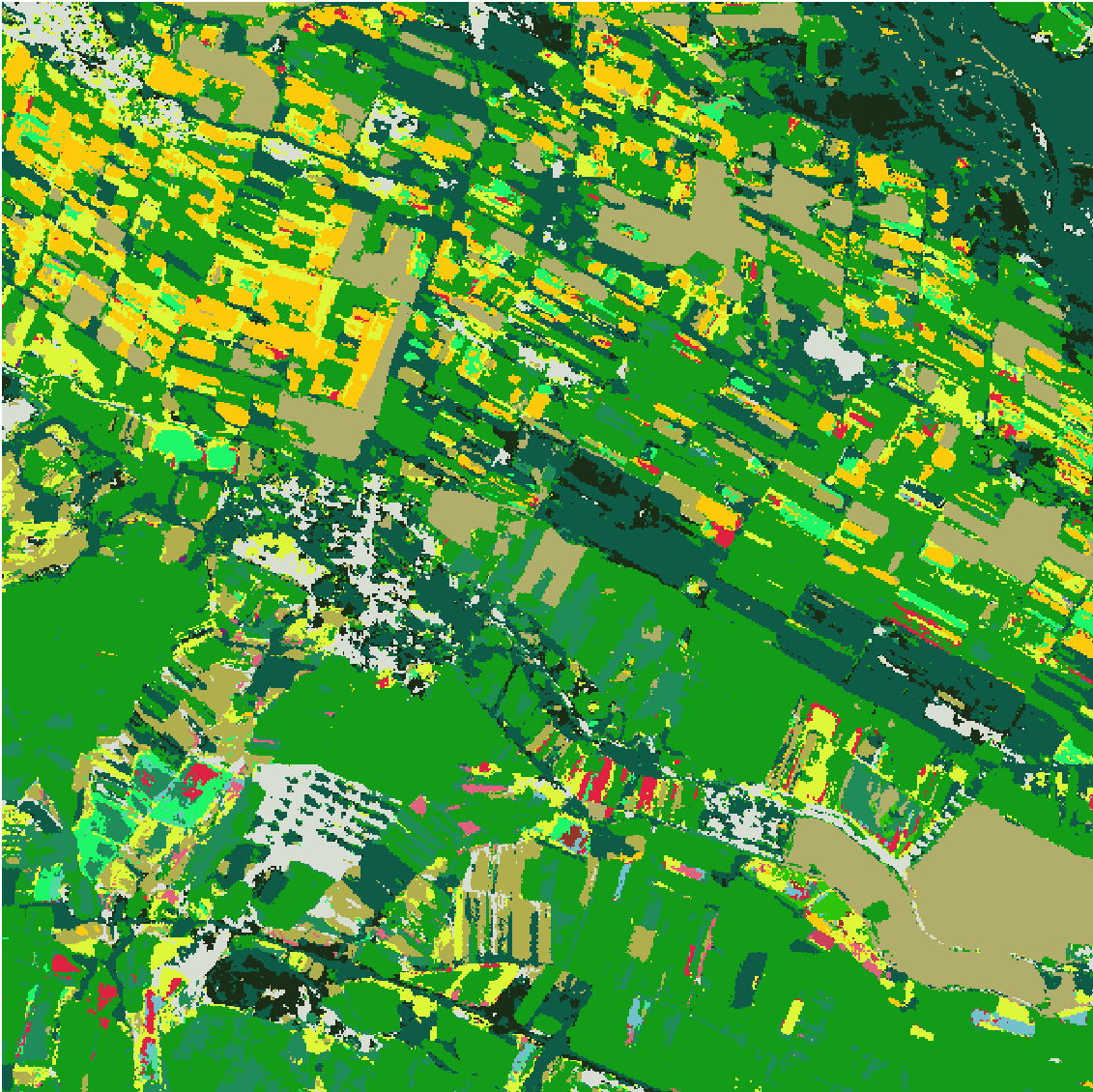

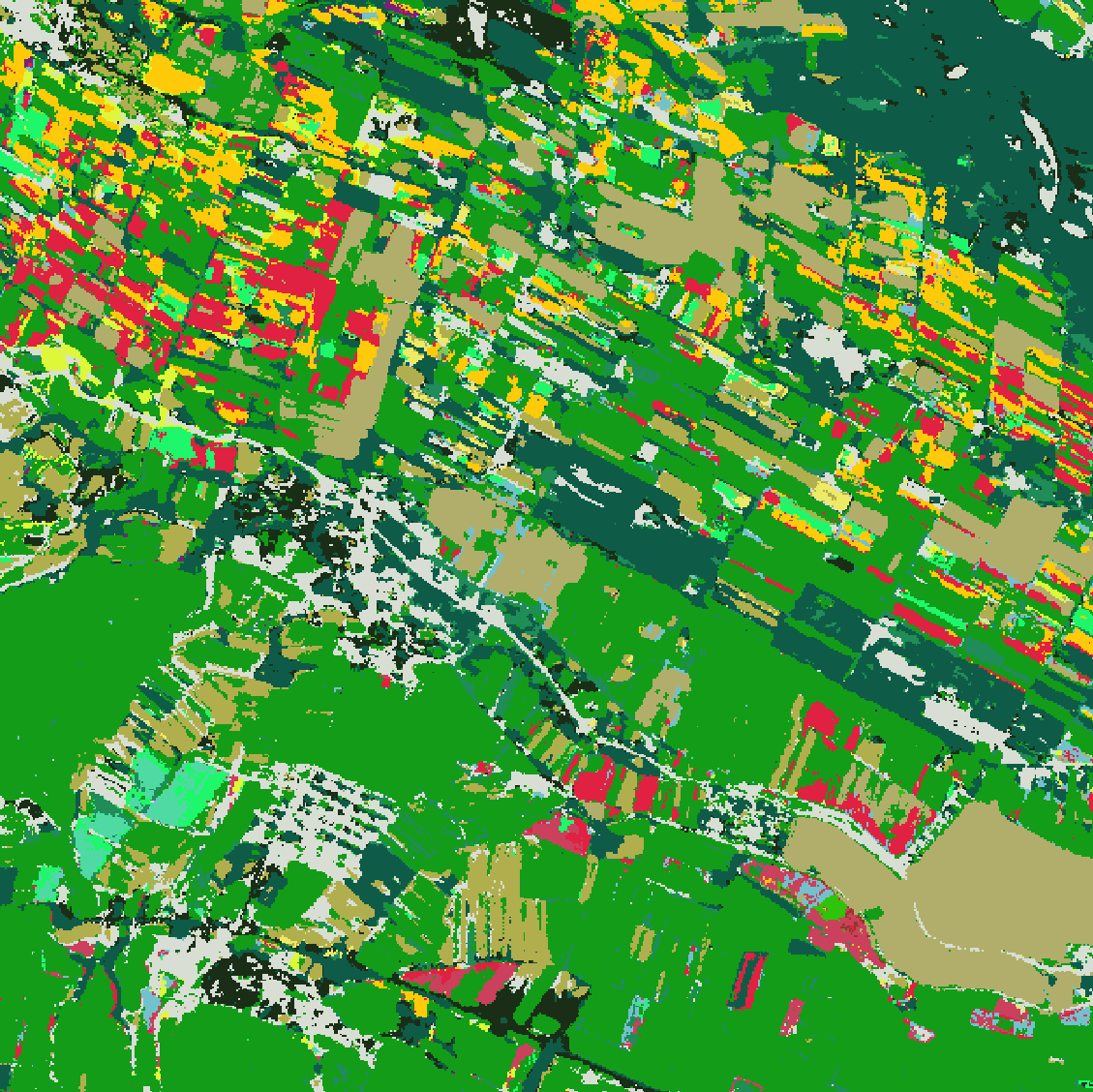

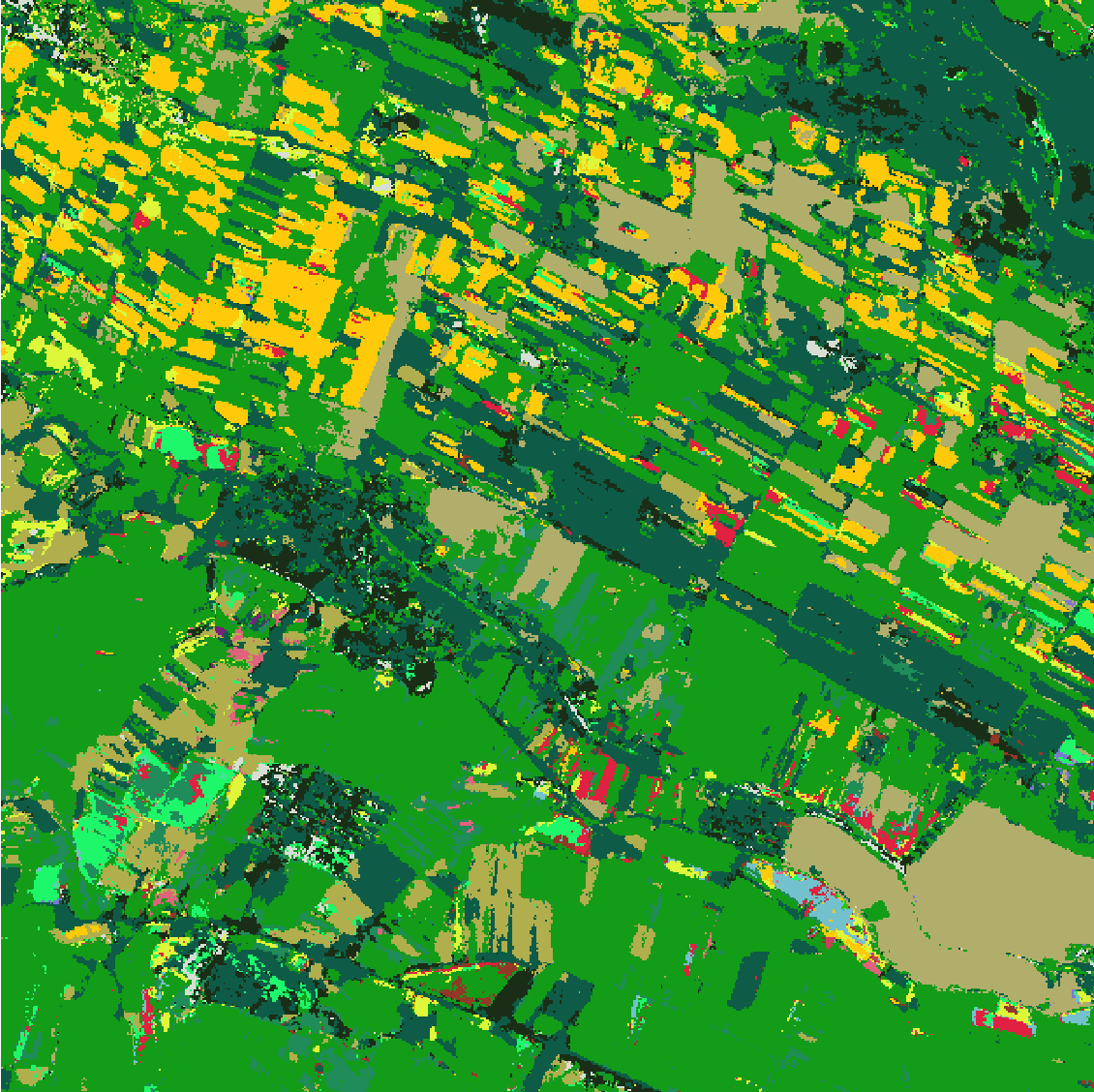


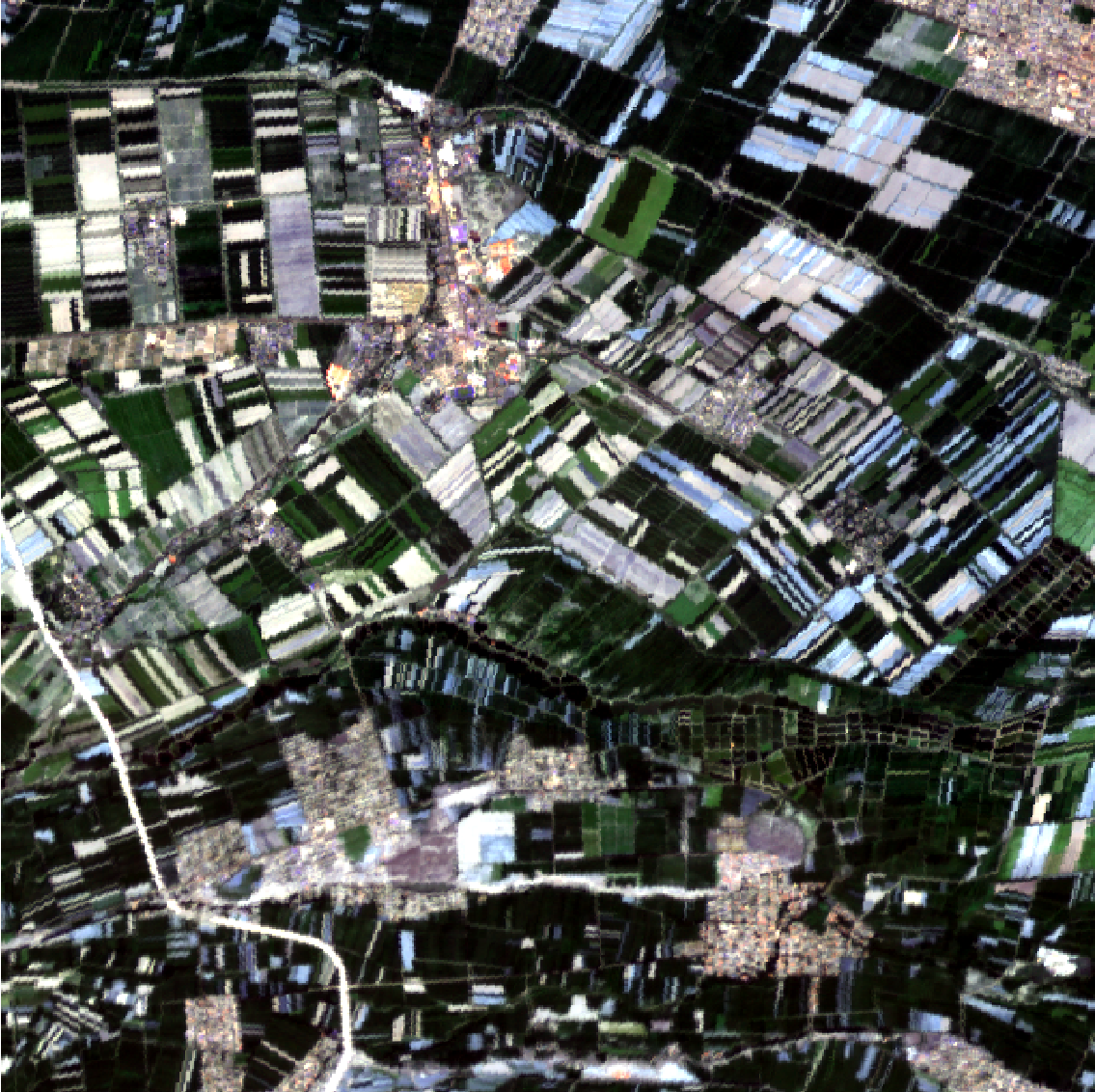

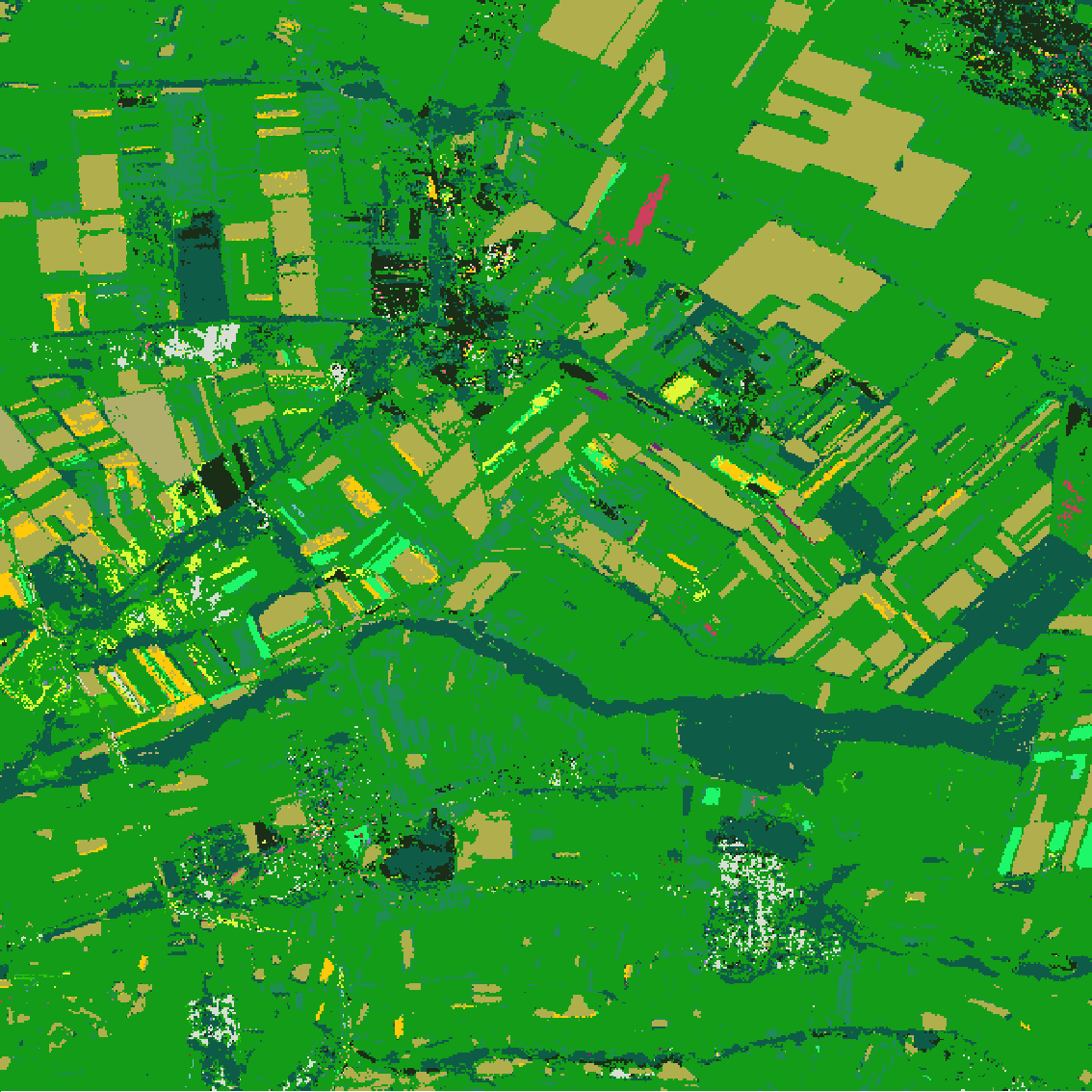

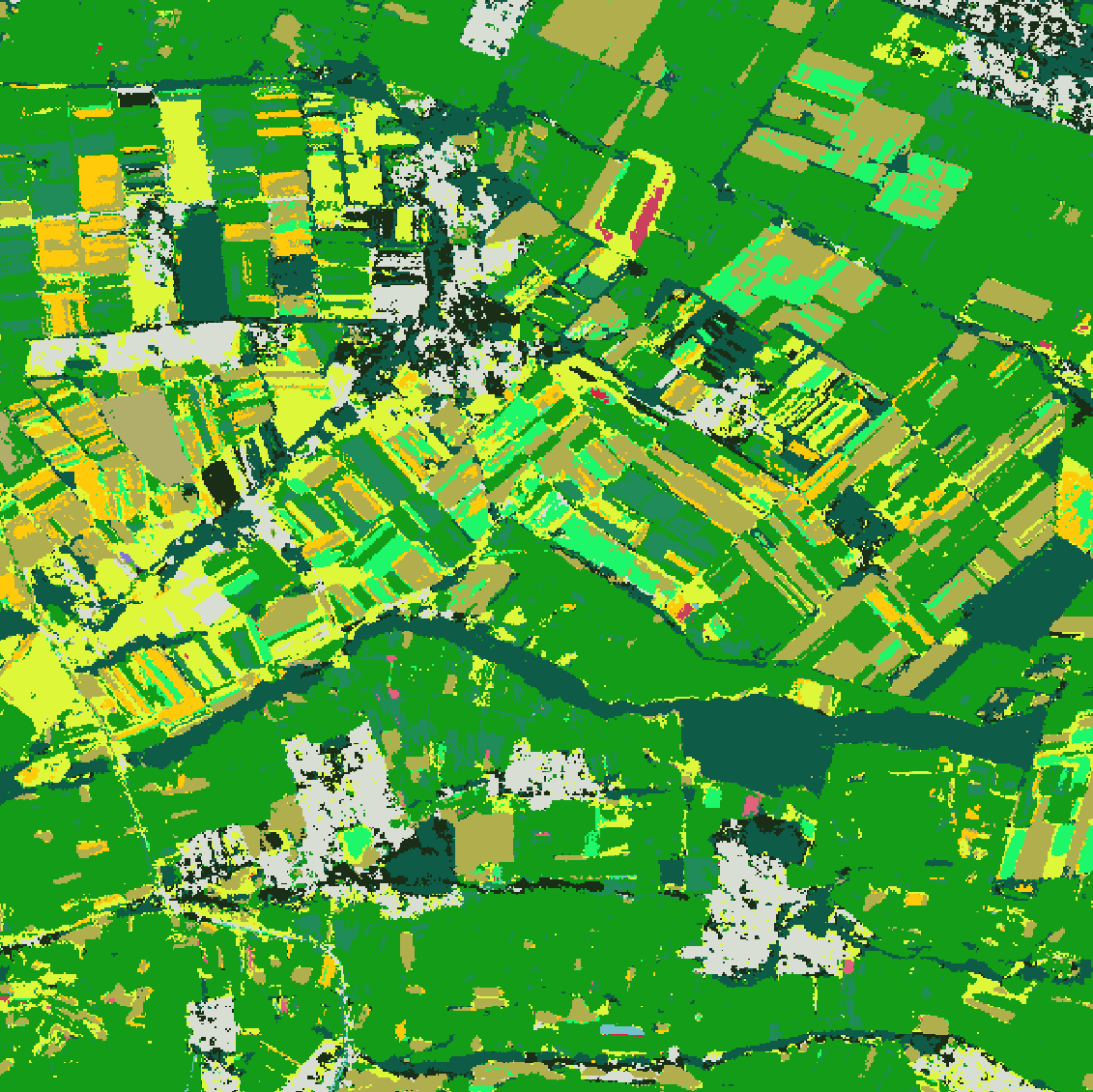


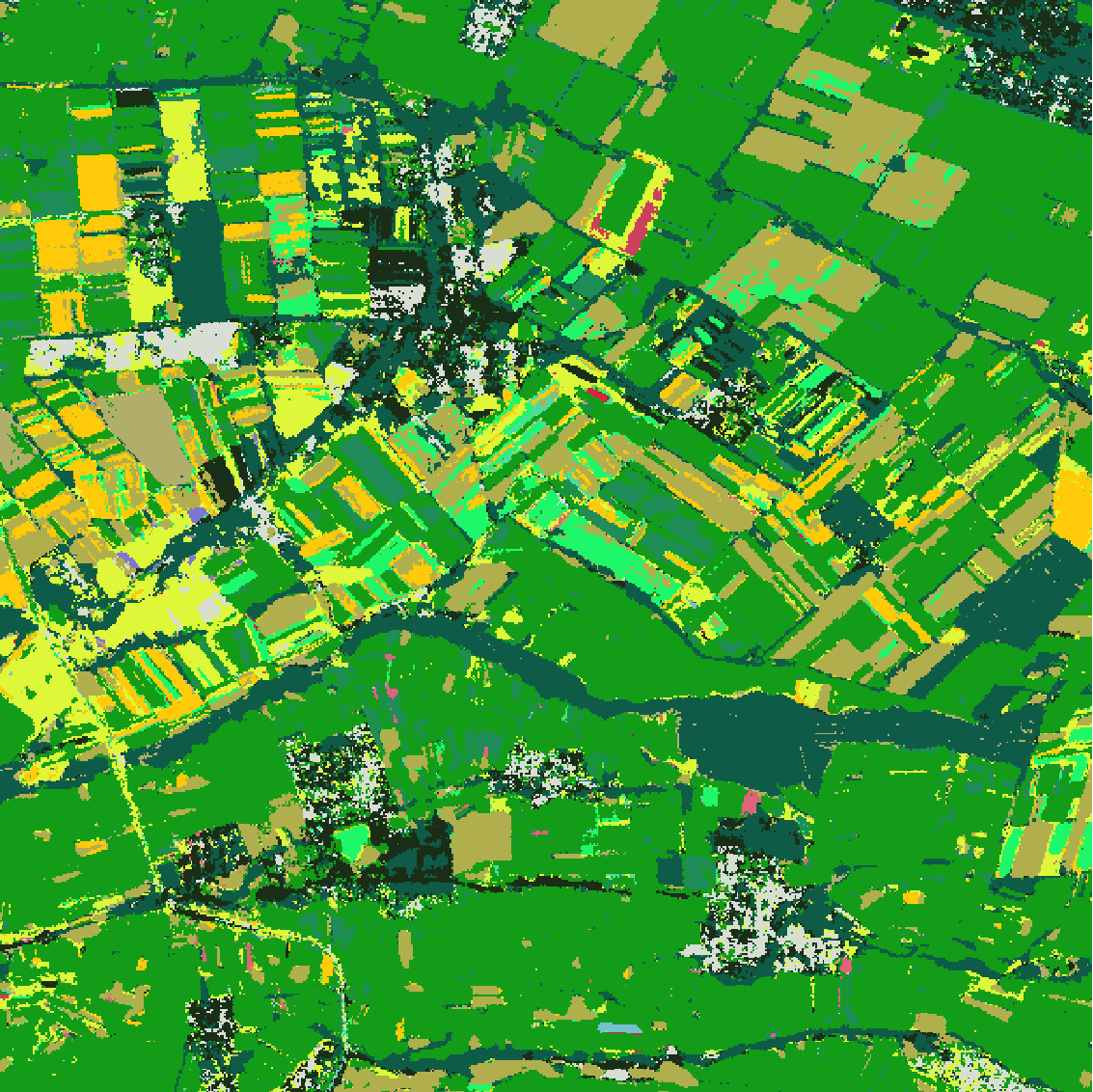

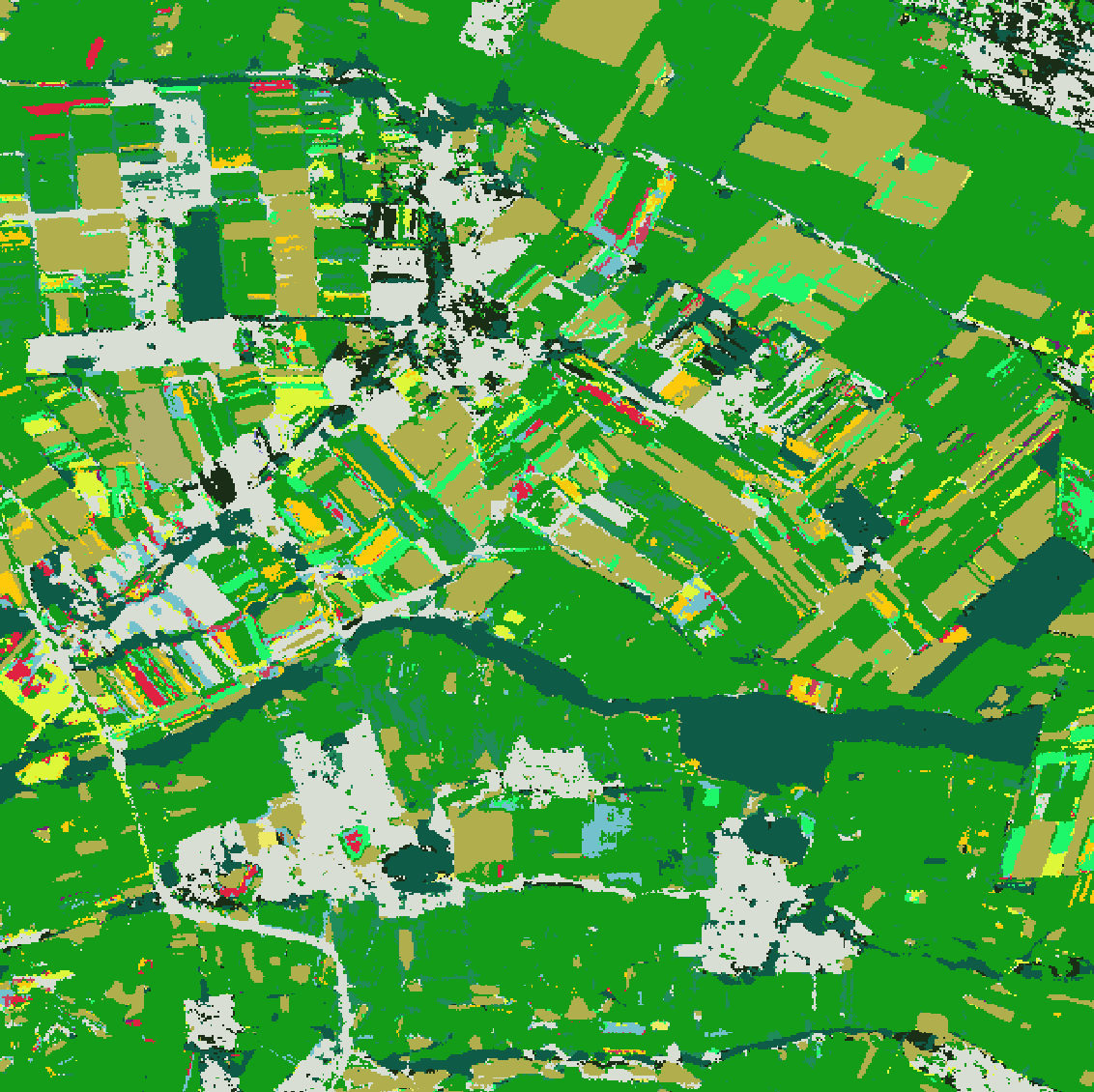

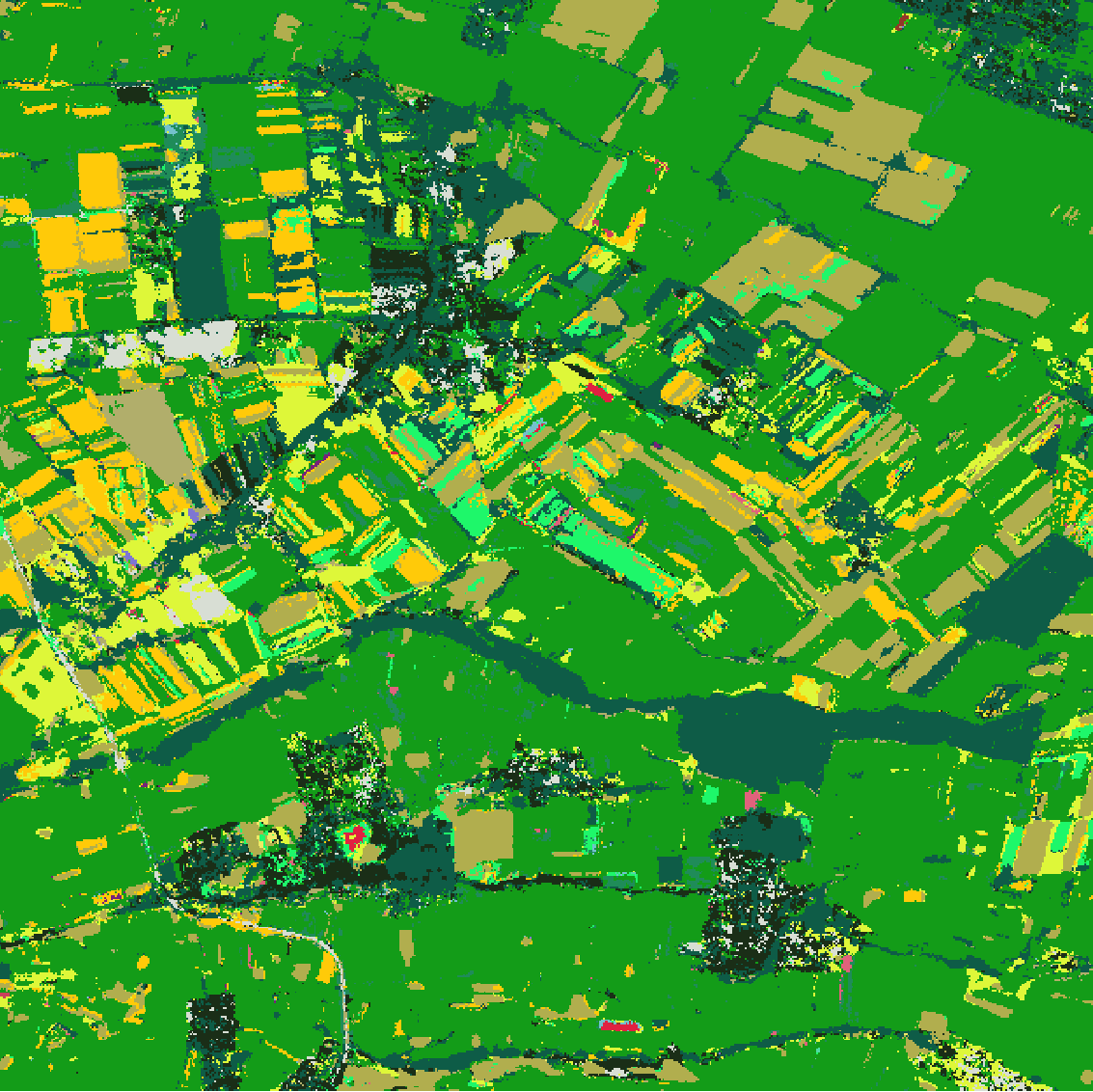


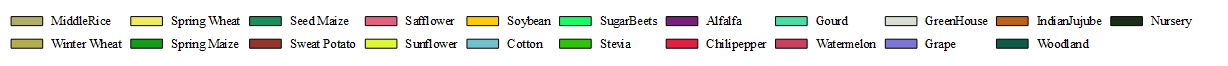


1. Ili River Valley


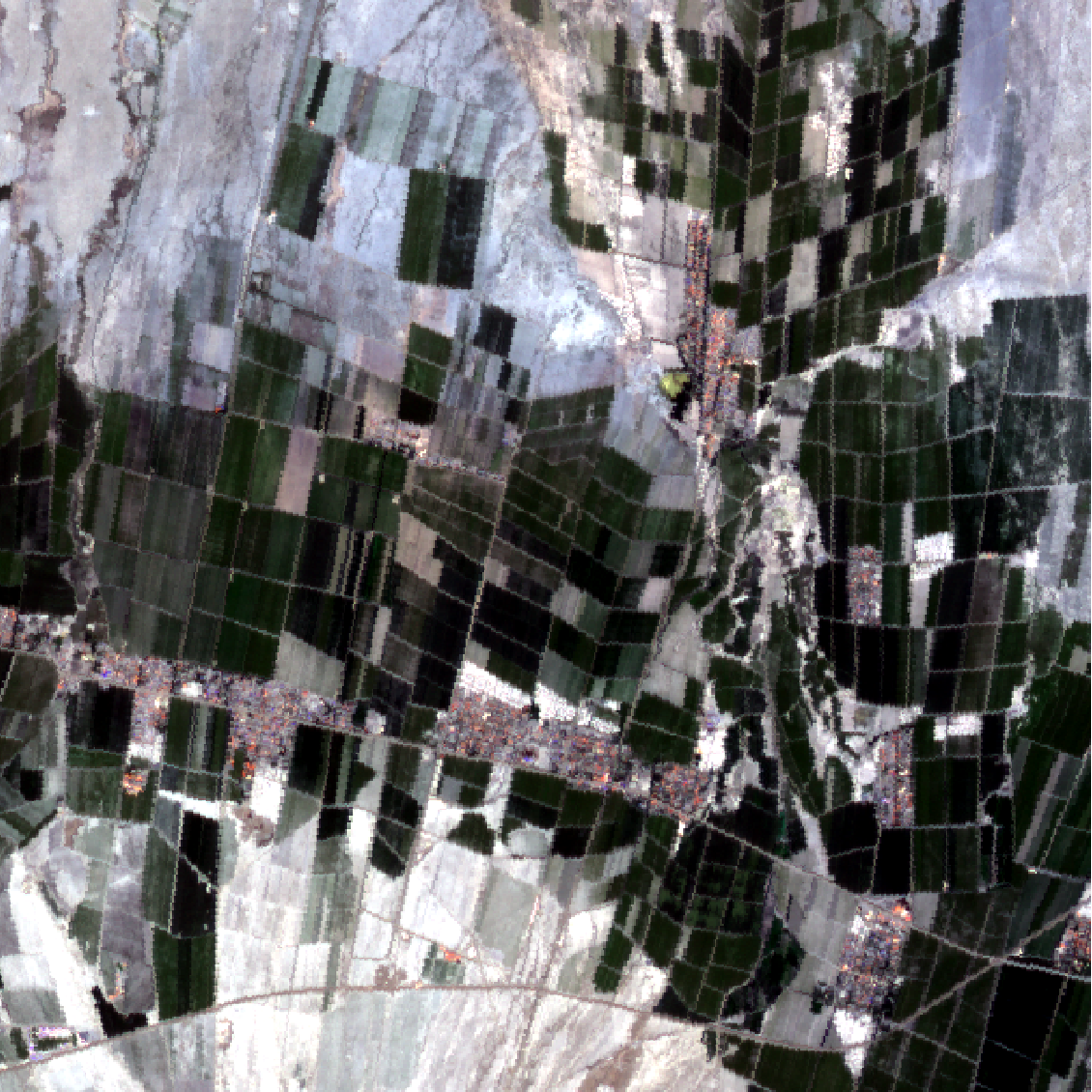

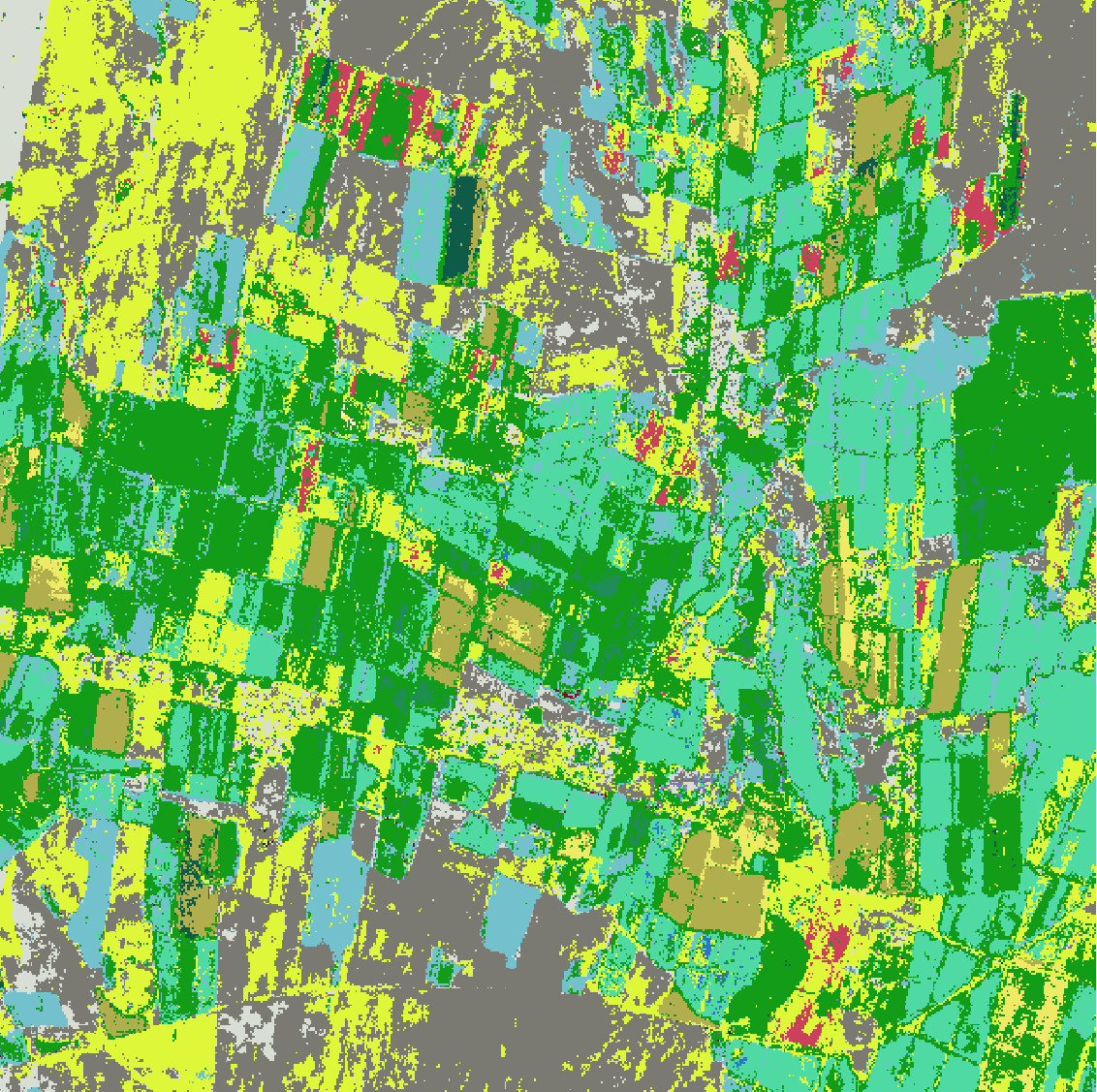

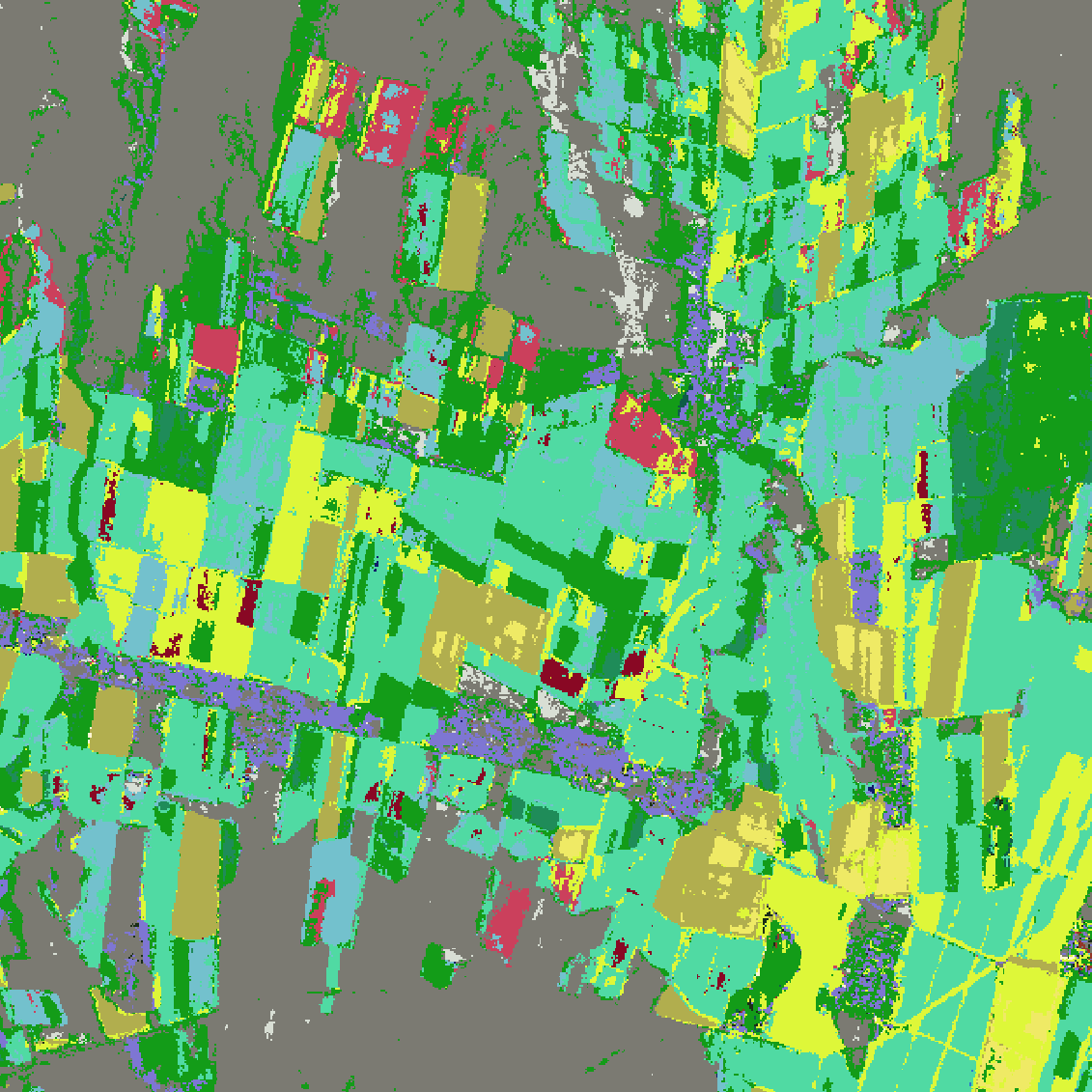


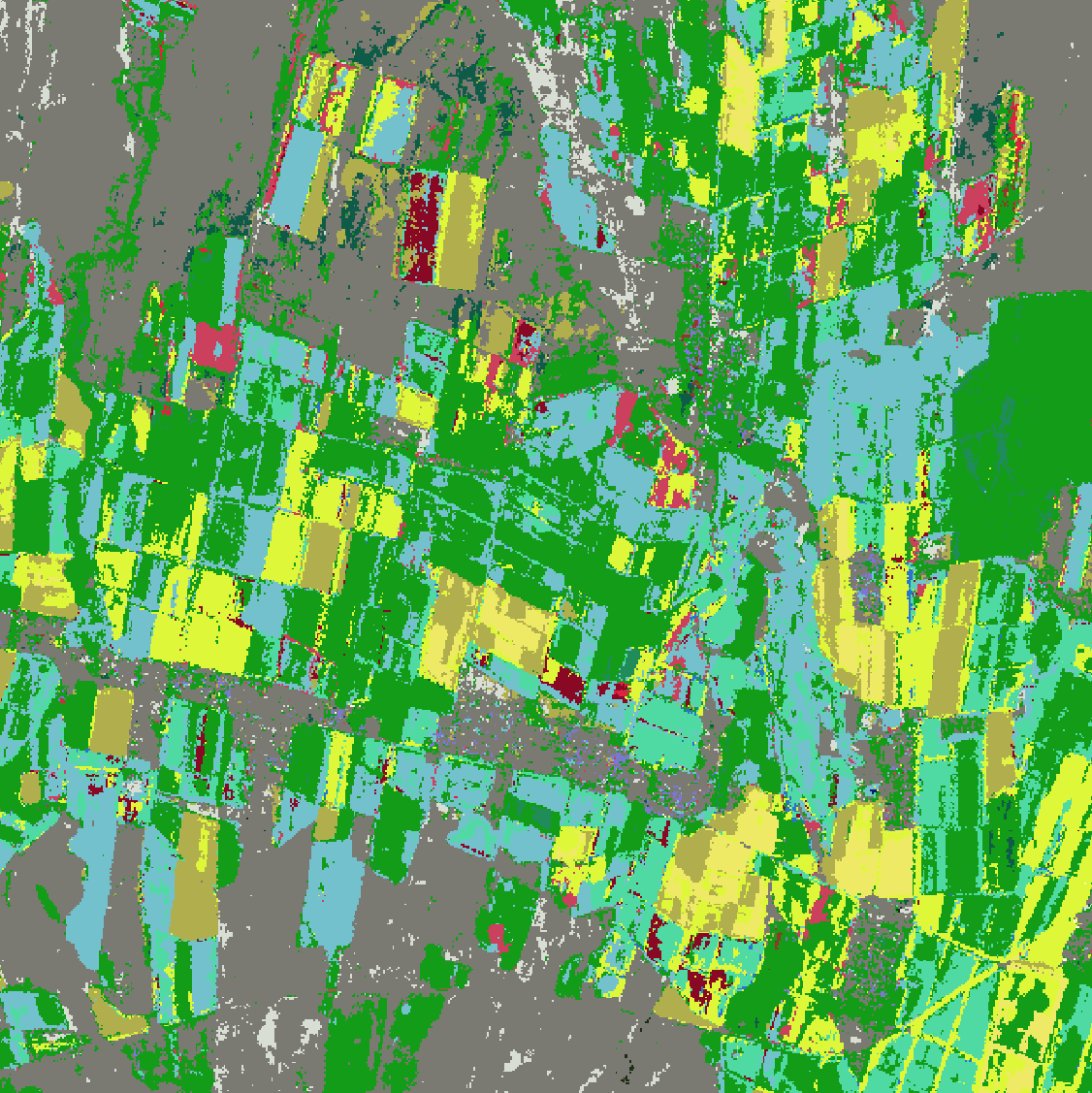

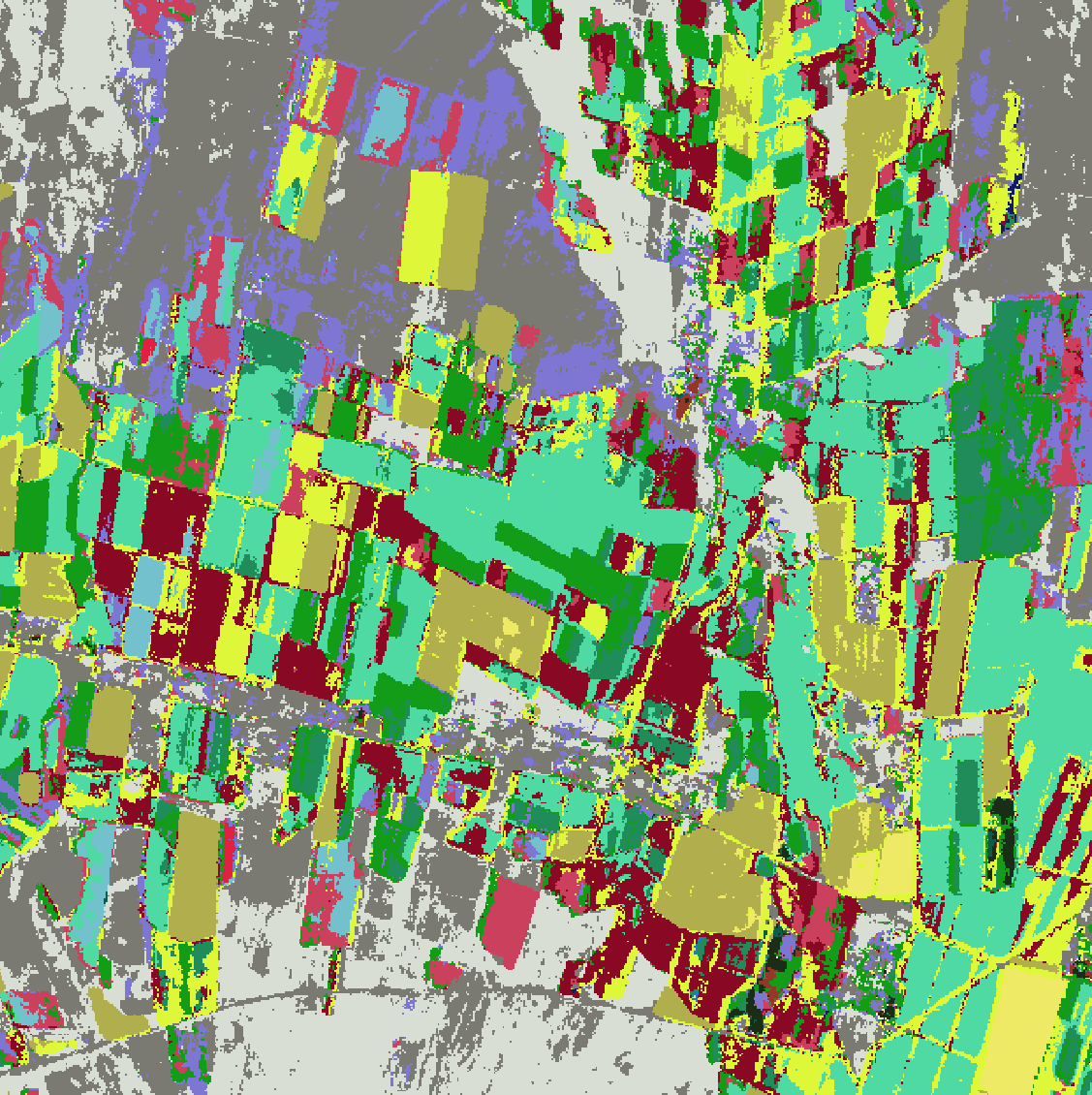

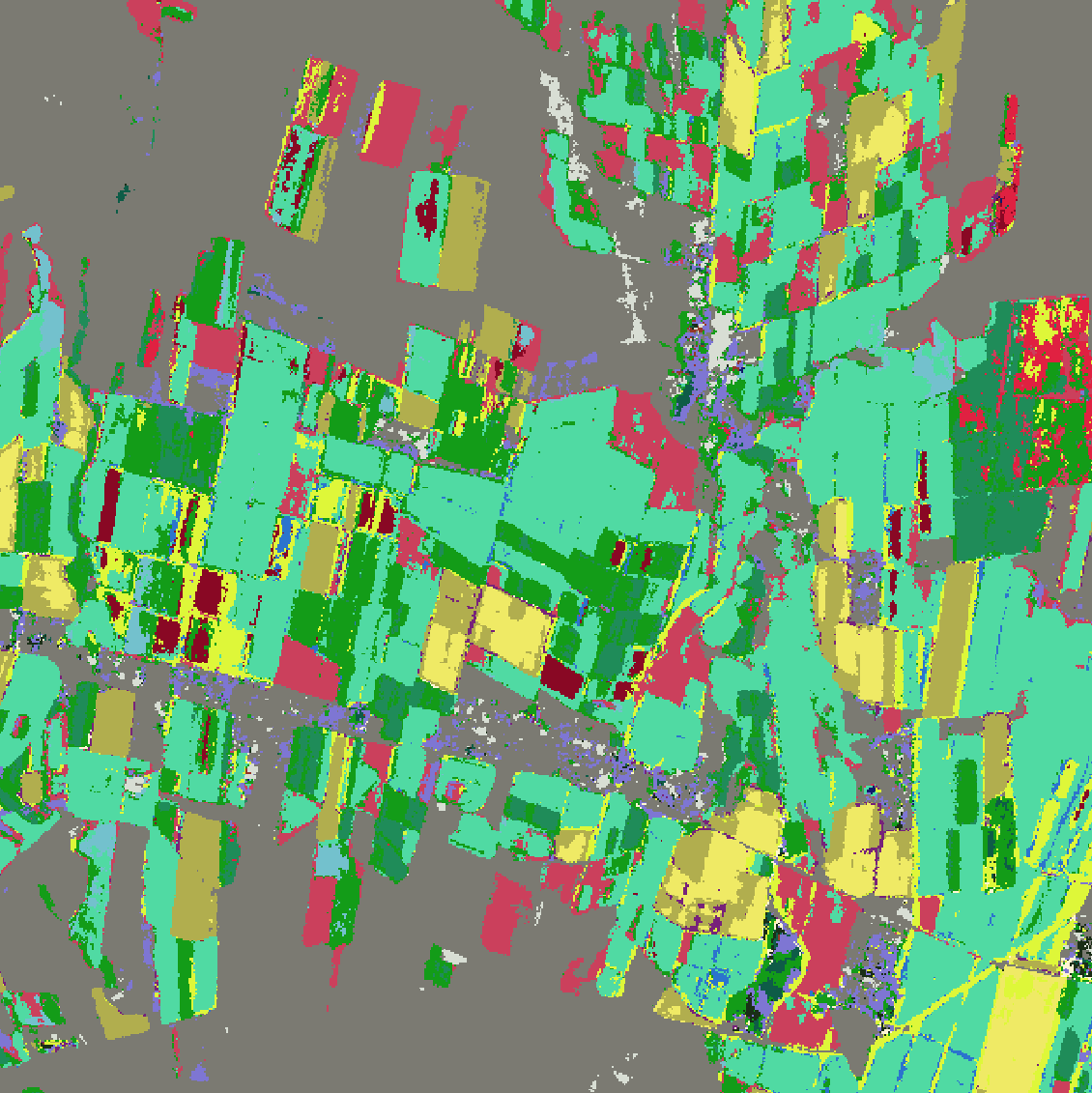


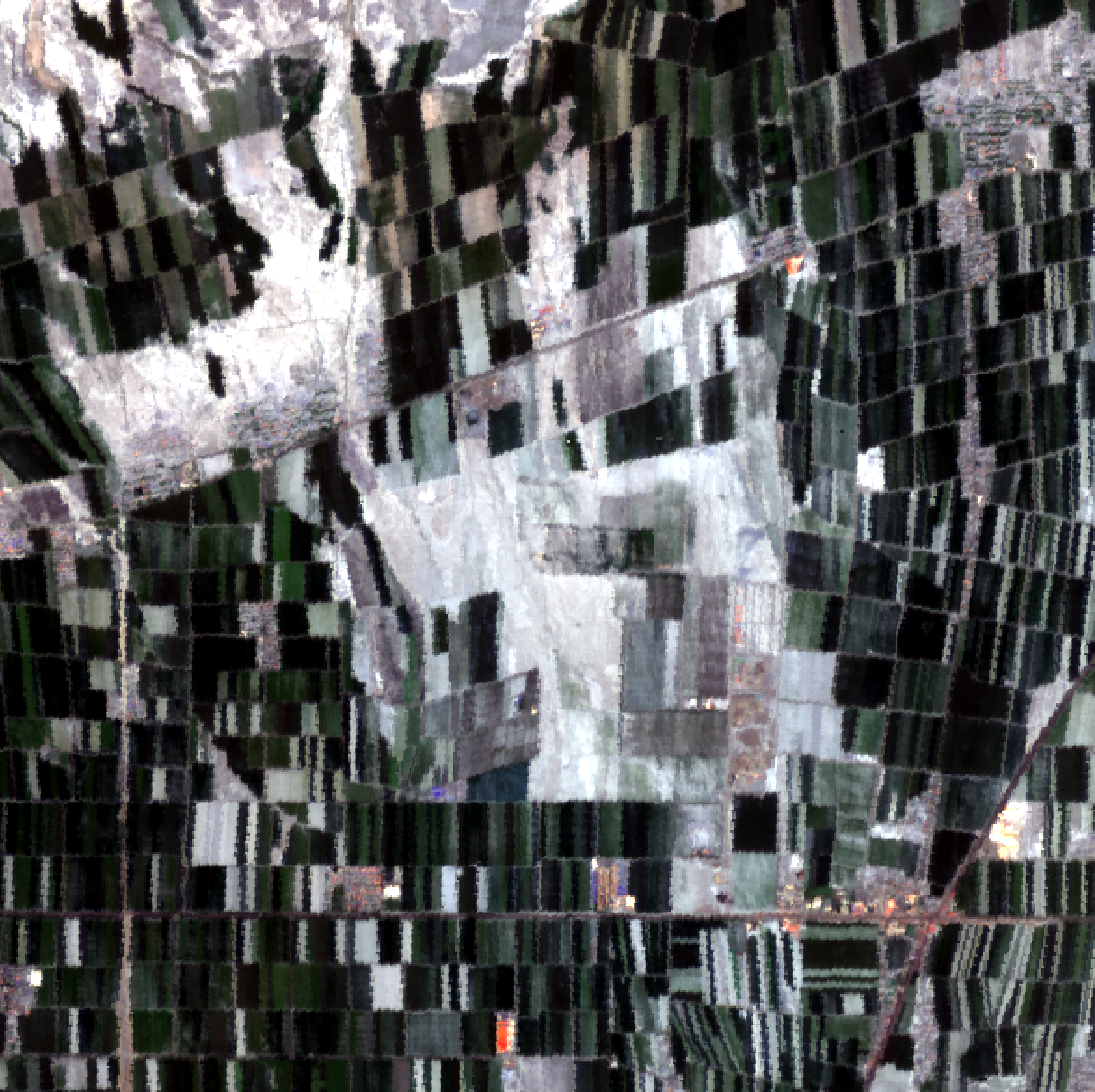

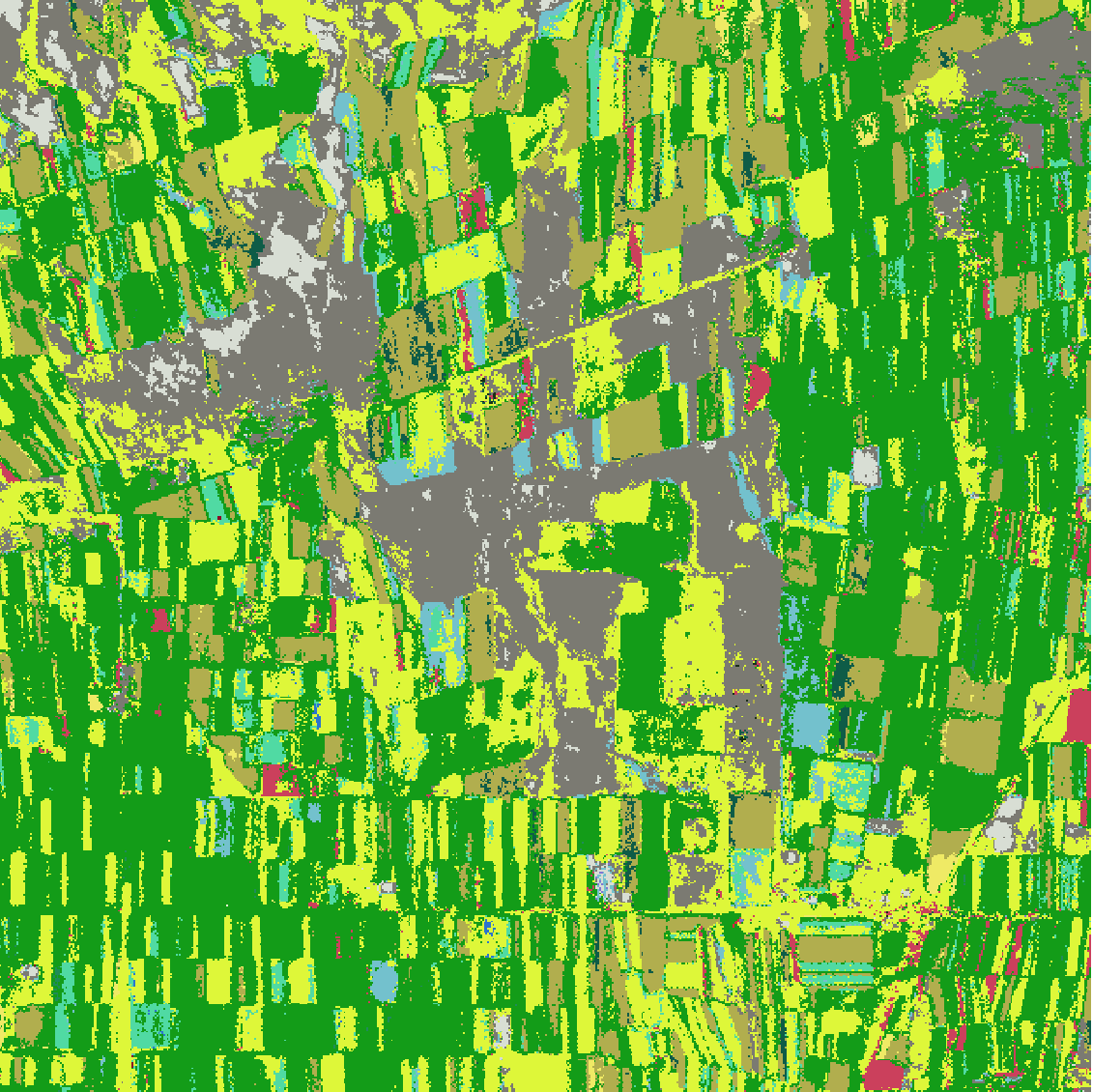

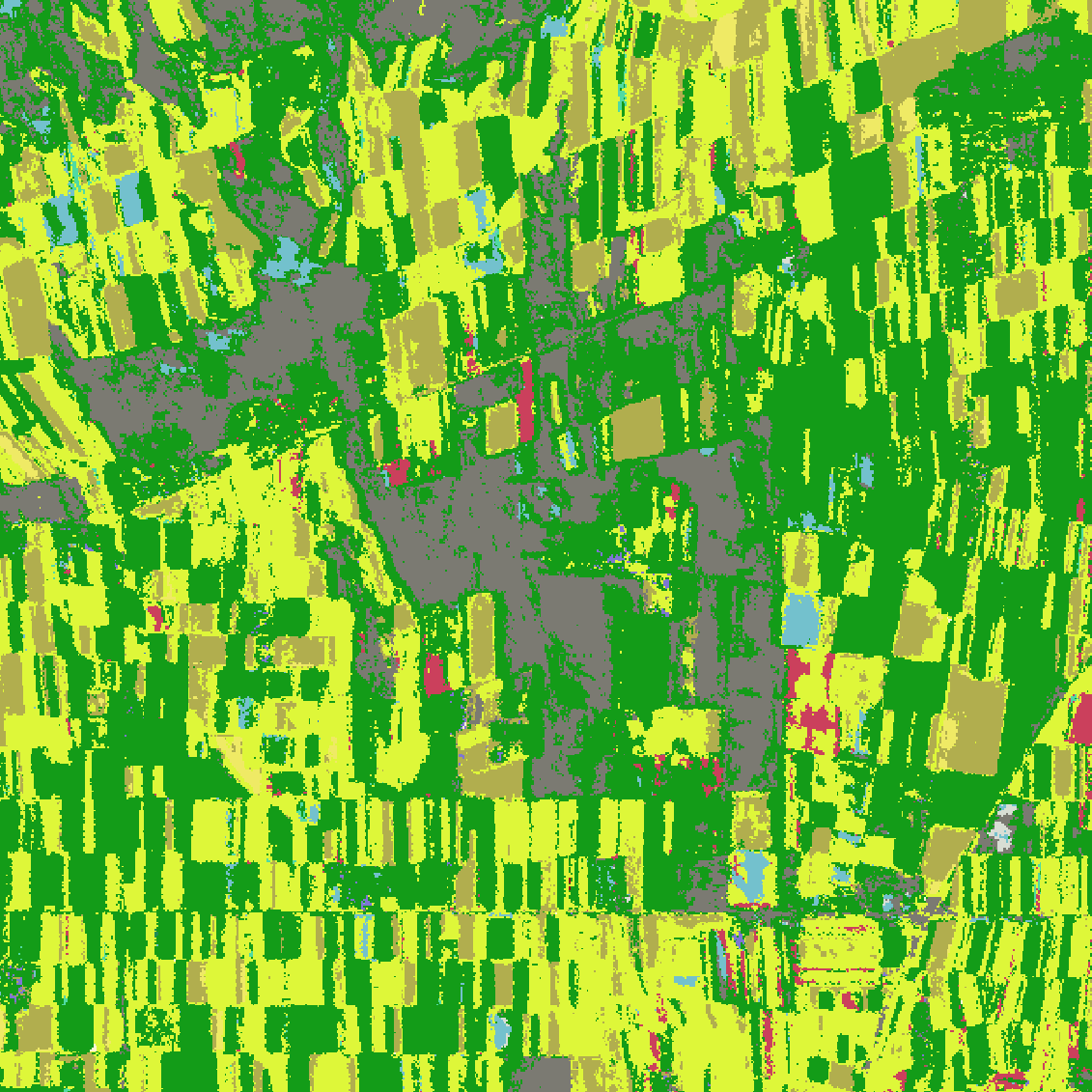


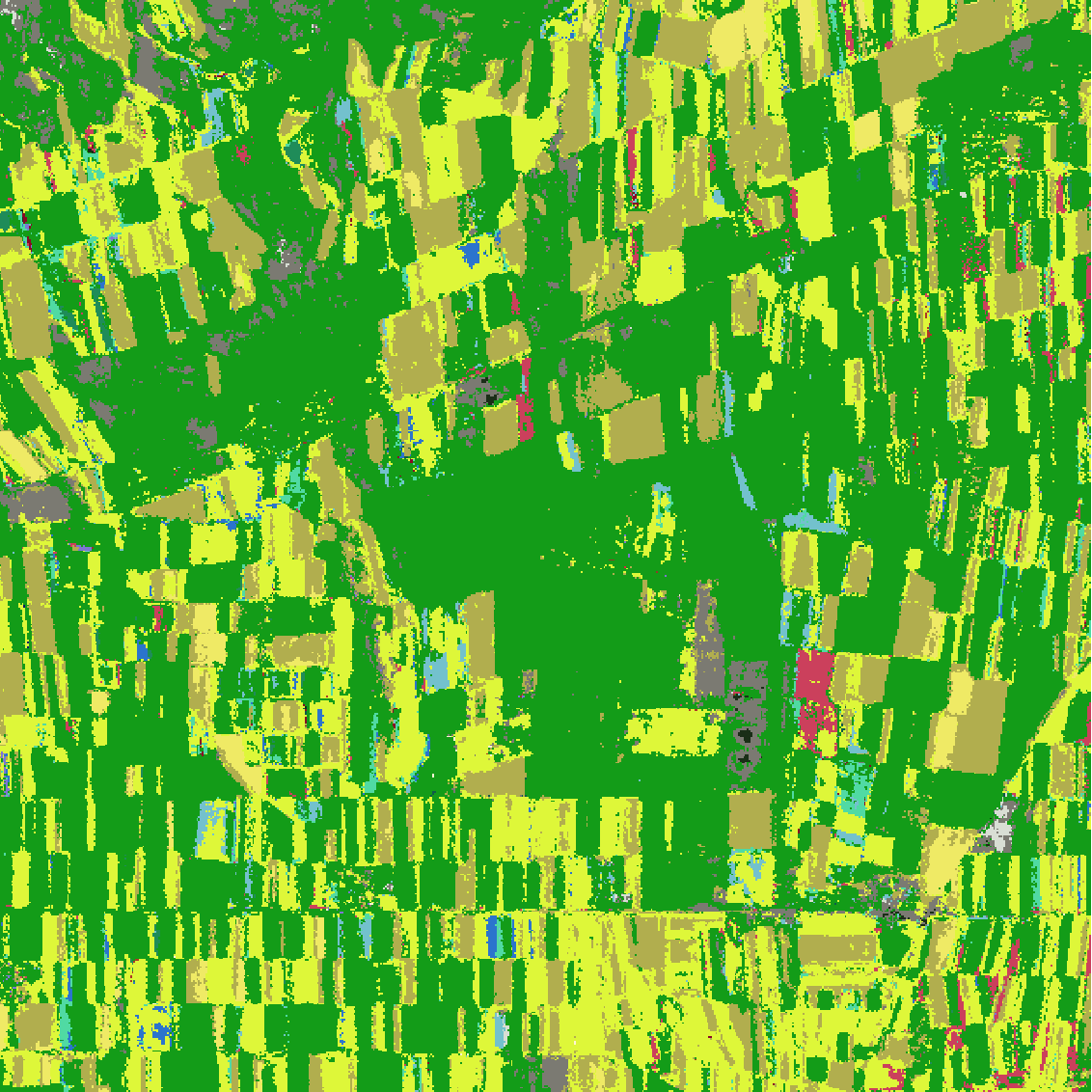

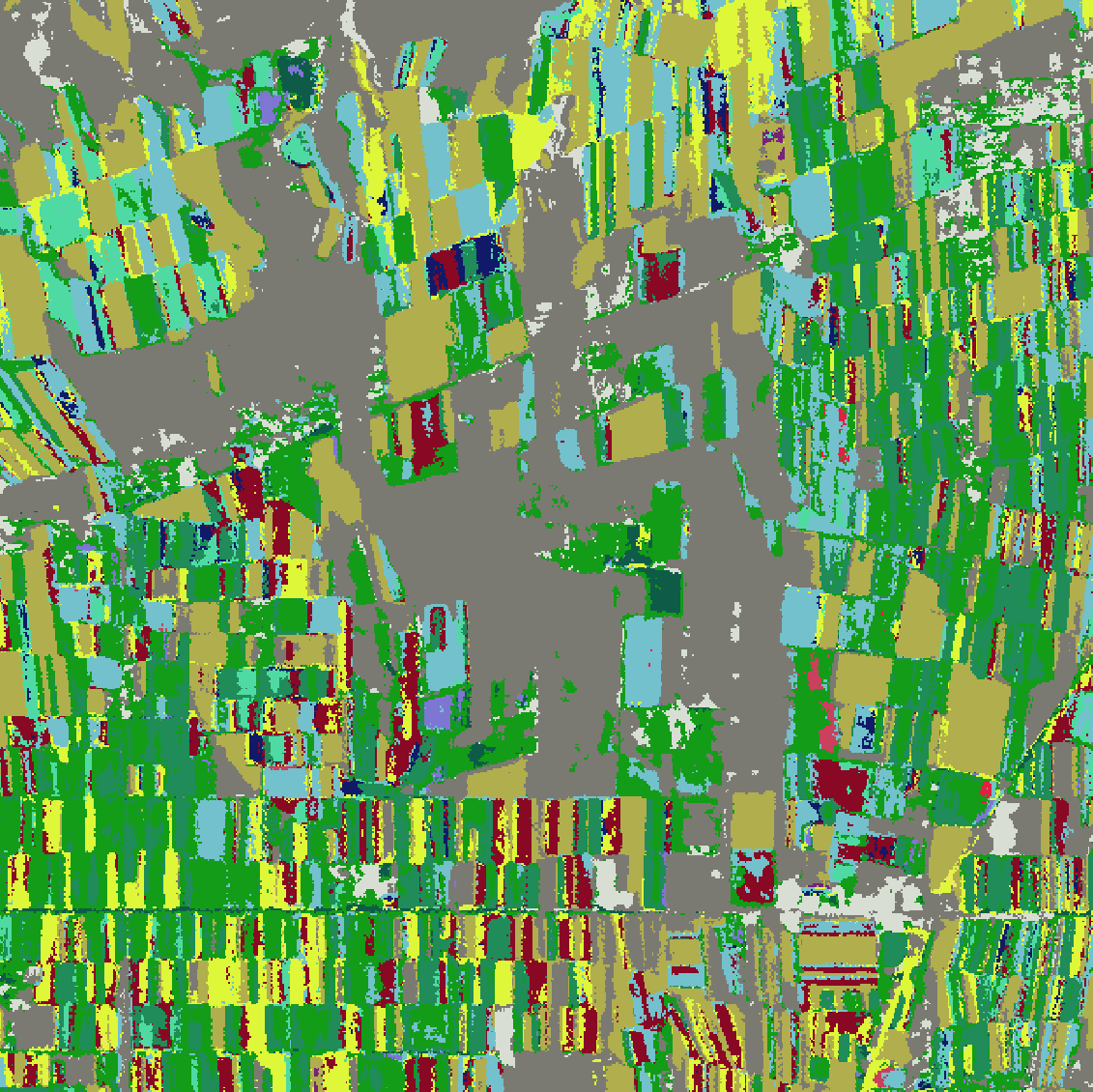

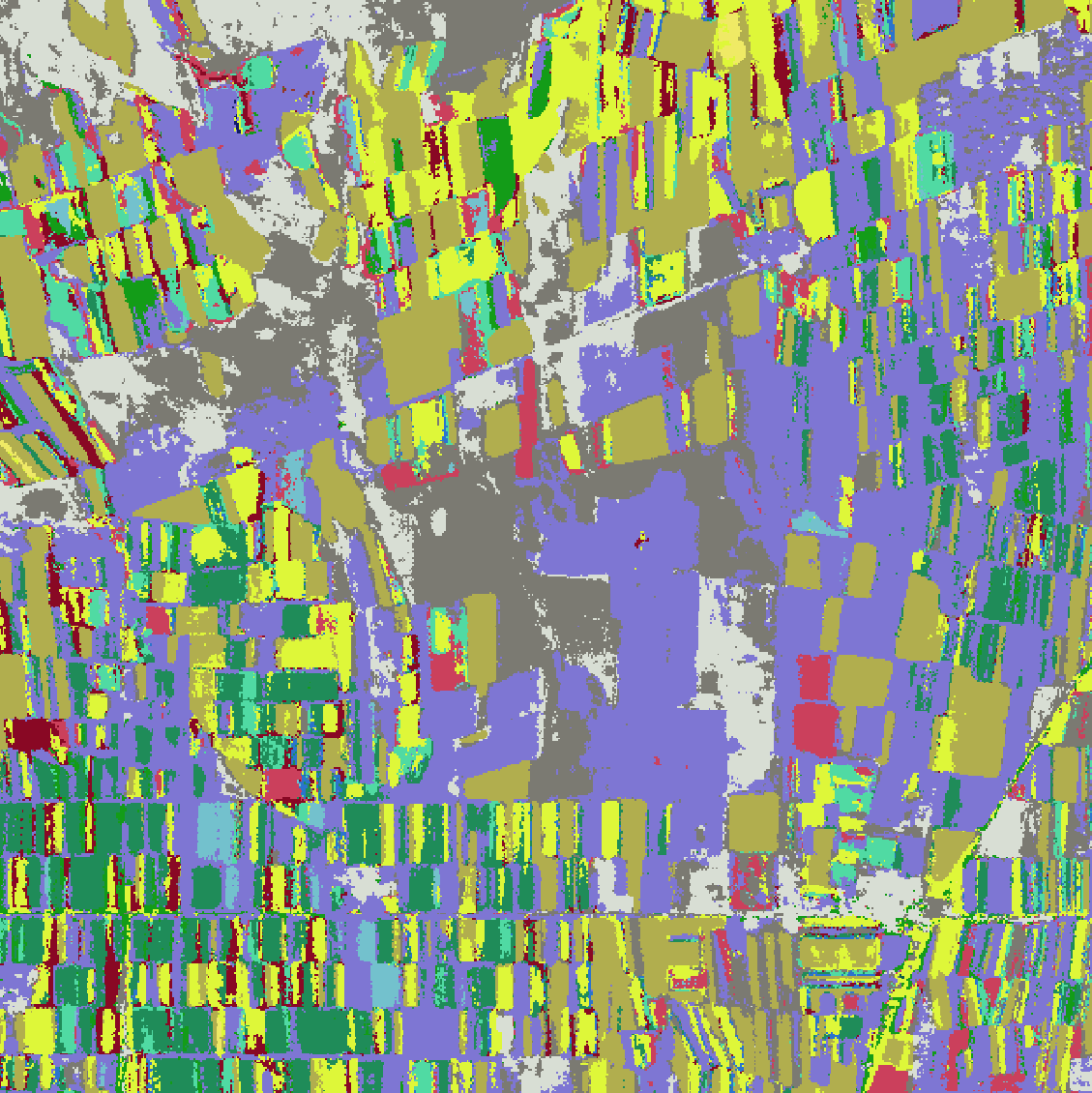


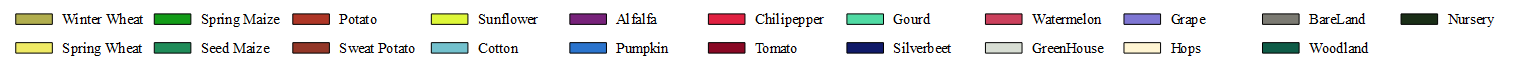


1. Tianshan Corridor


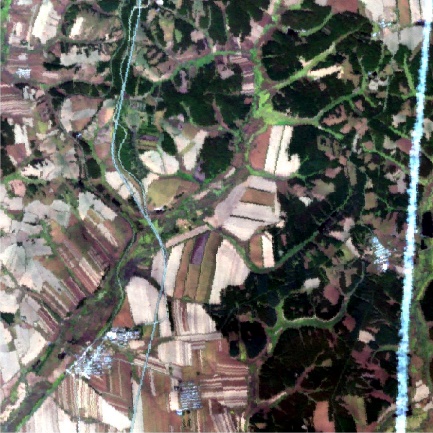

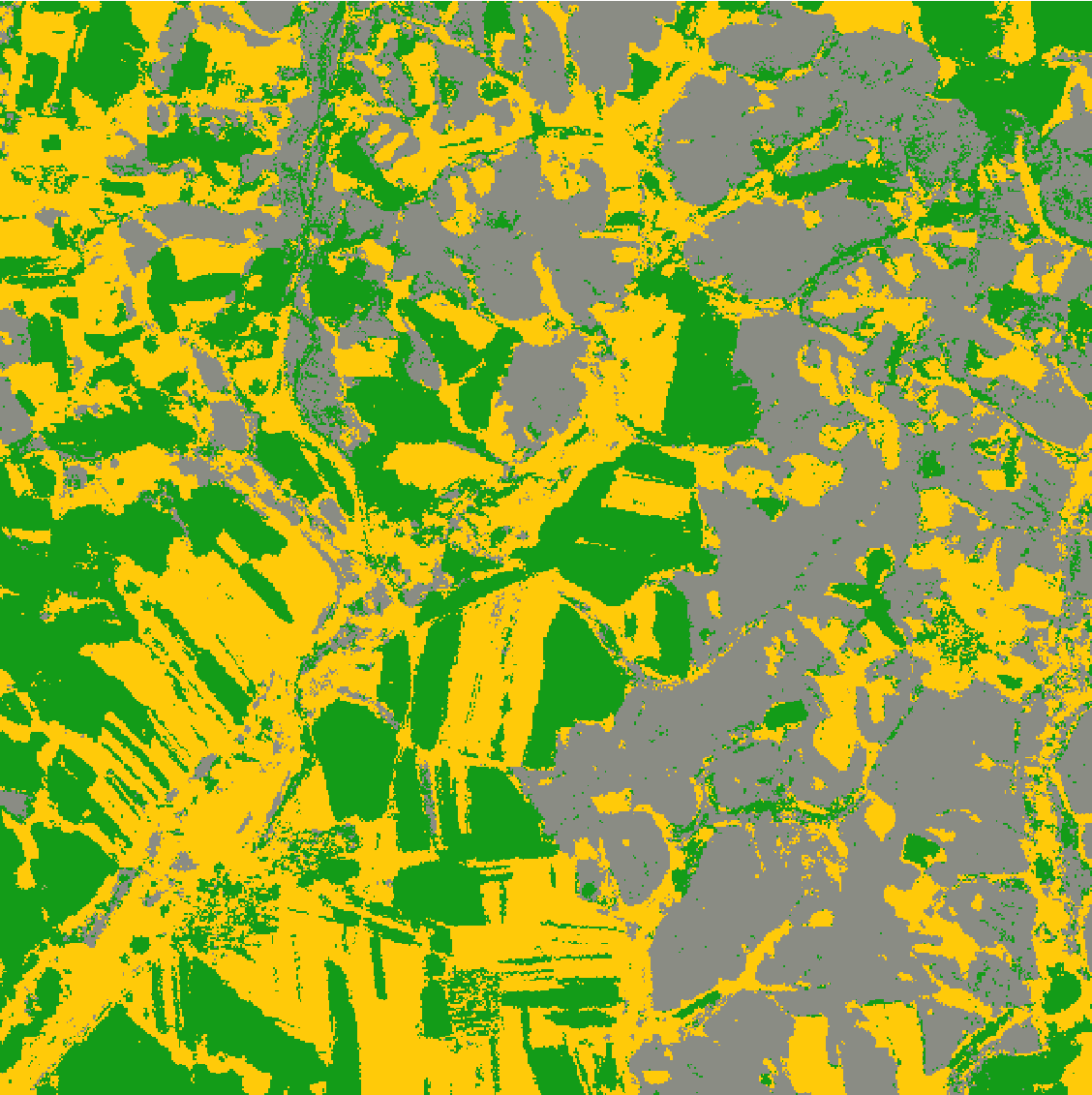

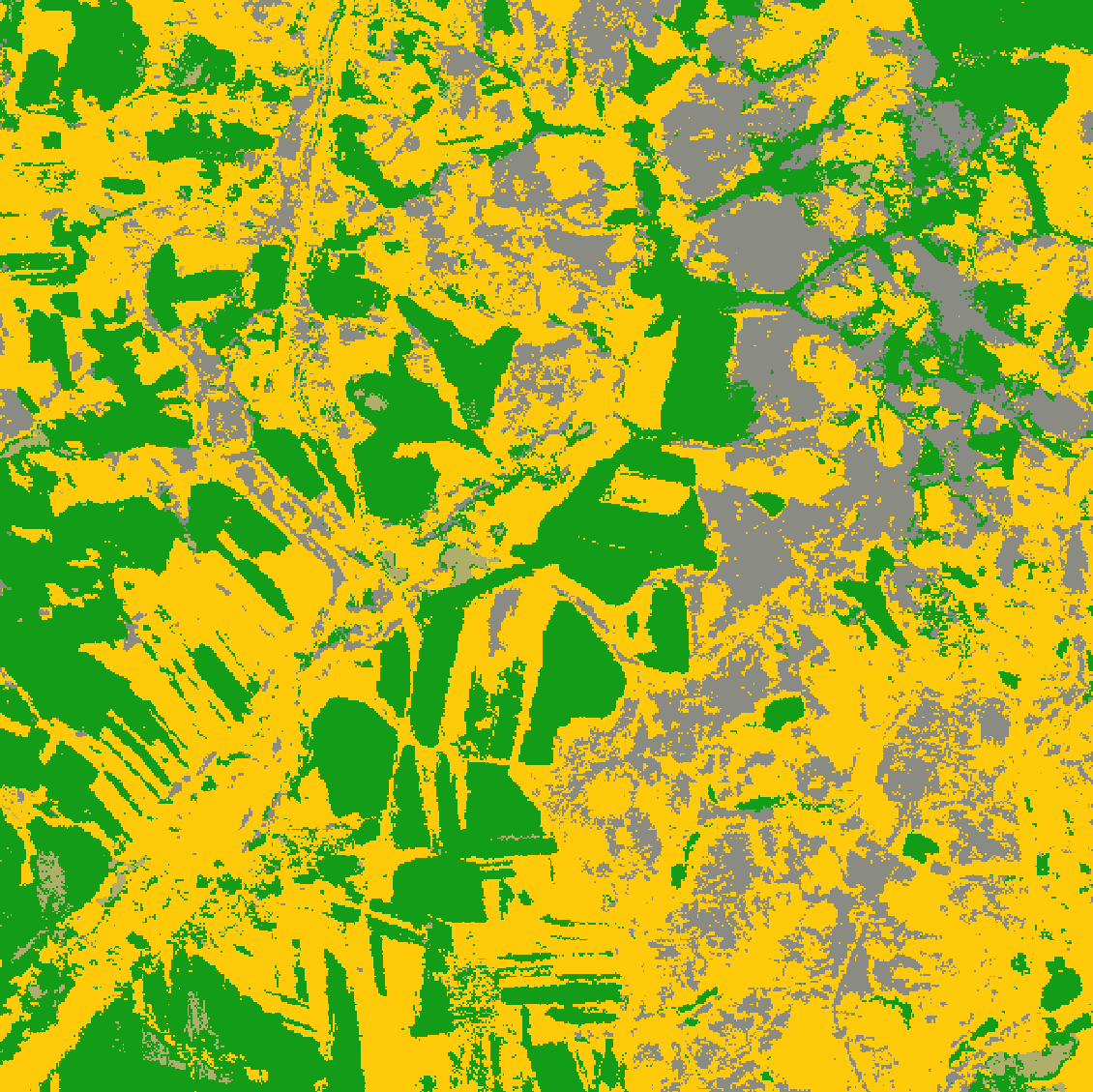


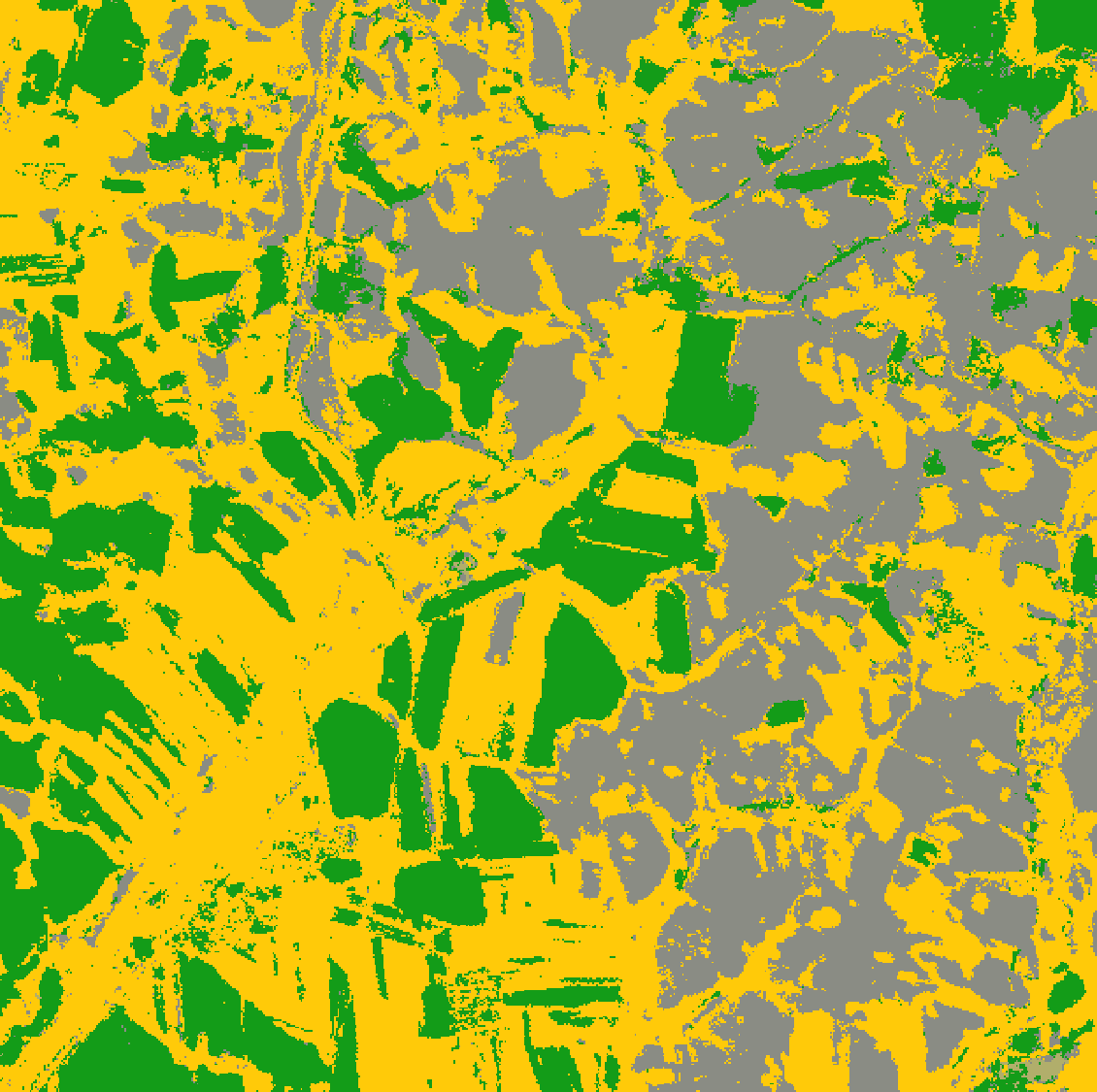

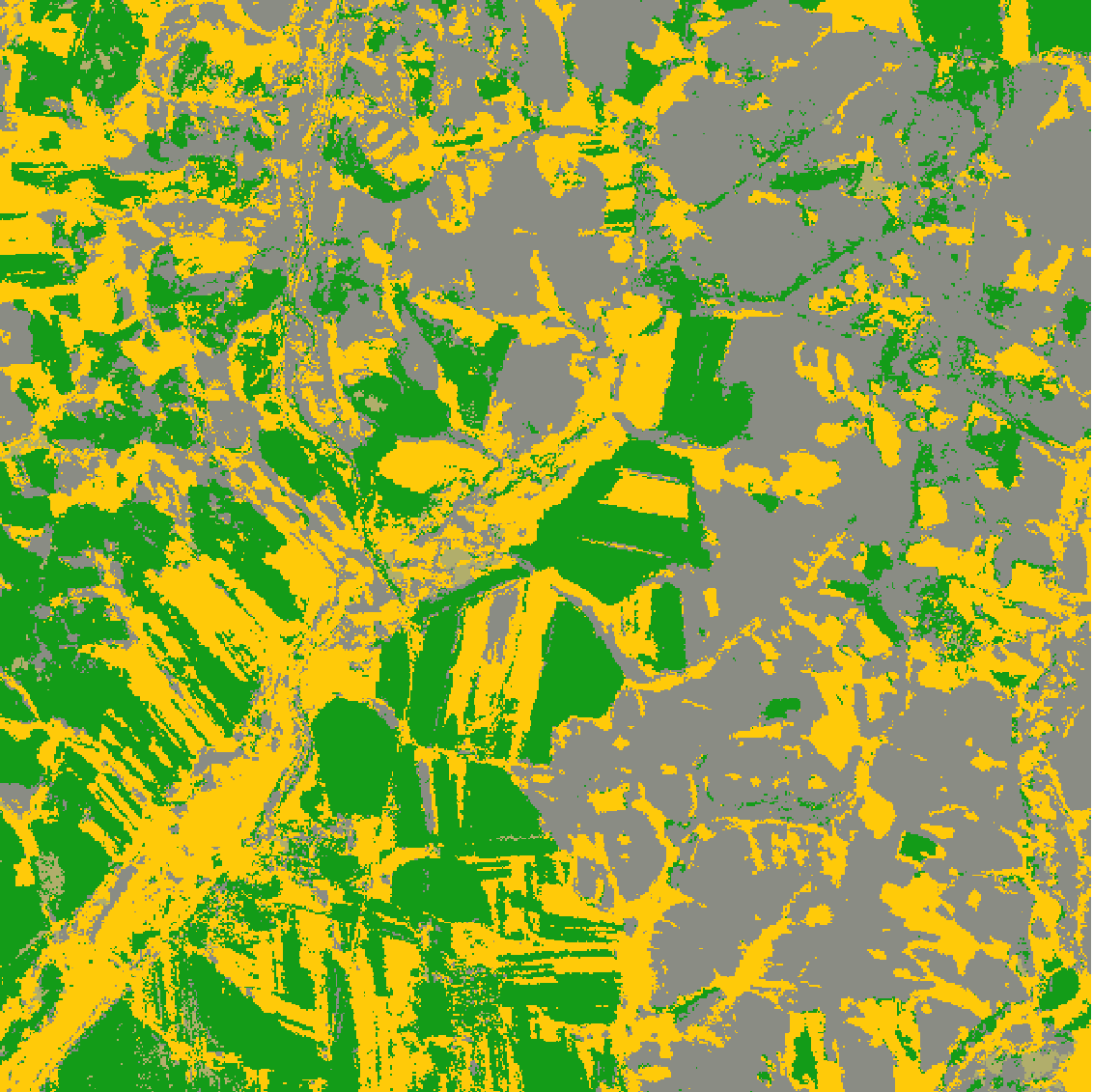

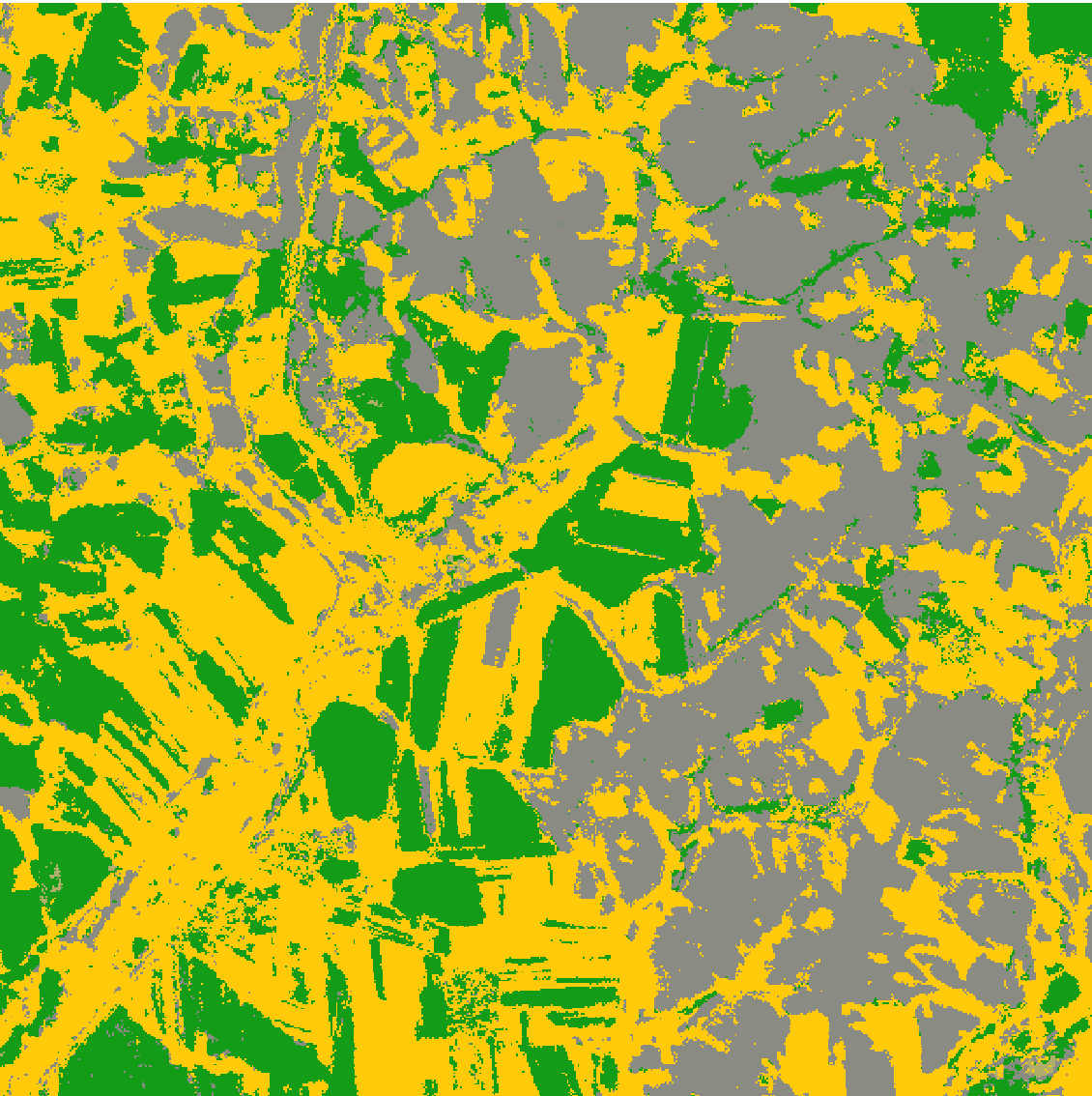


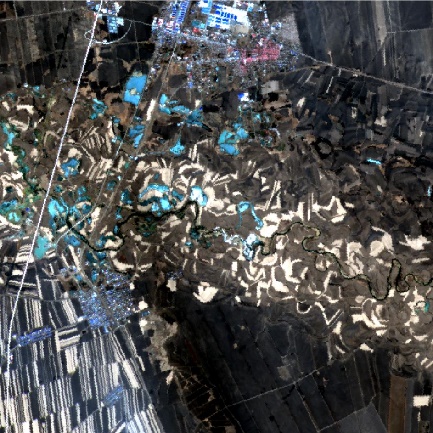

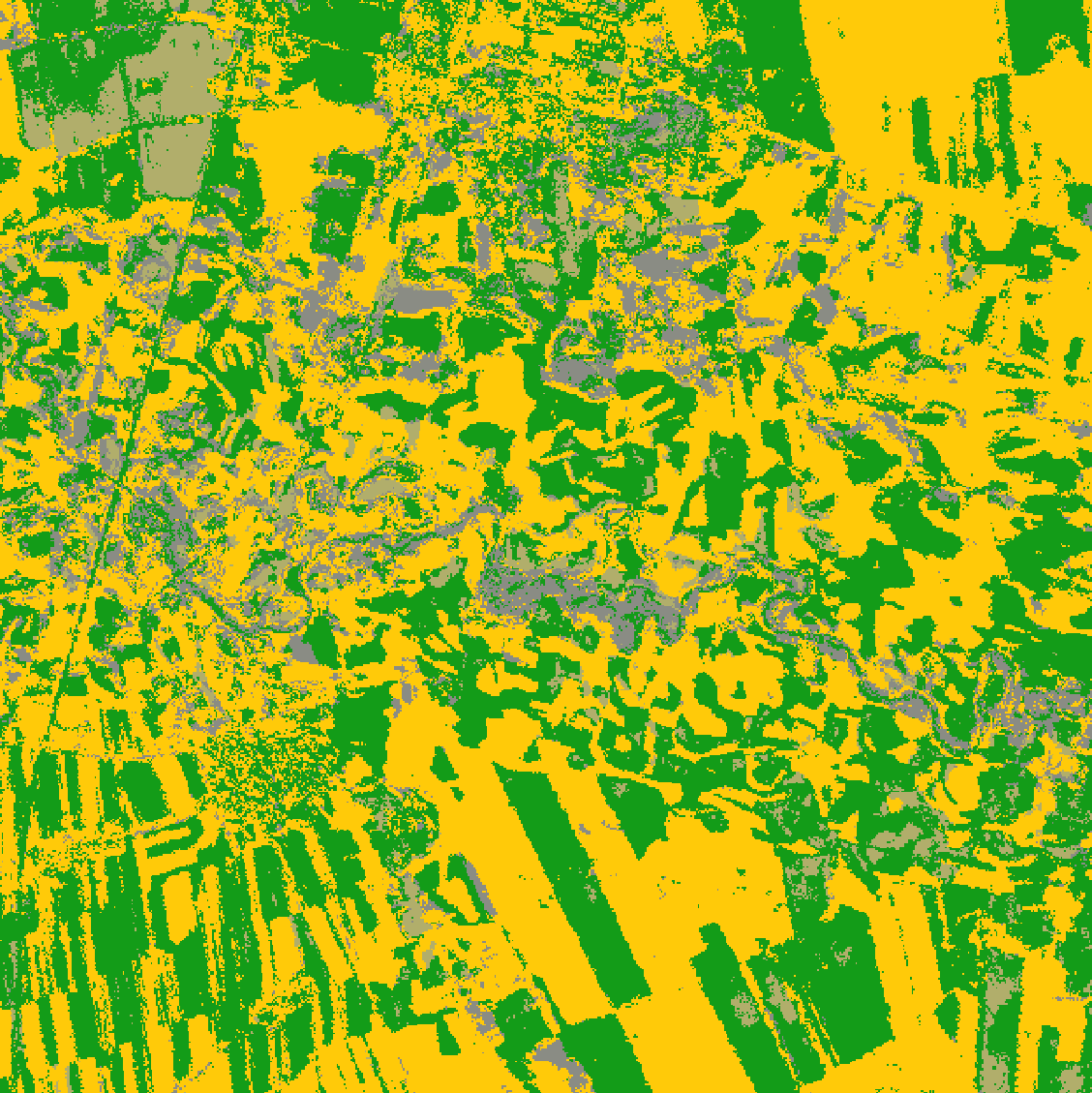

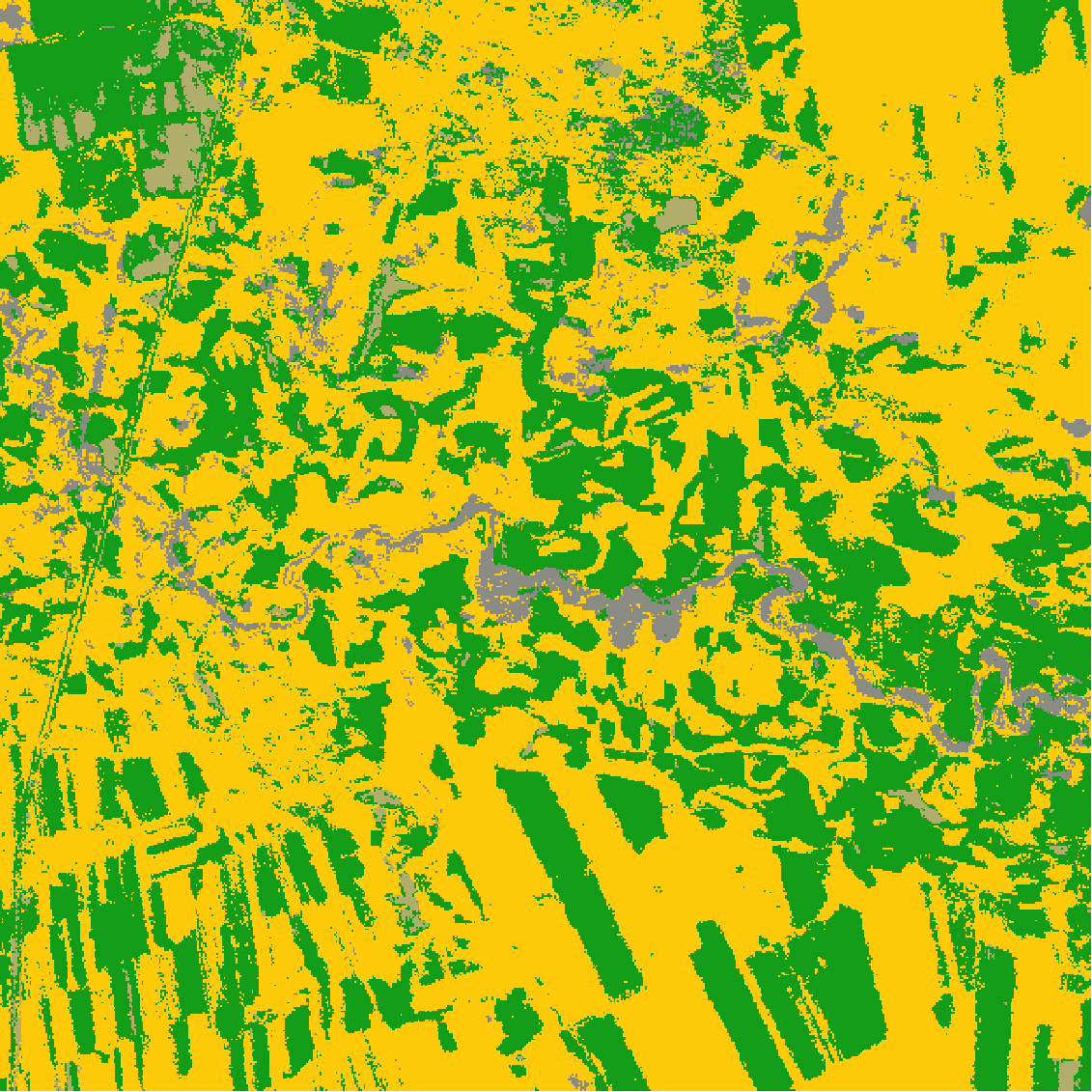


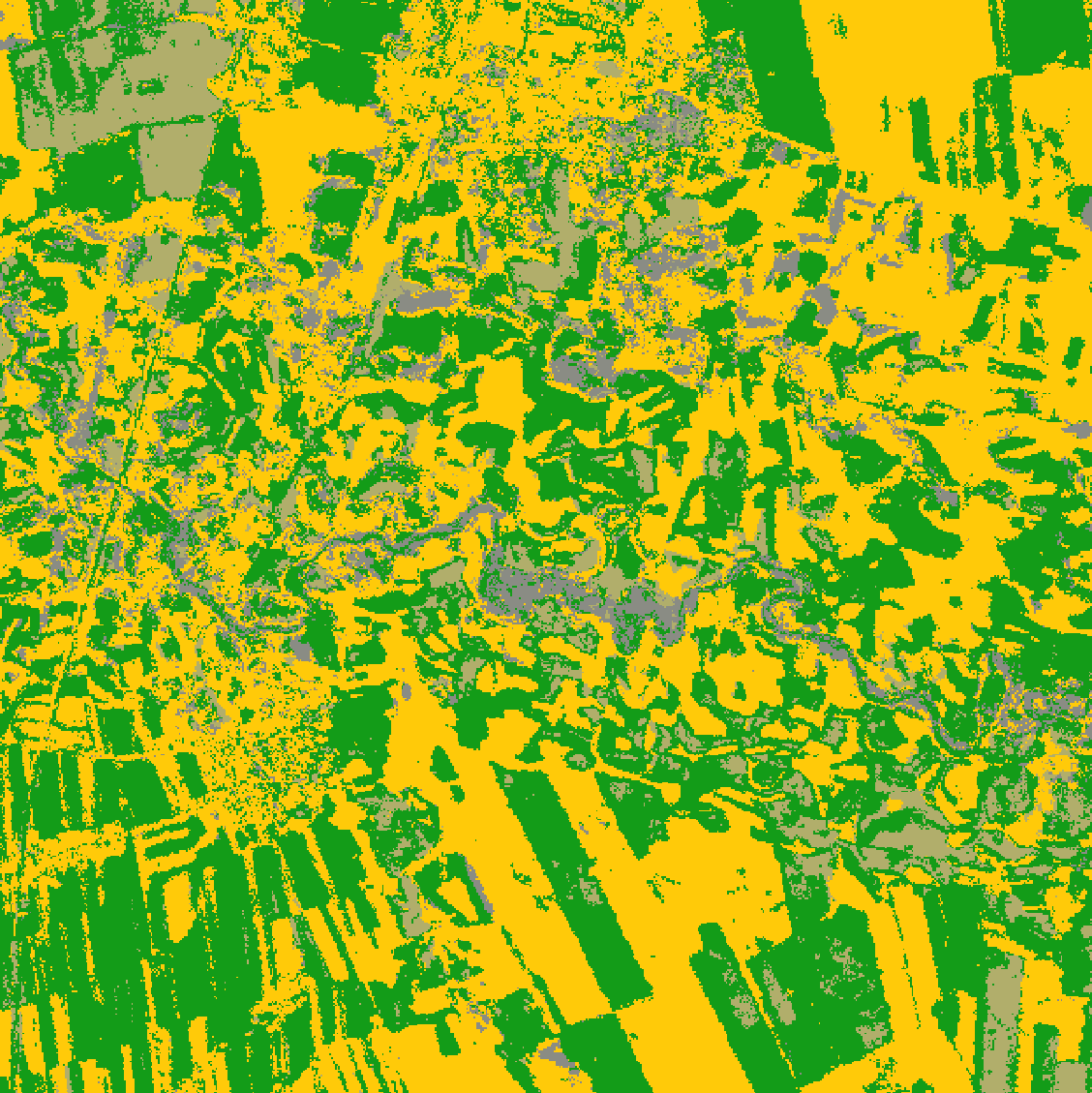

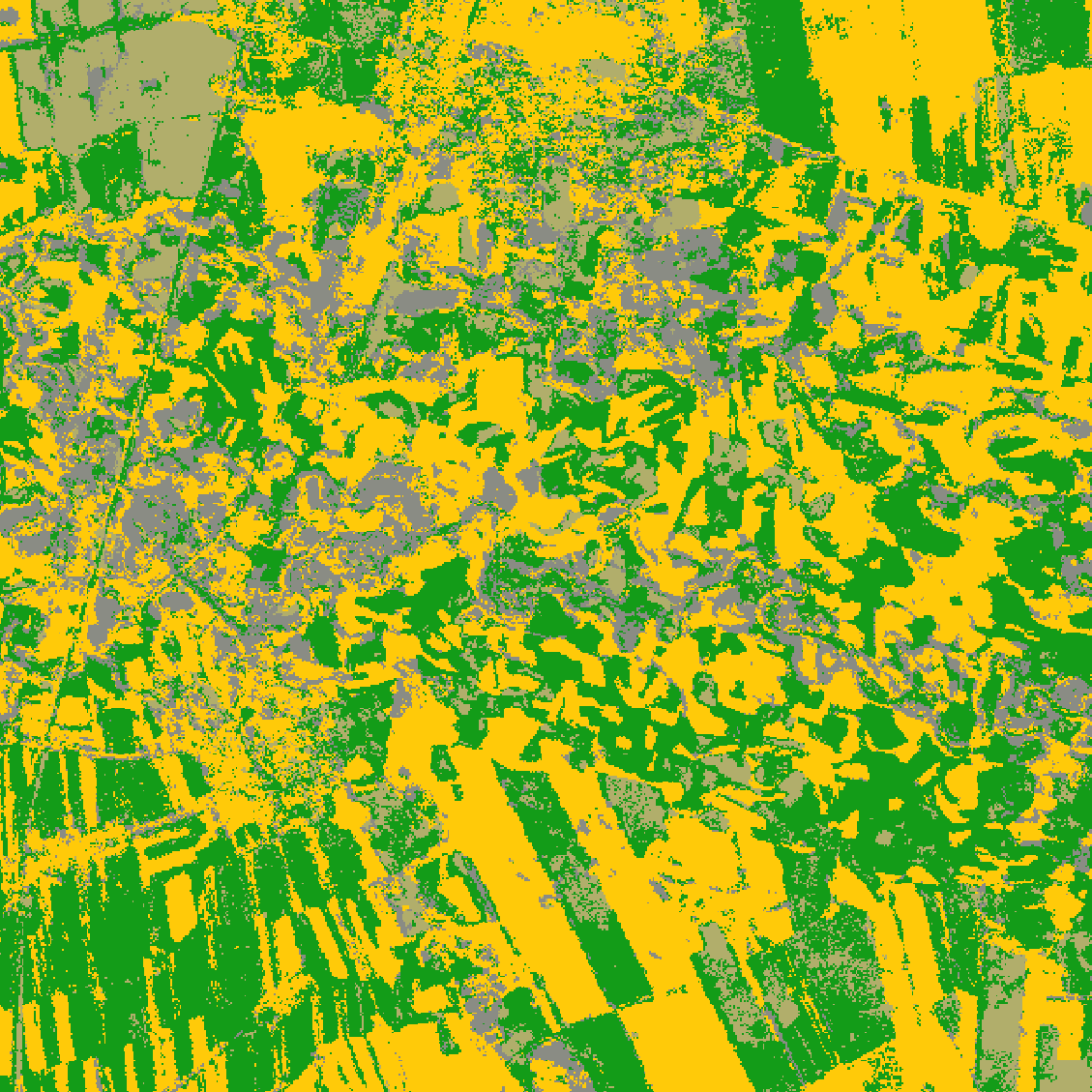

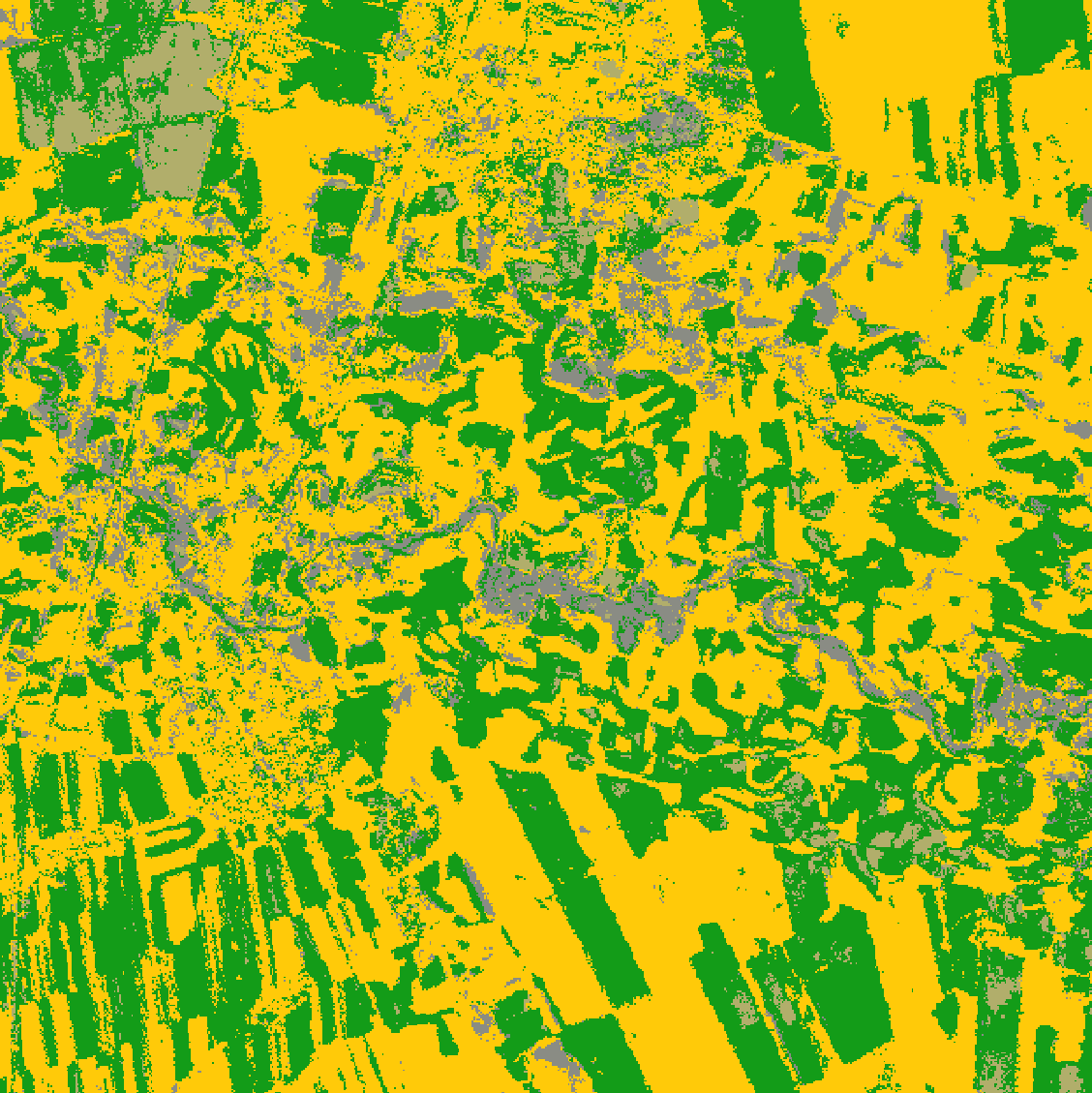


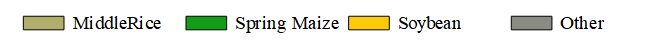


1. Western Heilongjiang


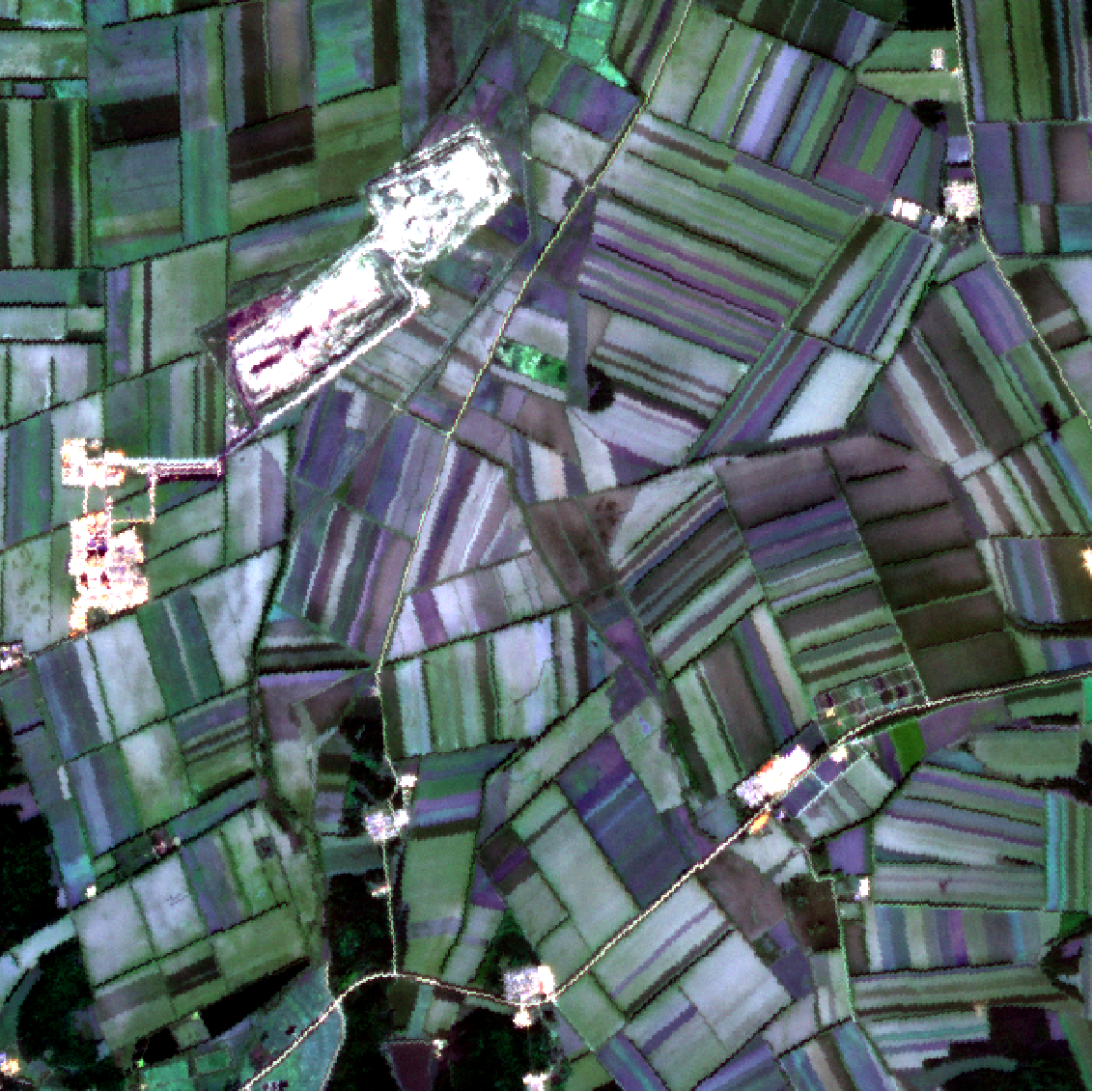

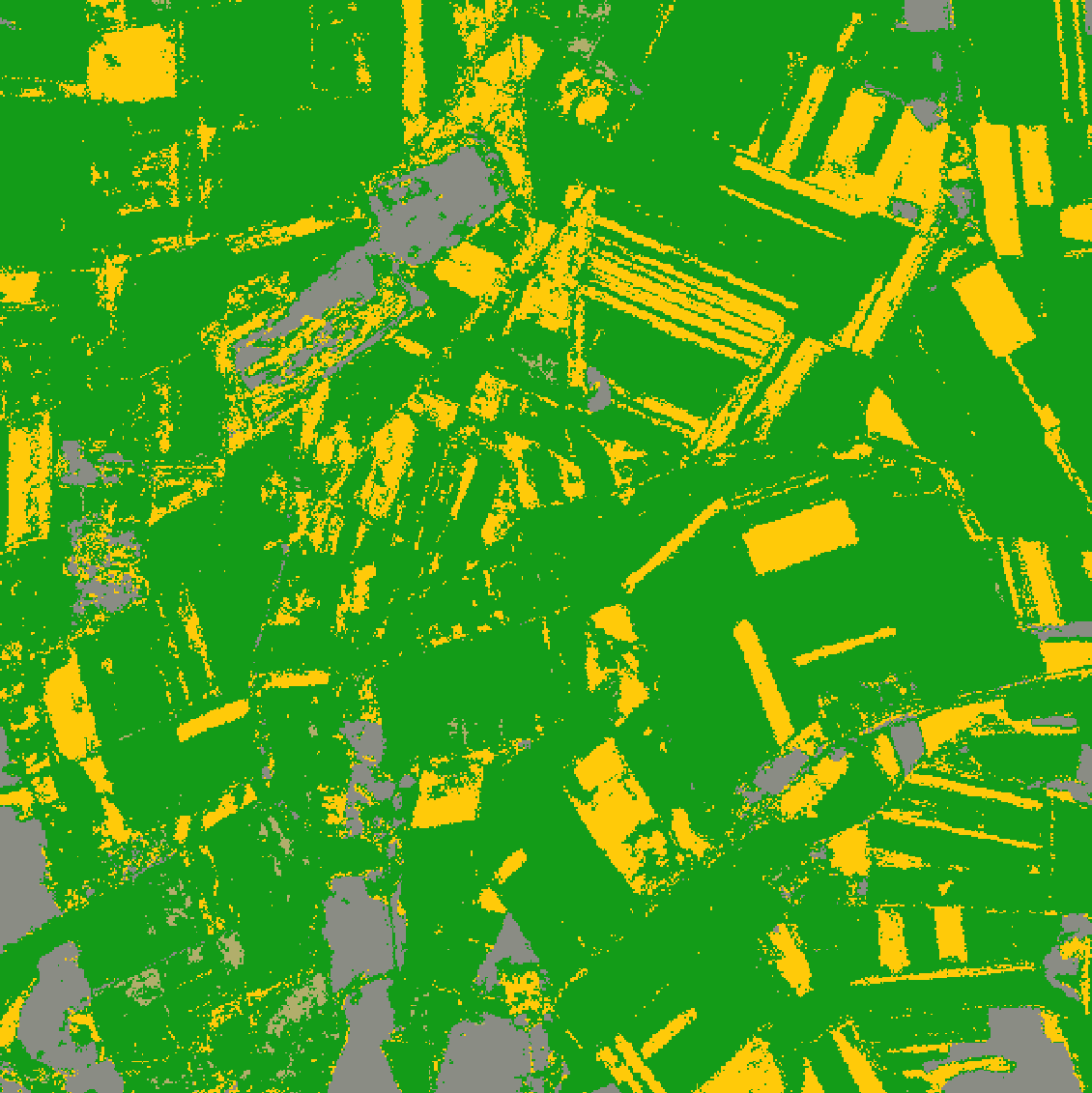

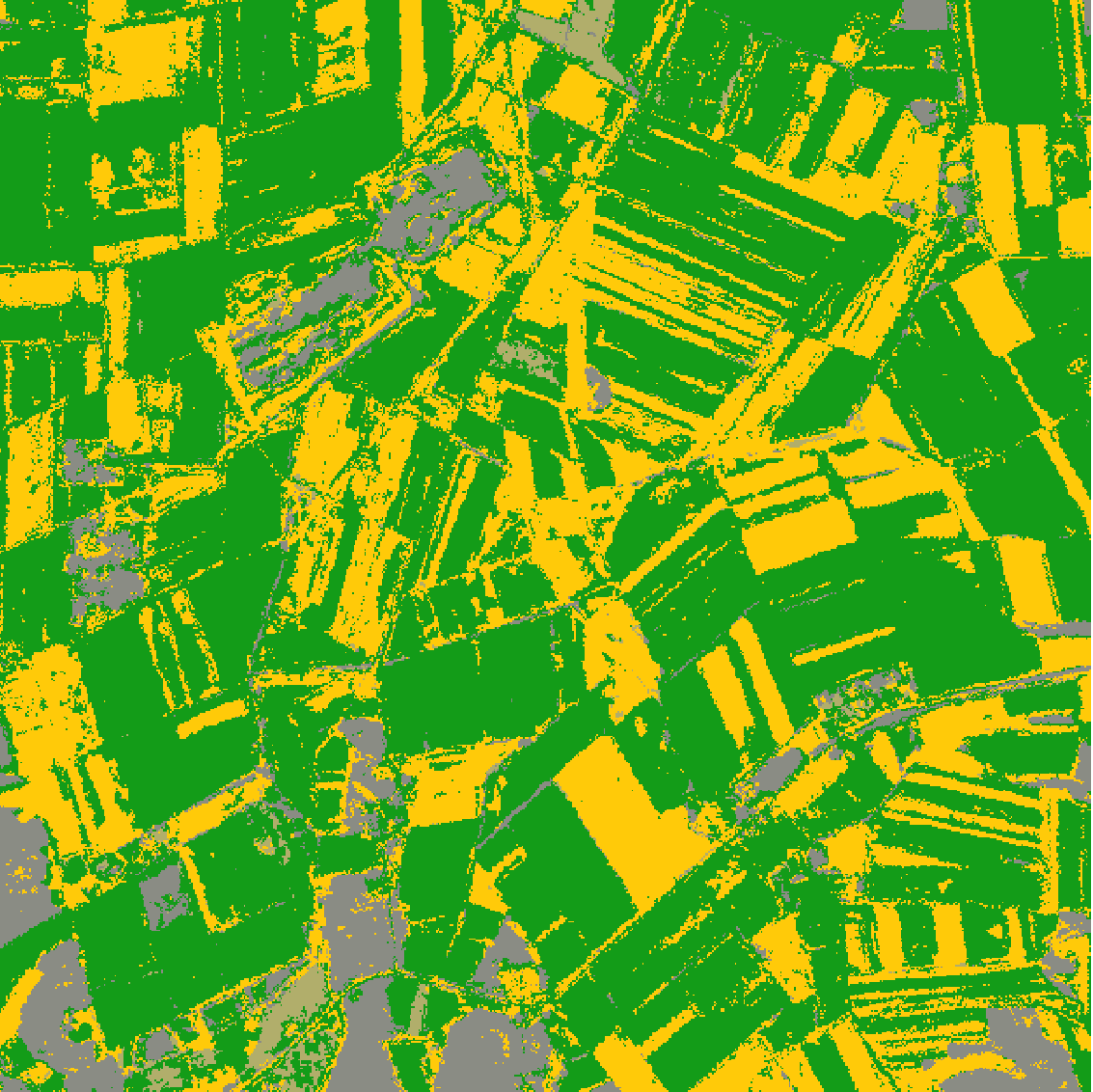


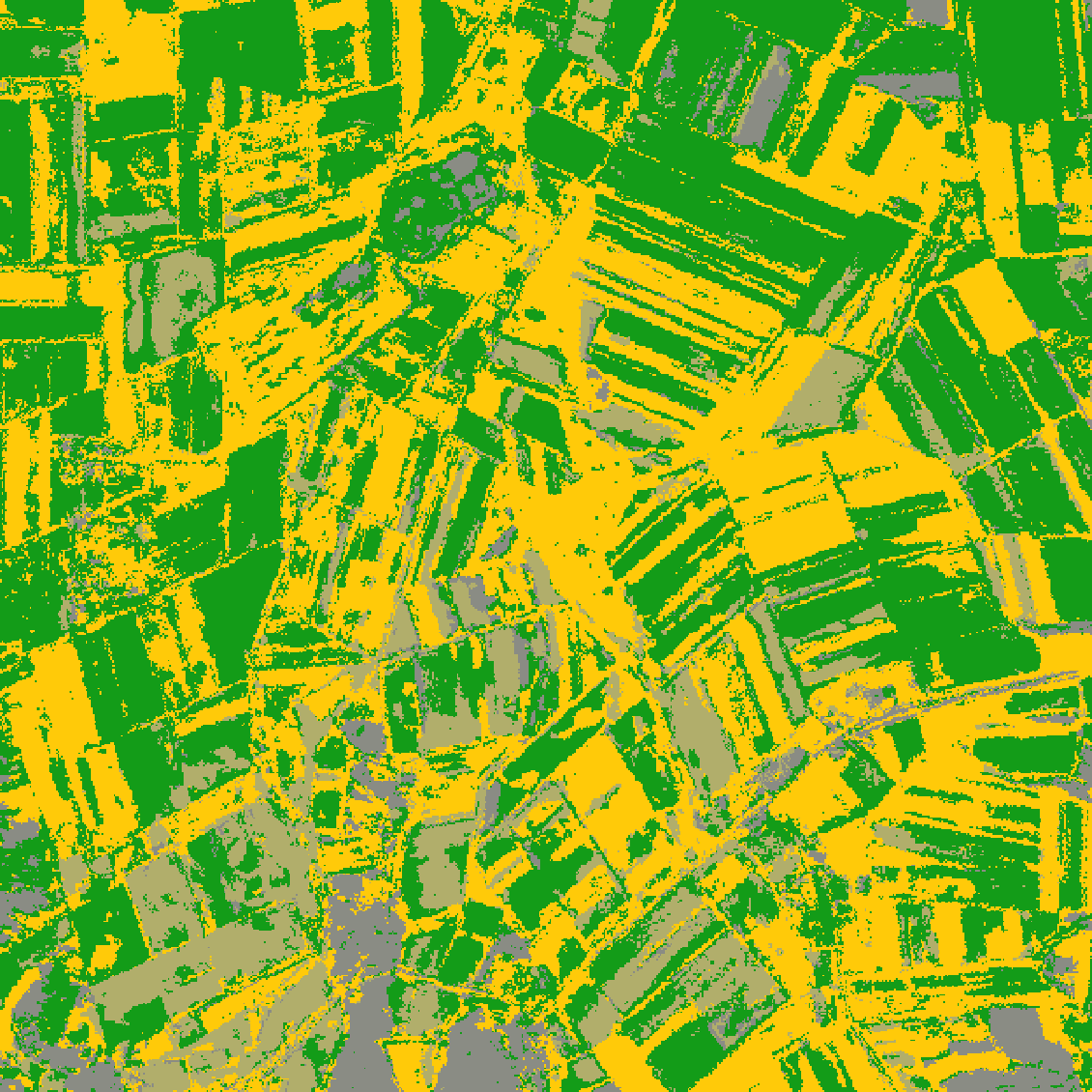

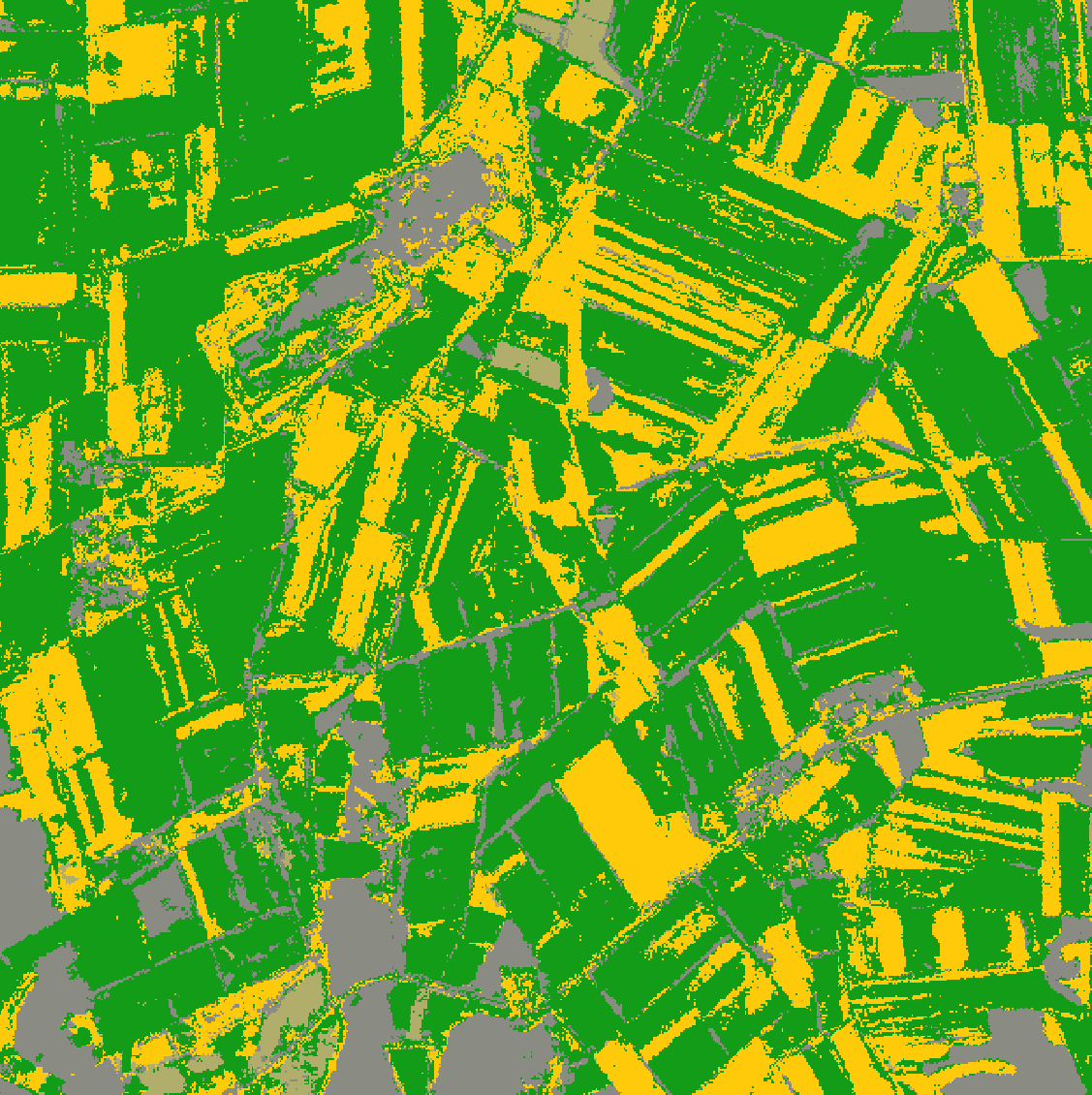

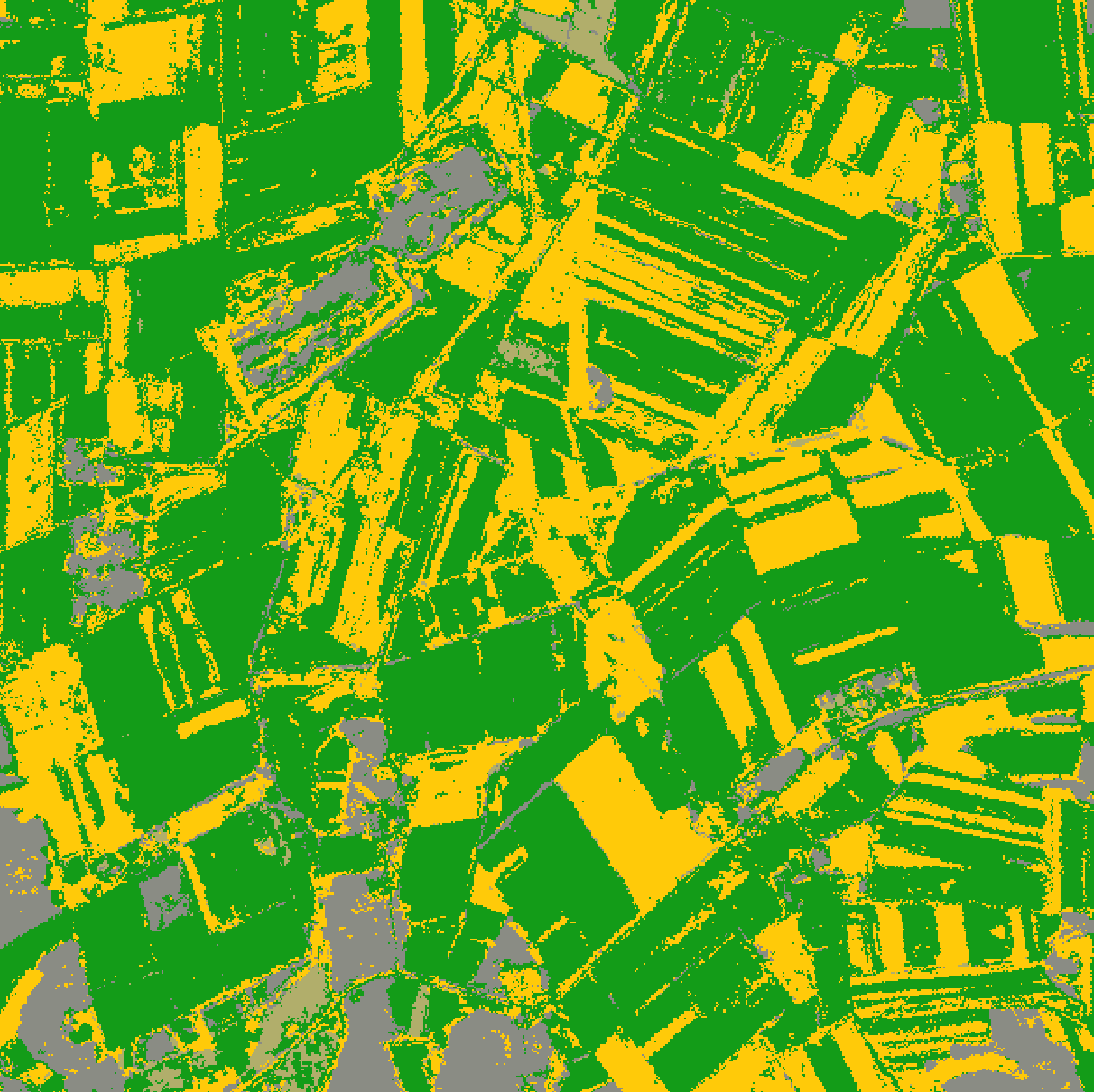


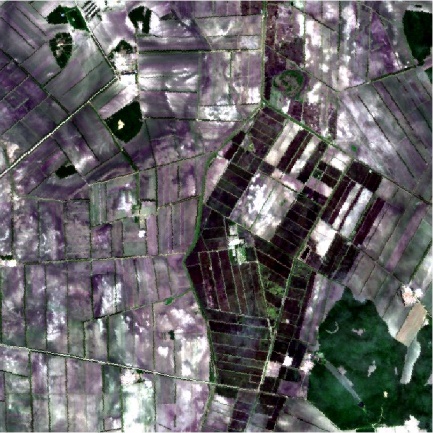

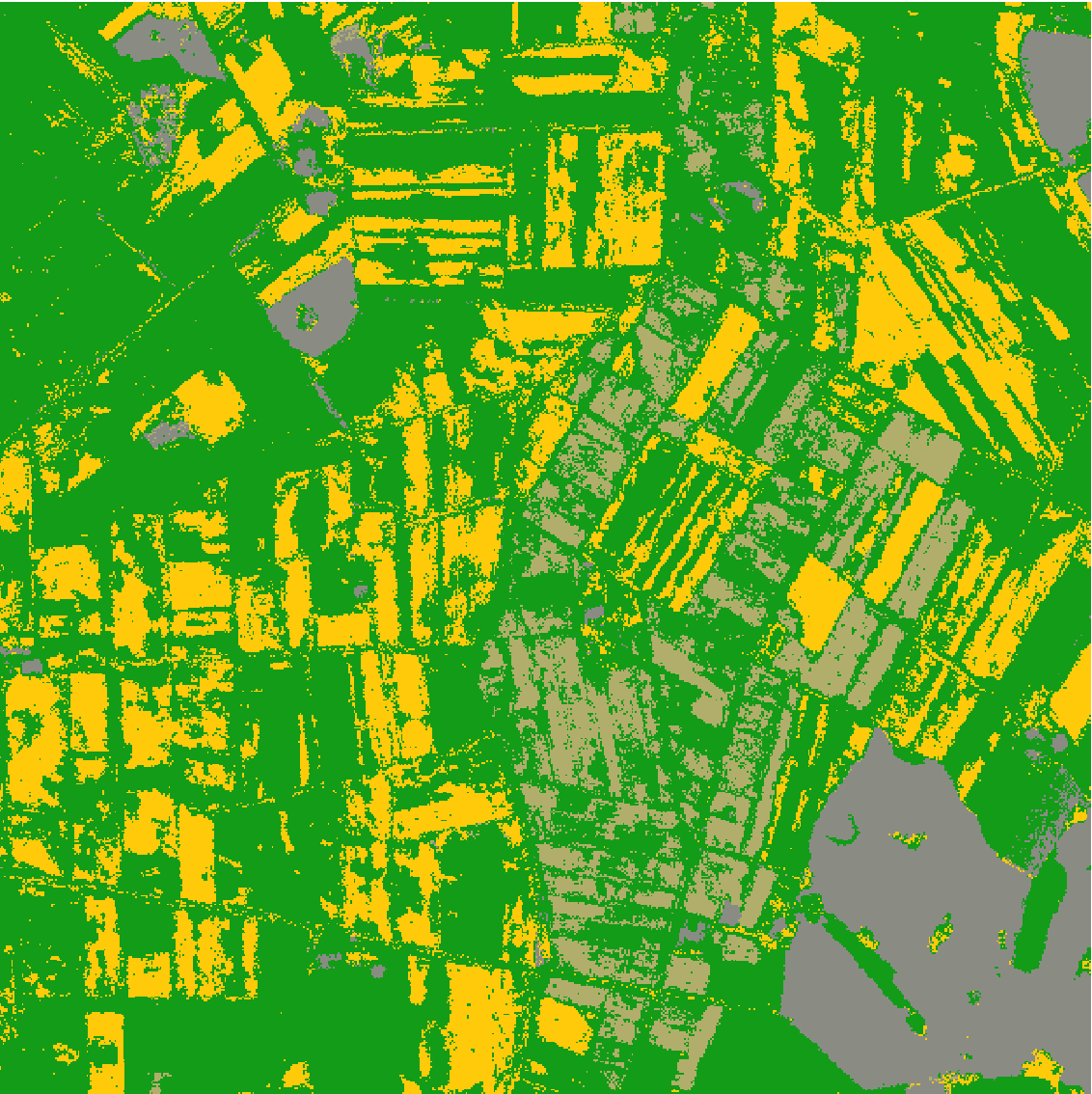

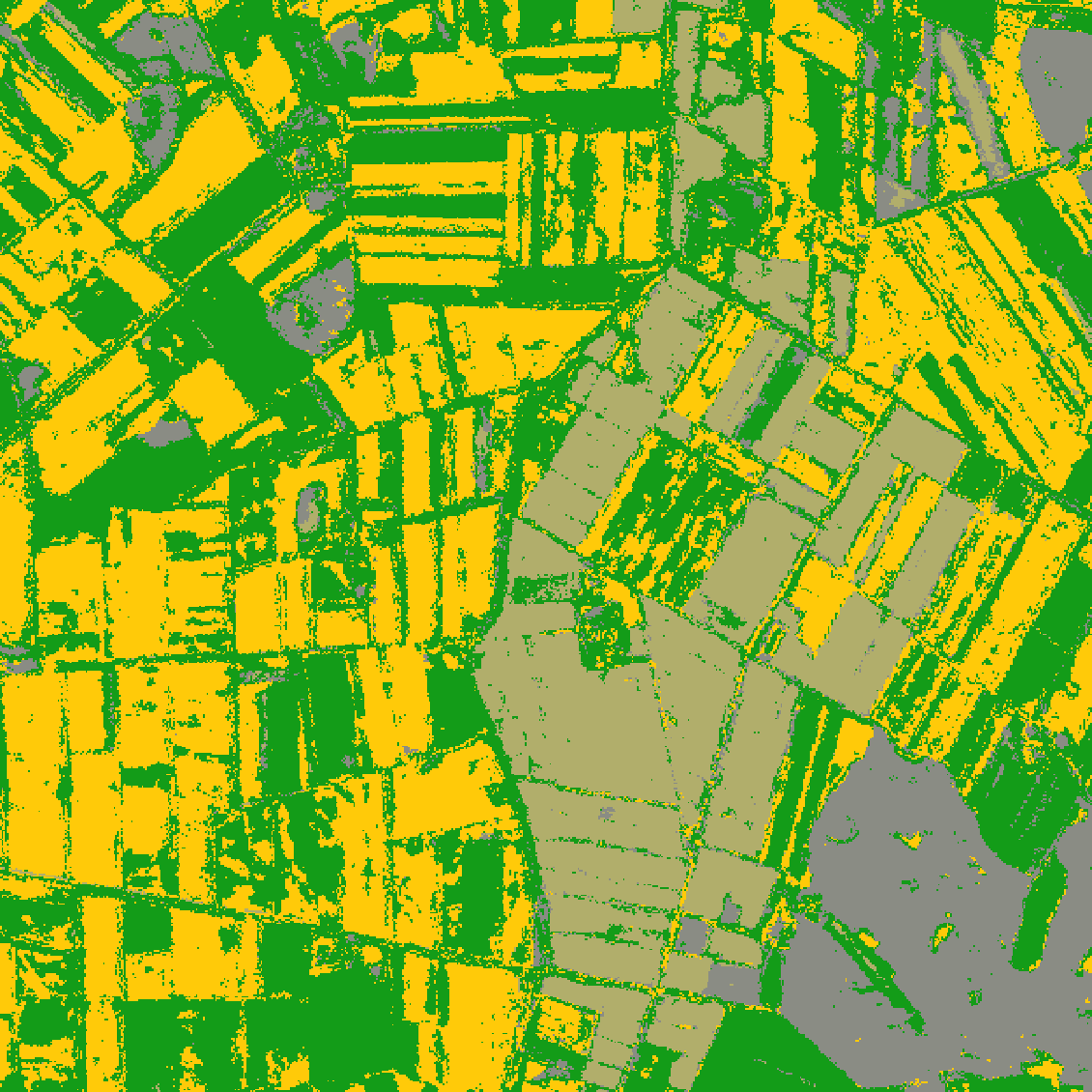


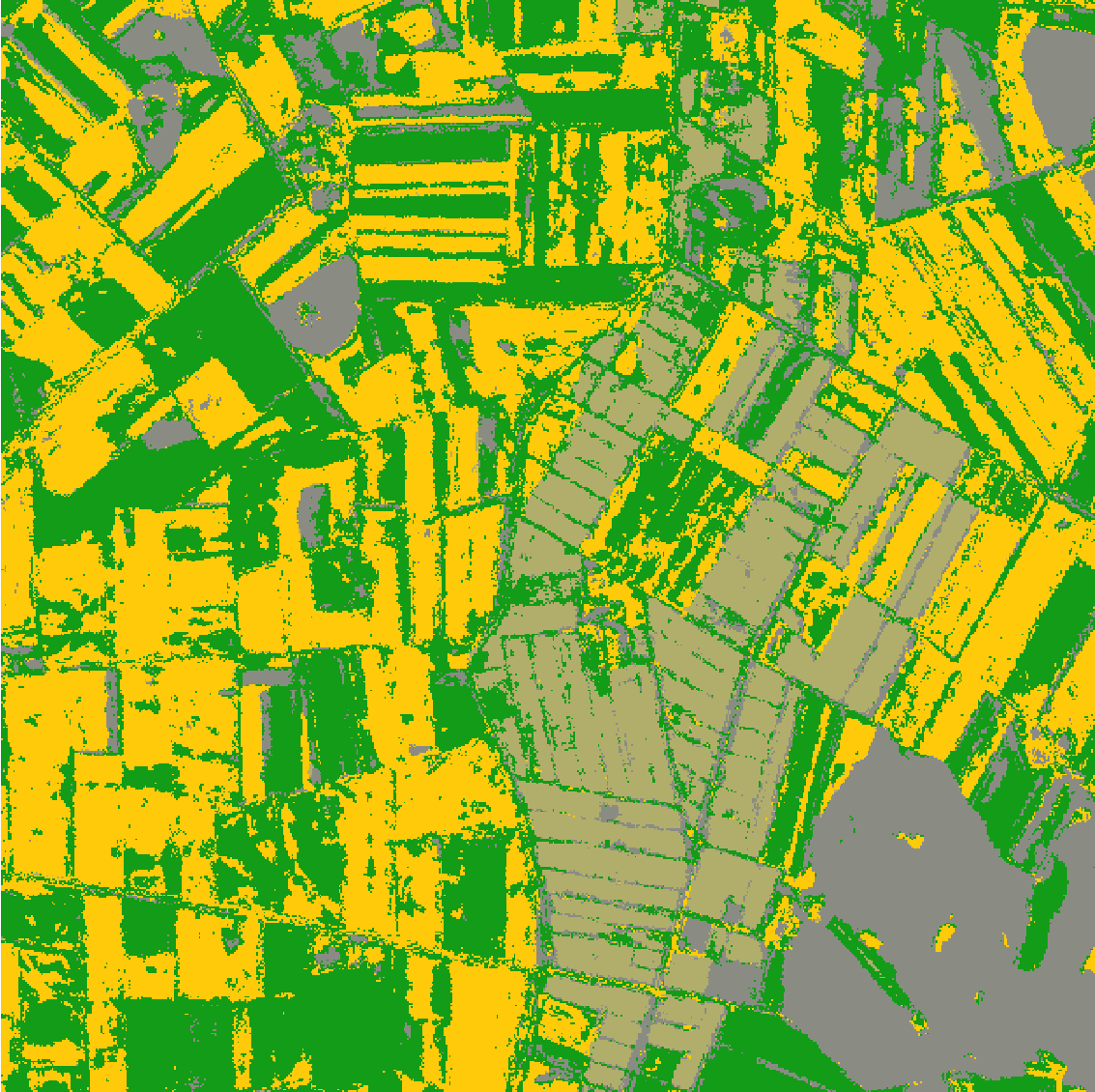

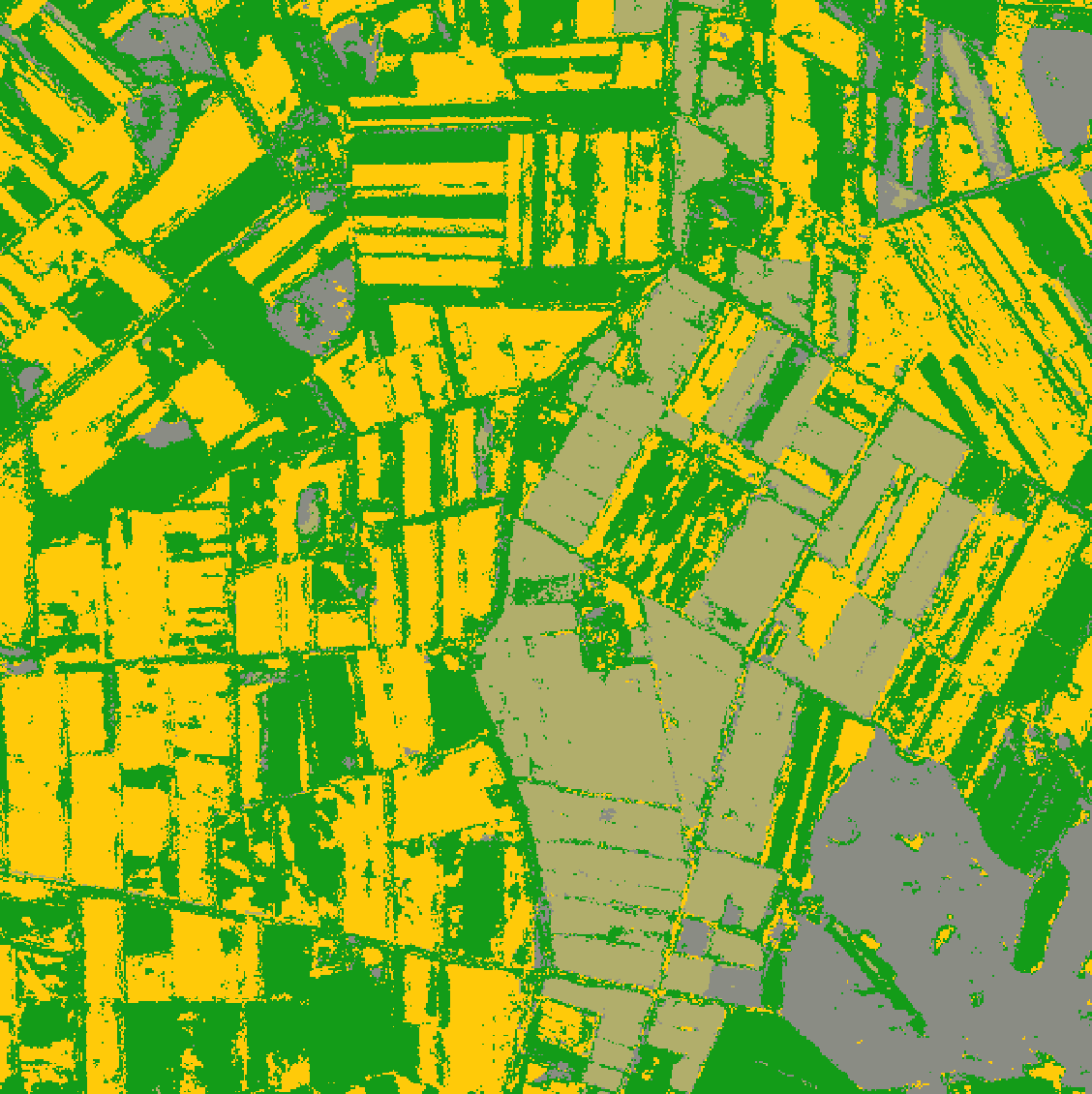

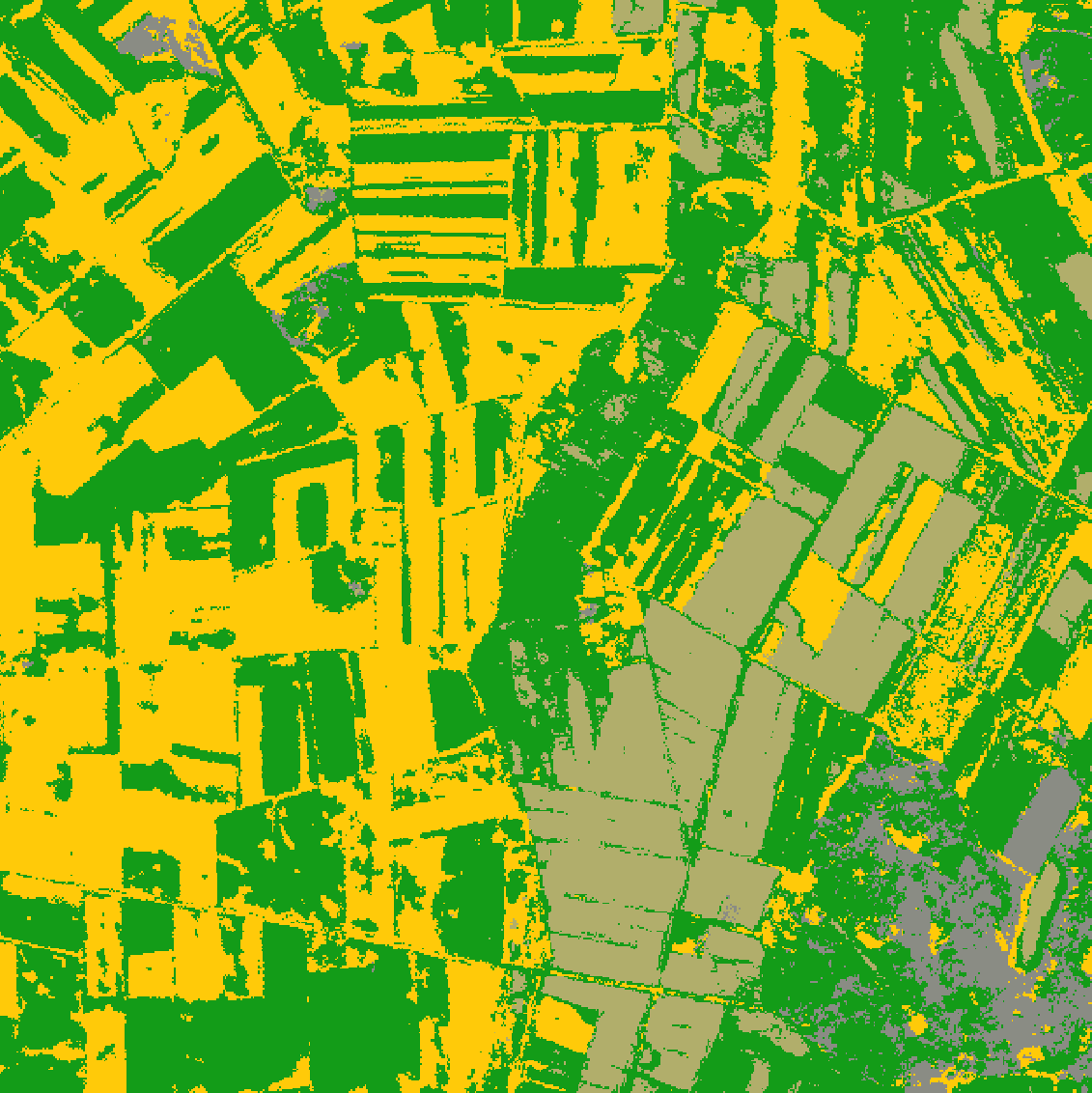


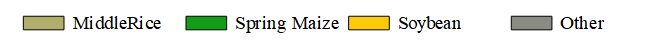


1. Eastern Heilongjiang

**Supplementary Figure 1.** Map of crop distribution in selected areas of the five study areas. For each study area, from left to right, remote sensing images, RF mapping, Performer mapping, ALBERT mapping, BERT mapping, and Cropformer mapping
